# Chemical Diversity and Bioactivities of Monoterpene Indole Alkaloids (MIAs) from Six Apocynaceae Genera

**DOI:** 10.3390/molecules26020488

**Published:** 2021-01-18

**Authors:** Afrah E. Mohammed, Zainab H. Abdul-Hameed, Modhi O. Alotaibi, Nahed O. Bawakid, Tariq R. Sobahi, Ahmed Abdel-Lateff, Walied M. Alarif

**Affiliations:** 1 Department of Biology, Faculty of Science, Princess Nourah Bint Abdulrahman University, P.O. Box 84428, Riyadh 11671, Saudi Arabia; AFAMohammed@pnu.edu.sa; 2 Department of Chemistry, Faculty of Science, King Abdulaziz University, P.O. Box 80203, Jeddah 21589, Saudi Arabia; flower9@hotmail.com (Z.H.A.-H.); nbawaked@kau.edu.sa (N.O.B.); drtariq_s@hotmail.com (T.R.S.); 3 Department of Natural Products and Alternative Medicine, Faculty of Pharmacy, King Abdulaziz University, P.O. Box 80260, Jeddah 21589, Saudi Arabia; ahmedabdellateff@gmail.com; 4 Department of Pharmacognosy, Faculty of Pharmacy, Minia University, Minia 61519, Egypt; 5 Department of Marine Chemistry, Faculty of Marine Sciences, King Abdulaziz University, P.O. Box 80207, Jeddah 21589, Saudi Arabia

**Keywords:** Apocynaceae, monoterpene, alkaloids, cytotoxicity, anti-inflammatory, antimicrobial

## Abstract

By the end of the twentieth century, the interest in natural compounds as probable sources of drugs has declined and was replaced by other strategies such as molecular target-based drug discovery. However, in the recent times, natural compounds regained their position as extremely important source drug leads. Indole-containing compounds are under clinical use which includes vinblastine and vincristine (anticancer), atevirdine (anti-HIV), yohimbine (erectile dysfunction), reserpine (antihypertension), ajmalicine (vascular disorders), ajmaline (anti-arrhythmic), vincamine (vasodilator), etc. Monoterpene Indole Alkaloids (MIAs) deserve the curiosity and attention of researchers due to their chemical diversity and biological activities. These compounds were considered as an impending source of drug-lead. In this review 444 compounds, were identified from six genera belonging to the family Apocynaceae, will be discussed. These genera (*Alstonia*, *Rauvolfia*, *Kopsia*, *Ervatamia*, and *Tabernaemontana,* and *Rhazya*) consist of 400 members and represent 20% of Apocynaceae species. Only 30 (7.5%) species were investigated, whereas the rest are promising to be investigated. Eleven bioactivities, including antibacterial, antifungal, anti-inflammatory and immunosuppressant activities, were reported. Whereas cytotoxic effect represents 47% of the reported activities. Convincingly, the genera selected in this review are a wealthy source for future anticancer drug lead.

## 1. Introduction

Alkaloids are basic nitrogenous natural metabolites with structural diversity and molecular conformity. They displayed interesting bioactivities and are known to perform an important role in plant protection. The majority of them were discovered from plants and recently recorded Ca 21,000 [1,2]. The alkaloids are generally derived from amino acids that are containing one or more nitrogen atoms. These precursors are playing a rule in their classification. Also, the biosynthetic pathway of alkaloids can be named according the amino acid source [3]. Thus, they can be categorized into several groups based on associated moieties, including piperidine, pyrrolidine, pyrrole, pyridine, quinolone, isoquinoline, indole, quinolizidine, pyrrolizidine, tropane, benzylisoquinoline, purine, β-carboline, indolinics and quinolizidine.

Terpenoids are considered to be interesting natural products that have chemical diversity and different bioactivities. Common terpenoids have been reported from marine sources [4]. Whereas, the plants were listed as an important source of such metabolites. Terpenoids include several subclasses according to the number of carbo-skeleton; monoterpenes (C_10_), sesquiterpenes (C_15_), diterpenes (C_20_), sesterterpenes (C_25_), triterpenes (C_30_), and tetraterpenes (C_40_). 

**Figure 1:**
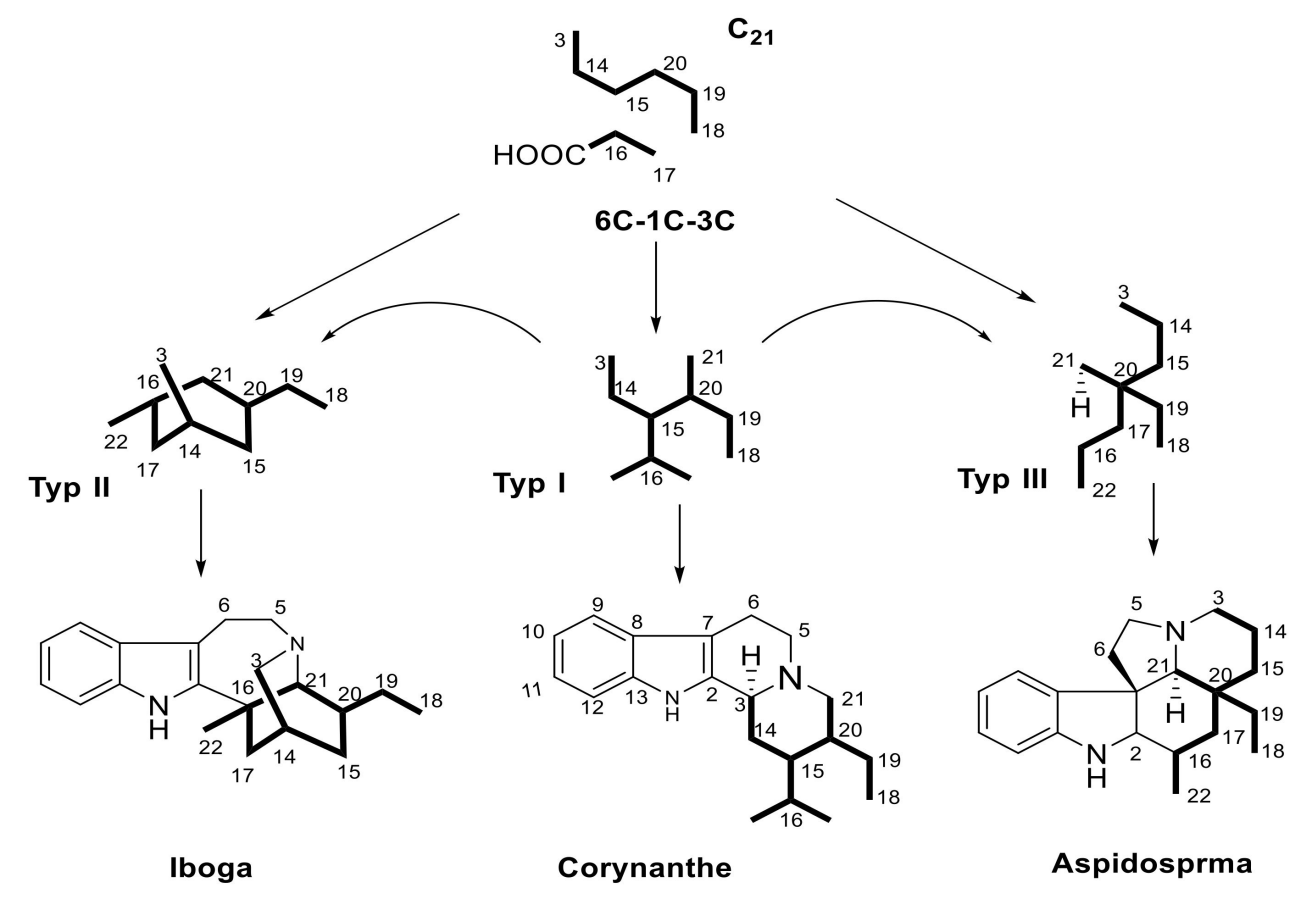
Biogenetic numbering rule as adopted from LeMen and Tylor.

Monoterpene indole alkaloids (MIAs) are metabolites containing a bicyclic structure of a benzene ring fused to a five-membered pyrrole ring. It is a noteworthy that the occurrence of multipart alkaloids is largely restricted to limited number of plant families. (e.g., Apocynaceae, Loganiaceae, and Rubiaceae) [5,6,7,8]. These families are closely taxonomically related. Also, on the chemical aspect, they are recognized to have apparent uniformity in the building blocks of these alkaloids. MIAs have been proposed to be sourced from strictosidine, which originates from the condensation of tryptophan with secologanin (C_10_ or C_9_ part), which can be divided into linear six carbon (6 C), one carbon (1 C) and three carbon (3 C) units (Figure 1). The connection between them requires proving. The nine-carbons fragment may be formed by the loss at certain stage of one of the carbons from the 3 C unit, and there are also a few indole bases which appear to have ended up without the 3 C or the 1 C units. Three hypothetical building blocks, Types I, II and III. It is nevertheless a useful way of dividing indole alkaloids into groups based on their sub­ architecture. Since Type I alkaloids are by far the most numerous, they may be the source of Type II and III. It was suggested by LeMen and Tylor that the convention be extended to cover Type II and III alkaloids as illustrated in Figure 1. On these hypothetical bases, the MIAs categorized according to their biogenic pathway in three main groups, corynanthe, aspidosperma and iboga [9].

Recently, strictosidine has been considered as the building block of MIAs biosynthesis [10]. MIAs have been proposed to arise from strictosidine, which itself originates from the condensation of tryptophan with secologanin in a 1:1 ratio. Strictosidine has been elaborated to give an impressive array of structural variants. This type of alkaloids possess 18 (or 19) carbon atoms on its skeleton. Additionally, the MIAs could be produced from tryptophan and secologanin in 1:2 or 2:1 ratio. According to this arrangement, three types (classes) of monoterpenes were constructed, including, corynanthe (e.g., ajmalicine), aspidosperma (e.g., tabersonine) and iboga (e.g., catharanthine) [11,12,13].

**Figure 2:**
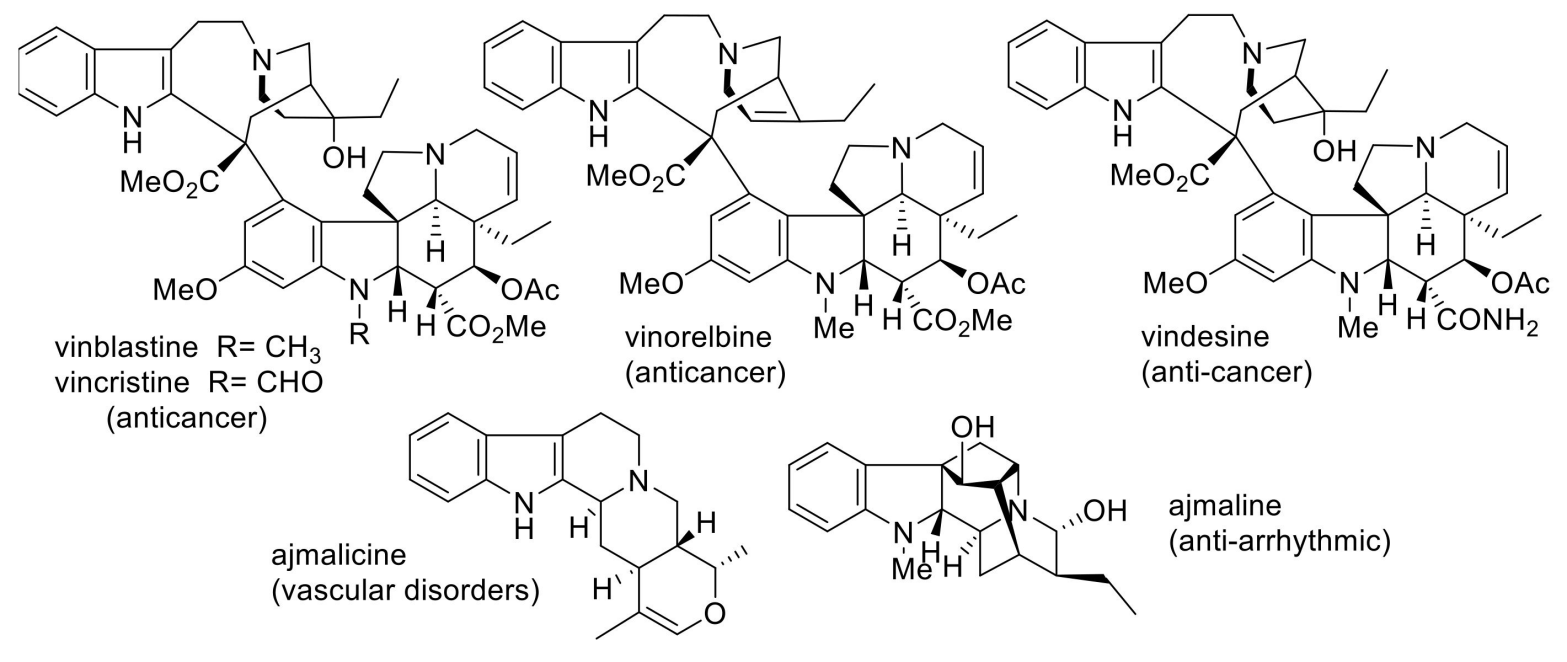
Examples of well-known biologically active terpene indole alkaloids.

Apocynaceae contains about 250 genera and 2000 species [14]. Five sub-families are classified under Apocynaceae, including, Apocynoideae, Asclepiadoideae, Periplocoideae, Rauvolfioideae, and Secamonoideae. Apocynaceae species ranged from shrubs to trees. The characteristic features of these plants include colorful flowers and opposite leaves. Traditionally, species of this family have been used for the treatment of fever, malaria, gastrointestinal ailments, diabetes, and pain [15]. Additionally, some species have shown antiplasmodial and anticancer activities [14]. Several Apocynaceae MIAs have been used as anticancer, analgesic, anti-inflammatory and anti-spasmodic agents. For example, vinblastine, vinorelbine, vincristine, and vindesine were utilized as anticancer agents, whereas ajmalicine and ajmaline were used in the treatment of cardiovascular disorders (Figure 2) [2]. *Catharanthus roseus* and *Rauvolfia serpentine* are members of Apocynaceae and are known as sources of bioactive indole alkaloids [16]. Reserpine has been used as a tranquillizer, whereas vinblastine and vincristine have been used as anti-leukemic agents [17]. Vincristine and vinblastine were among the earliest anti-tumor agents, and since 1965 have been used as tubulin polymerization inhibitors. They have been used in combination for the treatment of acute lymphoblastic leukemia and also against both Hodgkin’s and non- Hodgkin lymphoma. Additionally, strychnine is potent muscle contracting agent whereas, yohimbine has been used for the treatment of sexual dysfunction and investigated as a remedy for type-2 diabetes in animal and human models.

**Figure 3:**
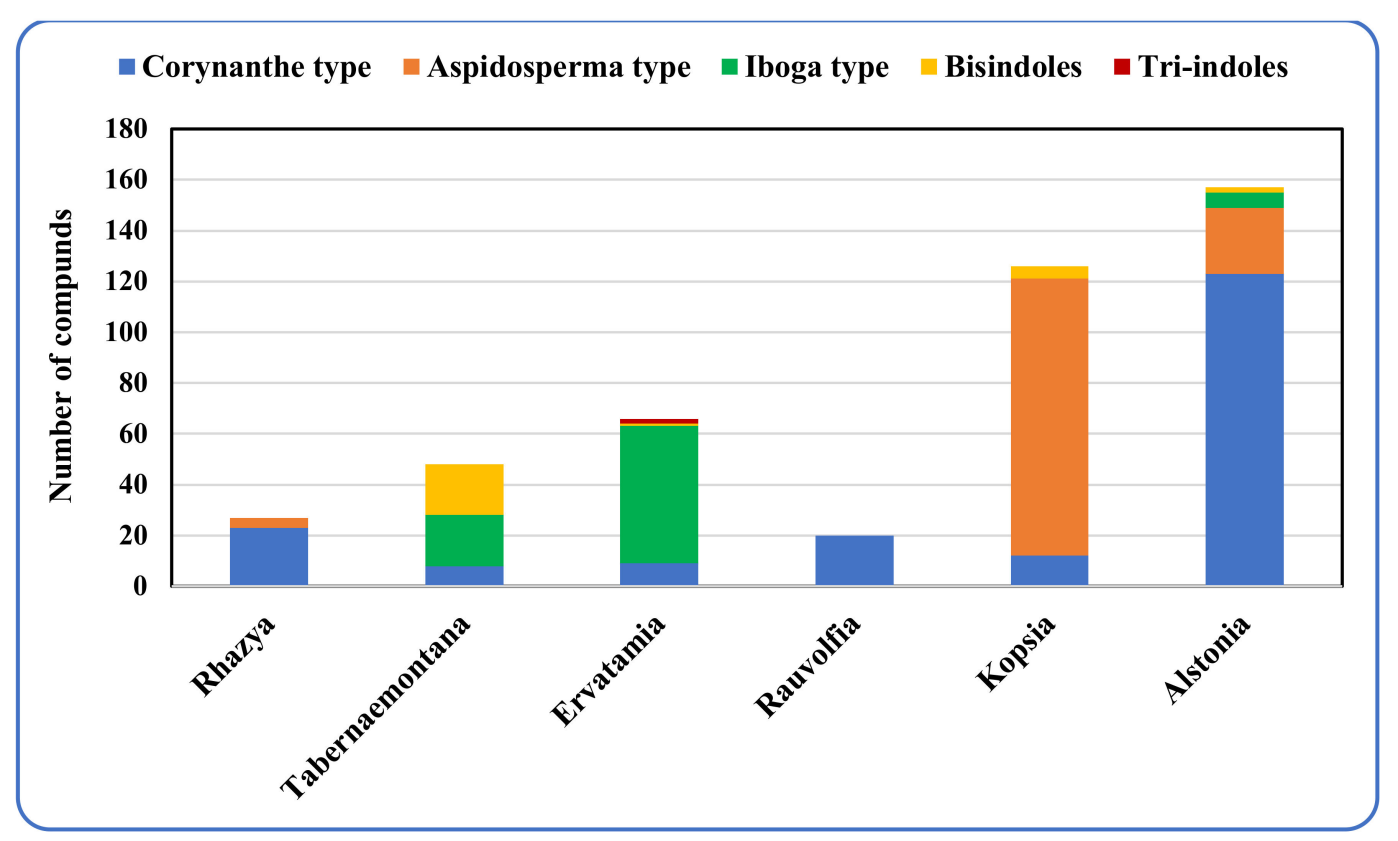
The types of the structures identified monoterpenoid alkaloids from the six genera.

**Figure 4:**
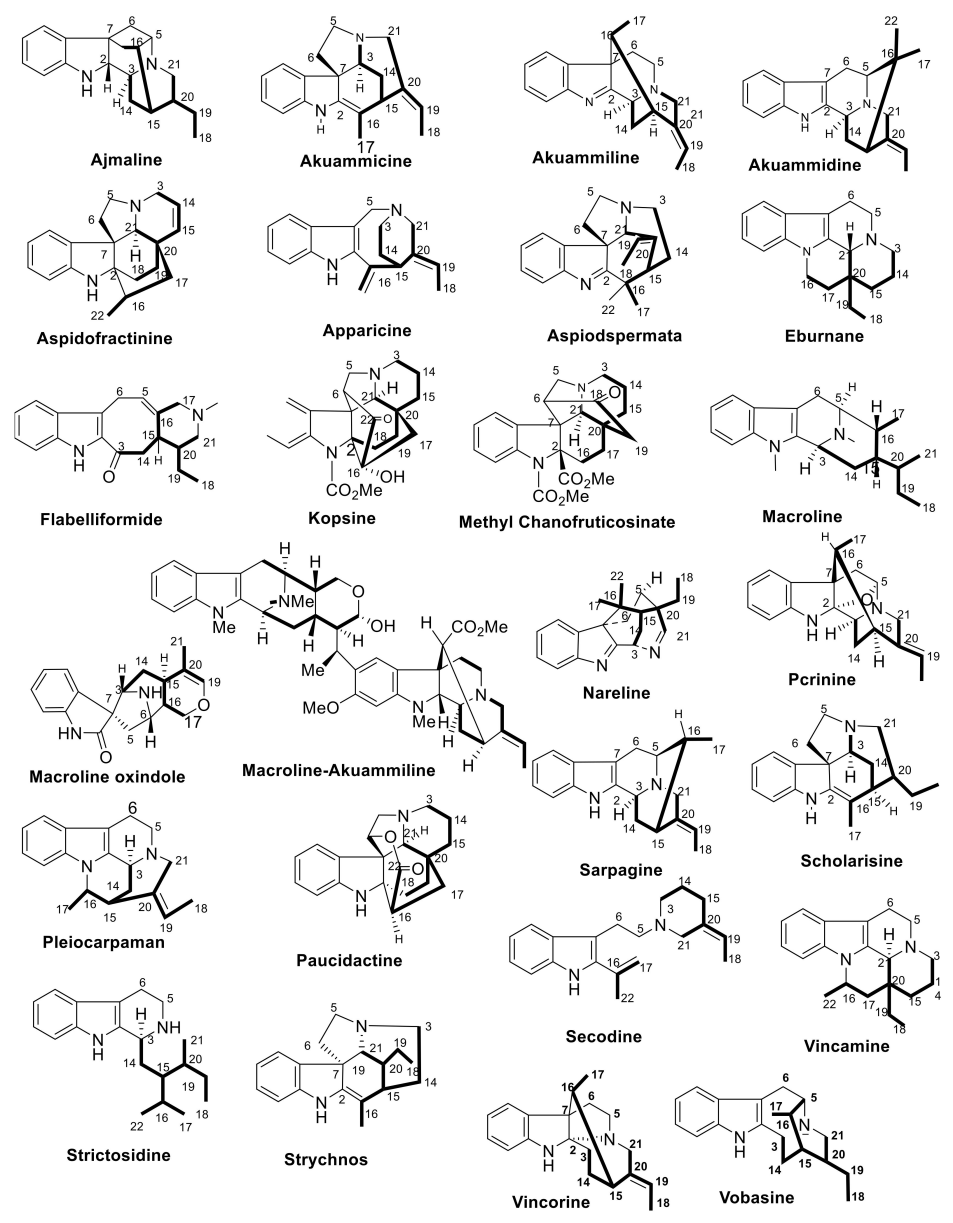
Common monoterpenoid indole alkaloidal skeletons of the six genera.

There are several publications interested in the terpene indole alkaloids of individual species of the family Apocynaceae. The current review organizes the reported MIAs considering the historical aspect in each selected genus. Moreover, these MIAs were biosynthetically classified according to the tepenoidal fragment, i.e., corynanthe, aspidosperma, or iboga. Also, it focuses on the origin, structural diversity and biological activities exerted by 444 (Table 1) monoterpene indole alkaloids which have been reported from selected six genera of the family Apocynaceae (*Alstonia*, *Kopsia*, *Ervatamia*, *Rauvolfia*, *Tabernaemontana* and *Rhazya*), in the period between 2010 and December 2020. The listed metabolites are categorized under 26 subclasses, ajmaline, akuamiline, akuammidine, akuammicine, apparicine, aspidofractinine, aspidospermatan, eburnane, flabelliformide, kopsine, macroline, macroline oxindole, macroline-akuammiline, methyl chanofruticosinate, nareline, paucidactine, picrinine, pleiocarpamine, sarpagine, scholaricine, secodine, strictosidine, strychnos, vincamine, vincorine and vobasine (Figure 3 and Figure 4). 

Additionally, the future prospective and emphasizing the research gaps and highlighting the roadmap to discover the potent bioactive monoterpenoid alkaloids, which could be a drug lead from the six genera. Also, this review will discuss the reported structural activity relationships.

## 2. Alstonia

Plants of the genus *Alstonia* are grown in Africa and Asia. It includes 60 species, which were recognized as rich source of heterocyclic monoterpene indole alkaloids. It has different names according to the geographical sources, including Devil tree, Australian fever bush, dita bark, Australian quinine, fever bark and palimara. *Alstonia* bark shows potent therapeutic effects including anti-inflammatory, antirheumatic, analgesic, antidiabetic, antimalarial, antipyretic, antihelminthic, antibiotic, antimicrobial, anticancer, antibacterial and antitussive effects [18,19,20]. 

**Figure 5:**
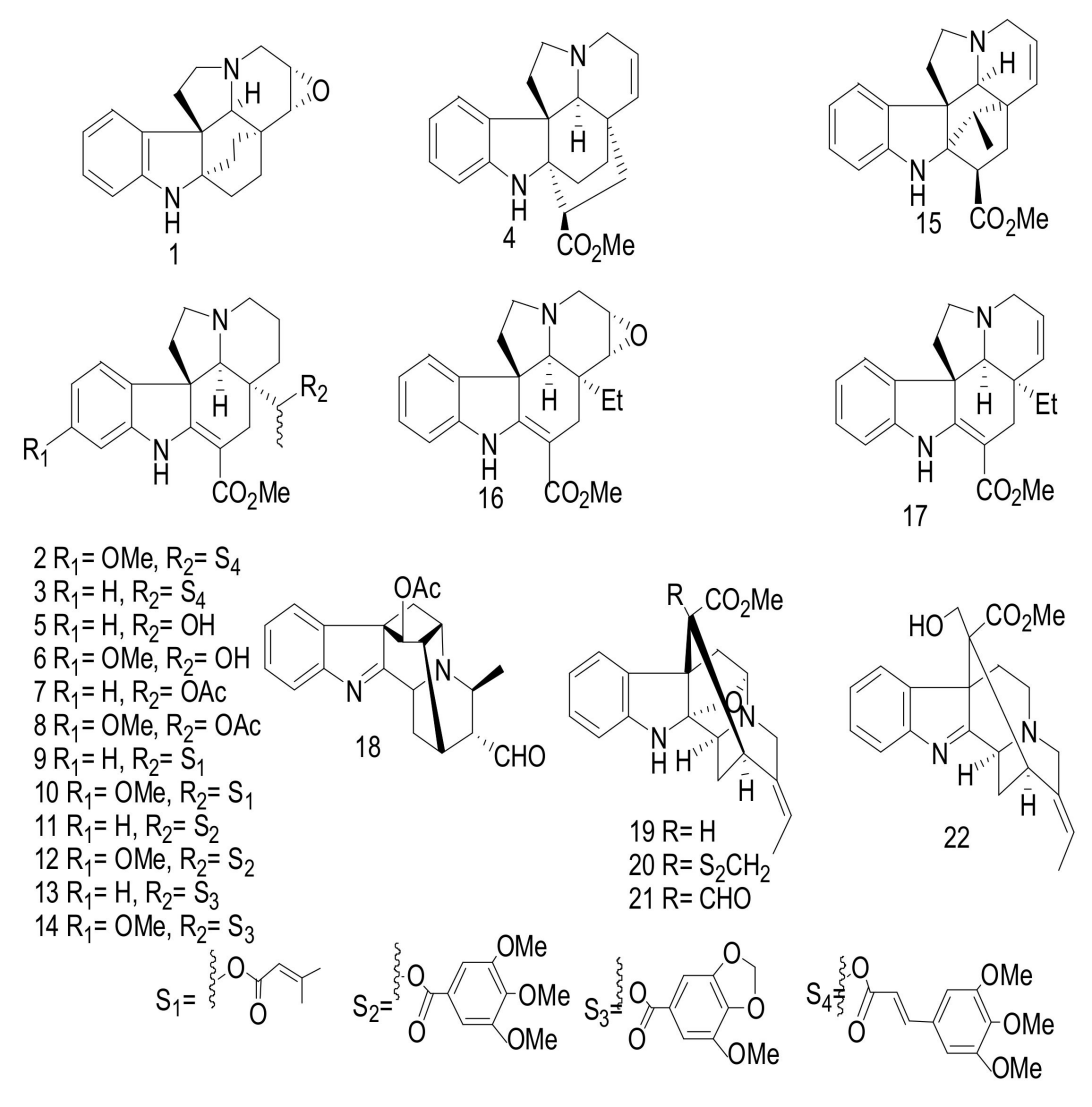
Compounds **1**–**22**.

Three monoterpene indole alkaloids (MIAs) derivatives, (14α,15α)-14,15-epoxyaspidofractinine (**1**) and maireines A (**2**) and B (**3**) have been isolated from the leaves and twigs of *A. mairei* [21]. Additionally, venalstonine (**4**) [22], (−)-minovincinine (**5**) [23], (−)-11-methoxyminovincinine (**6**) [24], (−)-echitovenine (**7**) [25], echitovenaldine (**8**) [26], echitovenidine (**9**), 11-methoxyechitovenidine (**10**) [27], echitoveniline (**11**), 11-methoxyechitoveniline (**12**) [24], echitoserpidine (**13**) [28],11-methoxyechitoserpidine (**14**) [29], (19*S*)-vindolinine (**15**) [22], lochnericine (**16**), tabersonine (**17**) [30], perakine (**18**) [31], picrinine (**19**) [32], F (**20**) [33], picralinal (**21**) [34] and rhazimol (**22**) [35] were isolated from the same species (Figure 5). These compounds were elucidated through the interpretation of different spectroscopic measurements including 1D and 2D NMR and MS. Interesting in compound (**1**) was the interpretation of the Rotating Frame Overhauser Enhancement Spectroscopy (ROSY) spectrum led to the establishment of the α-orientation of the epoxy moiety. Compounds **1**–**22** were evaluated against five human cancer cells, hepatocellular carcinoma (SMMC-7721), breast (SK-BR-3), pancreatic (PANC-1), human myeloid leukemia (HL-60), and lung (A-549) with IC_50_ values > 40 μM [21].

The majority of reported alkaloids from *A. scholaris*, were of the picrinine type whereas, those isolated from *A. yunnanensis* were either picrinine or aspidospermine types.

**Figure 6:**
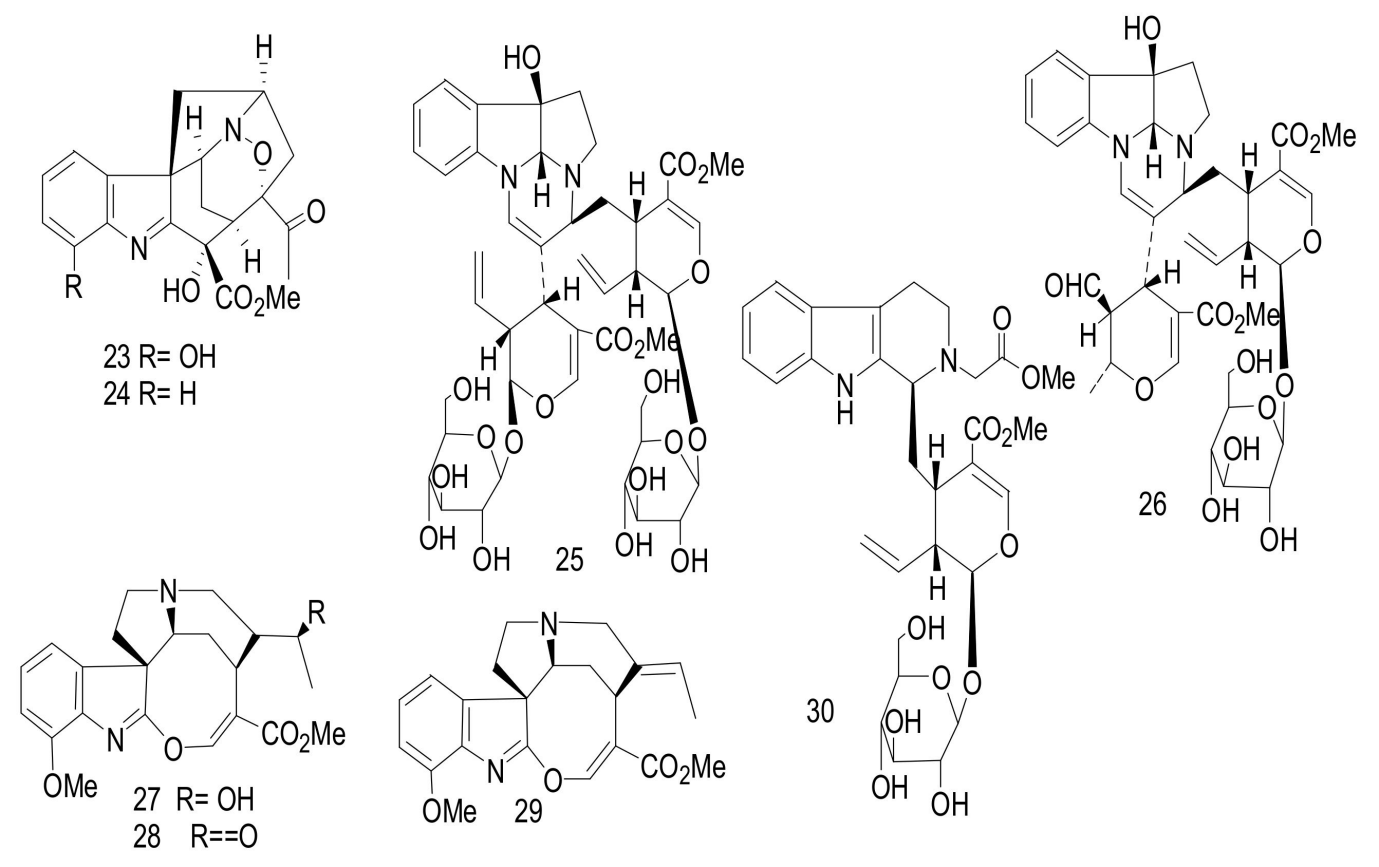
Compounds **23**–**30**.

Alsmaphorazines A (**23**) and B (**24**) (Figure 6) were identified from the leaves of malaysian *A. pneumatophore*. The chemical structures were determined on the basis of 2D NMR and MS spectral analysis. These compounds had an unprecedented skeleton containing an 1,2-oxazine (six-member ring) and an isoxazolidine (five-member ring) [36]. The absolute configuration of alsmaphorazine B was determined using CD spectral analysis. The absolute configuration of alsmaphorazine B (**24**) was studied by comparing its experimental CD spectrum with the calculated CD spectrum, with the CD calculations performed by Turbomole 6.1using the Time-Dependent Density Functional Theory (TD-DFT-B3LYP/TZVPP) level of theory on RI-DFTBP386LYP/TZVPP optimized geometries. Compound **23** inhibited the production of nitric oxide (NO) in an LPS-stimulated J774.1 cell with an IC_50_ value = 49.2 μM, without affecting the cell viability, whereas compound **24** showed no inhibitory effect at 50.0 μM. Compound **23** was more potent as an anti-inflammatory agent due to the presence of a hydroxyl group at C-12 [36].

Alstrostines A (**25**) and B (**26**) were determined as derived from the condensation of tryptophan and secologanin in a ratio of 1:2. They were isolated from *Alstonia rostrata* [37]. The structures were established by measuring ^1^H, ^13^C, HSQC, HMBC, ^1^H-^1^H COSY and ROESY. Compounds, **25** and **26,** exhibited a weak cytotoxicity against five human cancer cells, hepatocellular carcinoma (SMMC-7721), breast (MCF-7), colon (SW480), myeloid leukemia (HL-60) and lung (A-549), with IC_50_ values > 40 μM [37].

Alstrostines C-F (**27**–**30**) (Figure 6) were isolated from the leaves and twigs of Chinese *A. rostrata* [38]. Compounds **27**–**30** showed a characteristic UV absorption at 326, 275 and 214 nm, which indicated the presence of an indole alkaloid with a β-anilineacrylate system. The chemical structure elucidation was confirmed by 1D and 2D NMR. Compounds **27**–**30** showed weak cytotoxicity against five human cancer cells, breast (SK-BR-3), human myeloid leukemia (HL-60), pancreatic (PANC-1), hepatocellular carcinoma (SMMC-7721) and lung (A-549) cells, with IC_50_ values > 40 μM [38].

**Figure 7:**
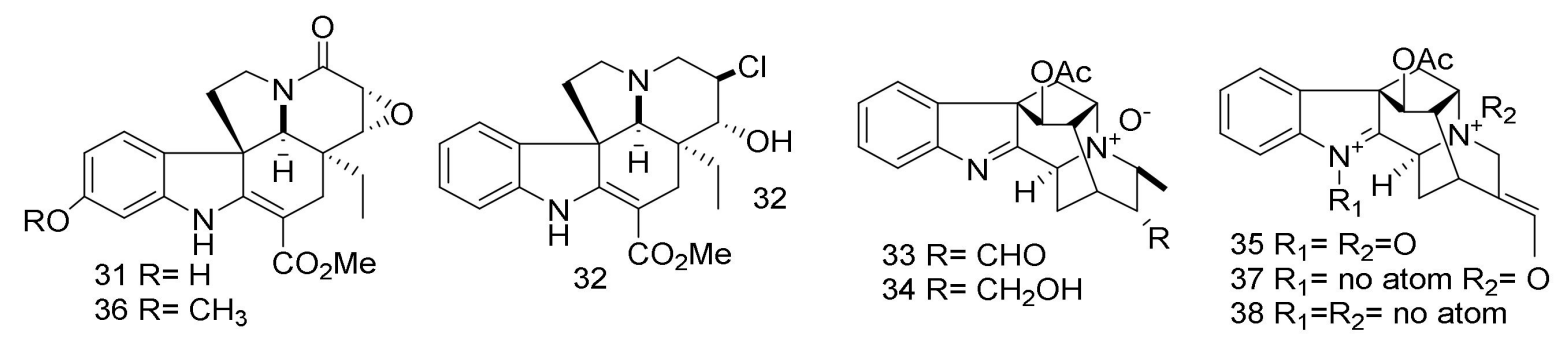
Compounds **31**–**38**.

Five MIAs, 11-hydroxy-6,7-epoxy-8-oxo-vincadifformine (**31**), 14-chloro-15-hydroxyvinca difformine (**32**), perakine N_4_-oxide (**33**), raucaffrinoline *N*_4_-oxide (**34**), and vinorine *N_1_*,*N*_4_-dioxide (**35**) (Figure 7) have been reported from *A. yunnanensis.* Additionally, three compounds, 11-methoxy-6,7-epoxy-8-oxovincadifformine (**36**), vinorine *N*4-oxide (**37**) and vinorine (**38**) have also been found from the same plant [39]. The chemical structures were established based on 1D and 2D (^1^H-^1^H-COSY, HMQC, HMBC, and ROESY) NMR spectroscopy. Compounds **33**, **34,** and **37** showed cytotoxicity against astrocytoma and glioma cells (CCF-STTG1, CHG-5, SHG-44 and U251) with IC_50_ values ranging from 9.2 to 17.4 μM. Adriamycin was used as positive control and showed cytotoxicity with an IC_50_ value ranging from 21.8 to 33.7 μM. These compounds exhibited a cytotoxic effect against breast cancer (MCF-7) and human skin cancer (SK-MEL-2) with IC_50_ values ranging from 28.1 to 35.5 μM. Adriamycin was used as positive control and exhibited a cytotoxic effect with IC_50_ values ranging from 14.1 to 37.6 μM [39]. Alkaloids **35** and 3**8** displayed no cytotoxic activities or selective inhibition of COX-2 comparable to those of **33**, **34** and **37** although they possess the same monoterpene indole skeleton. The observations indicated that a *N*_4_-oxide functionality was essential for cytotoxic and anti-inflammatory properties, while a *N*_1_-oxide maybe weaken the cytotoxic activities for this type of alkaloids. The observations indicated that the presence of oxide in *N*_4_ was essential for cytotoxic and anti-inflammatory activities, while the presence of the oxide on *N*_1_-oxide led to decreasing the cytotoxicity.

**Figure 8:**
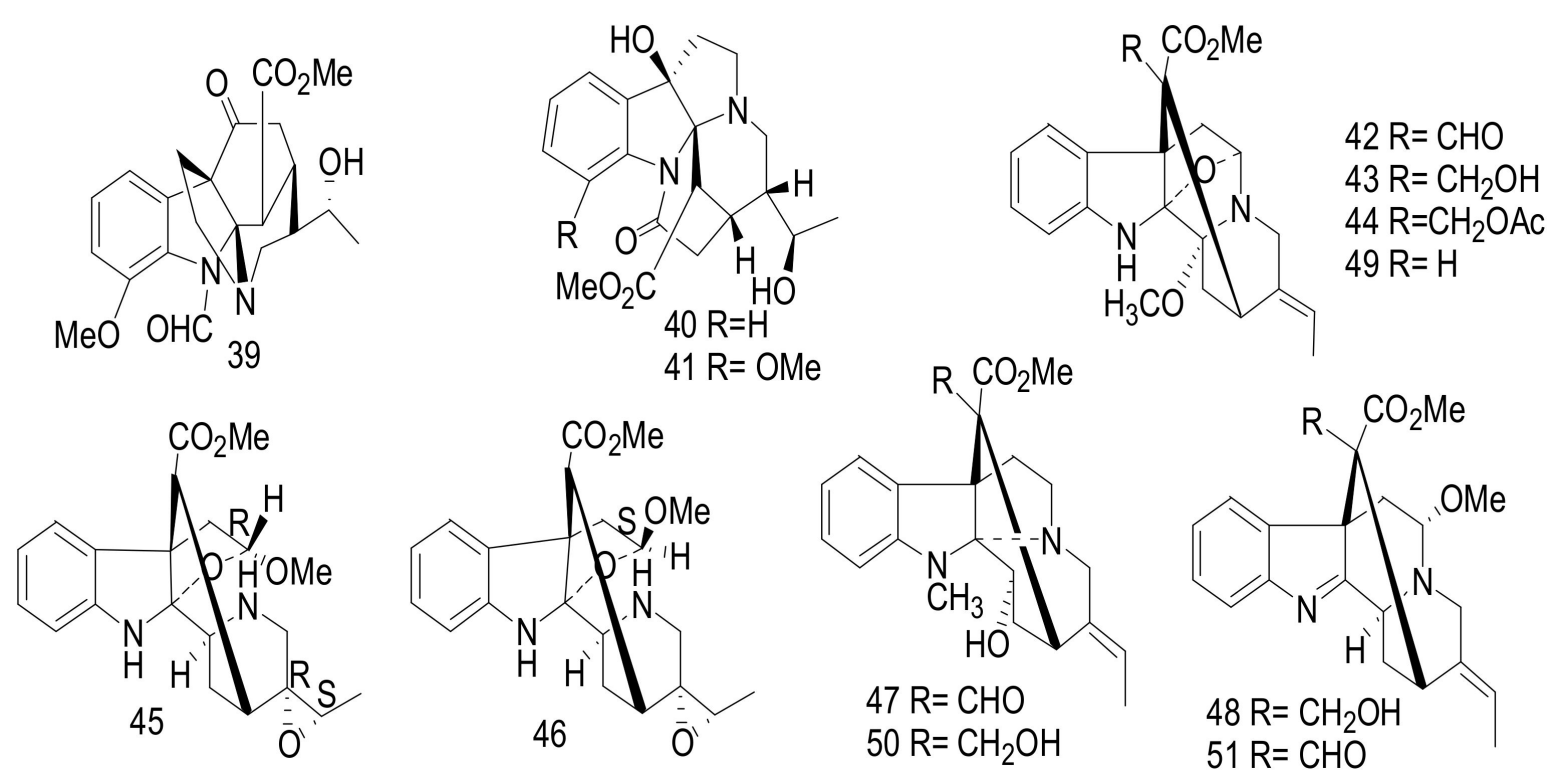
Compounds **39**–**51**.

Alsmaphorazines (C) (**39**), (D), (**40**), and (E) (**41**) (Figure 8) were elucidated from *A. pneumatophore* [40]. The planar structure of **39** was elucidated by 2D NMR and MS. This alkaloid possesses a novel ring skeleton containing an octahydropyrrolo[2,3-b]pyrrole unit. The absolute configuration of (**39**) was determined by the modified Mosher’s method and also confirmed by measuring the CD spectrum, which fully agreed with the CD calculations. Compounds **39**–**41** showed no cytotoxicity and also weak anti-melanogenesis activity against HL-60 and B16F10 cells with IC_50_ values >100 μM [40].

New scholarisins I-VII (**42**–**48**), and (3*R*,5*S*,7*R*,15*R*,16*R*,19*E*)-scholarisine F (**49**) [41], along with three known indoles: 3-*epi*-dihydrocorymine (**50**), and (*E*)-16-formyl-5α-methoxystrictamine (**51**) were identified from the leaves of *Alstonia rupestris* (Figure 8) [42]. Compounds **42**, **47**, and **51** showed significant cytotoxicity against cancer cells, A-549, BGC-823, HepG2, HL-60, MCF-7, SMMC-7721, and SW480 with IC_50_ values < 30 μM. These compounds exhibited selective inhibition effect of COX-2 with IC_50_ values ranging between 92.0 and 96.4 μM, while compounds **43**, **44**, and **48**–**50** displayed a weak cytotoxicity towards the tested tumor cells with IC_50_ values > 40 μM. Furthermore, alkaloids **45** and **46** showed a weak cytotoxicity with IC_50_ values > 80 μM. Doxorubicin was used as a positive control and showed with IC_50_ value < 35 µM. These activities of **45** and **46**, indicated that the bond connection between C-5 and N-4 was essential for the cytotoxicity [41]. Compounds **42**, **43**, **44** and **49** showed antifungal activity against *Gibberella pulicaris* (KZN 4207) and *Colletotrichum nicotianae* (SACC-1922) with MIC values of 0.64 and 0.69 mM; 1.37 and 1.44 mM; 1.80 and 1.91 mM and 1.55 and 1.71 mM, respectively. Nystatin was implemented as a positive control and showed MIC values of 0.007 and 0.006 mM. These bioactivities may be due to the presence of a formyl group at C-16 in the alkaloids subclasses picrinine in **42**, vincorine in **47**, and akuammiline in **51**, respectively and also may play a role in anti-inflammatory activity [41].

**Figure 9:**
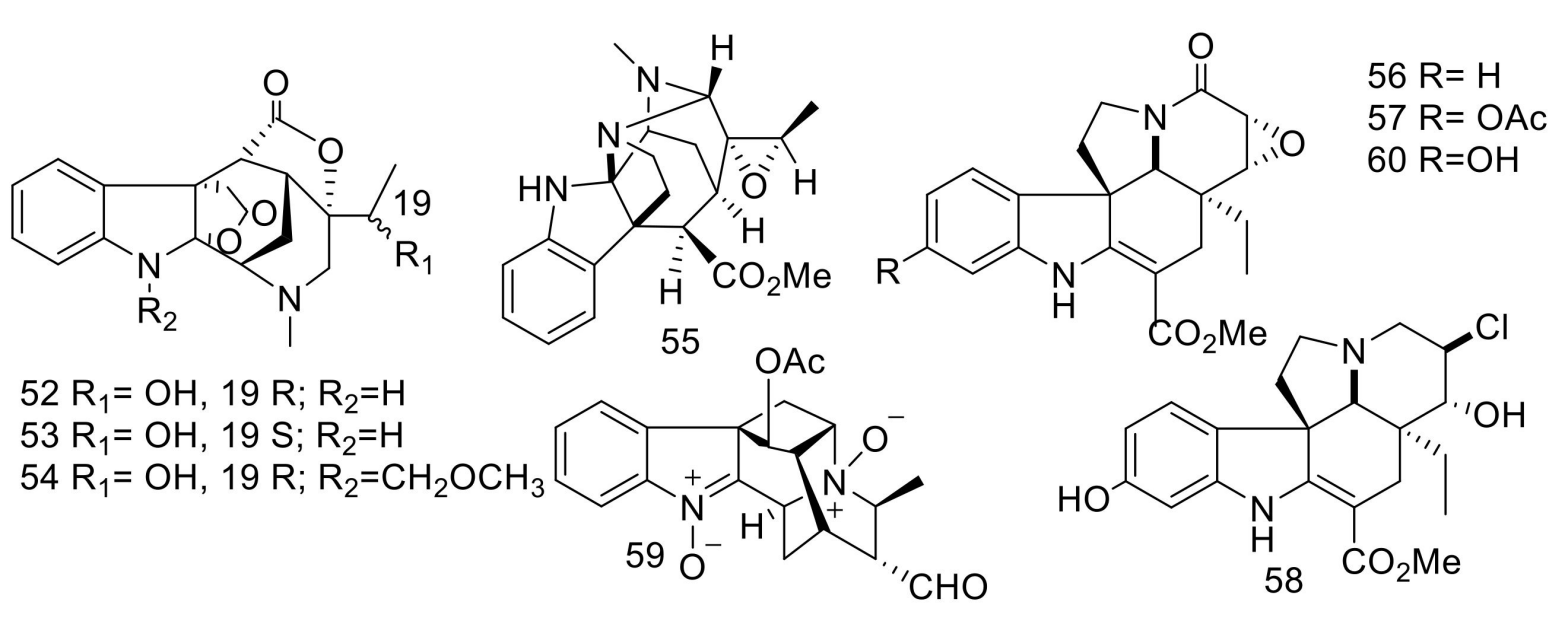
Compounds **52**–**60**.

Alstolactines A (**52**), B (**53**), and C (**54**) (Figure 9) were isolated from the leaves of chines *A. scholaris* [43]. The structures were identified by extensive spectroscopic data analyses and X-ray diffraction analyses. The absolute stereochemistry was deduced from crystal X-ray diffraction. These compounds are biosynthetically originated from picrinine, which is the main metabolite in *A. scholaris*. Compounds **52**–**54** exhibited no effects against four bacterial strains: *Klebsiella pneumonia, Escherichia coli*, *Pseudomonas aeruginosa and Staphylococcus aureus* [43].

Moreover, Alistonitrine A (**55**) (Figure 9) had an unprecedented caged carbon skeleton with a unique 6/5/6/5/5/6 ring system and also contained three nitrogen atoms. It was isolated from the same species [12]. Its structure and absolute configuration were established by extensive spectroscopic analyses and electron circular dichroism calculations. Compound **55** exhibited no activity as an anti-inflammatory in both NF-κB and HIF-α models [12].

The MIAs, 6,7-epoxy-8-oxo-vincadifformine (**56**), 11-acetyl-6,7-epoxy-8-oxo-vincadifformine (**57**), 11-hydroxy-14-chloro-15-hydroxyvincadifformine (**58**) and perakine *N*_1_,*N*_4_-dioxide (**59**) were identified from the aerial parts of *A. rupestris.* Additionally*,* 11-hydroxy-6,7-epoxy-8-oxovincadifformine (**60**) and **35** were isolated from the same species [44]. 

Compounds **56, 57** and **60** exhibited potent cytotoxic effects against head and neck squamous cancer (SCL-1, Detroit-562, UMSCC-1, CAL-27, TCA-83, HepG2 and SCC-PKU) cells, with IC_50_ values < 20 μM. Doxorubicin was implemented as a positive control and showed cytotoxicity, with IC_50_ values ≤ 35.4 µM. Compound **56** exhibited potent effect, with IC_50_ values ≤ 13.7 μM. This may be due to the absence of any substitution at the phenolic ring. This can be explained by the fact that the attachment of electron-donating groups (OH and OAc) led to a reduction in the cytotoxicity [44]. Compounds **56**, **57**, and **60** displayed significant antifungal activities against *Alternaria alternata* and *Phytophthora capsici*, with MIC values = 0.66 & 0.99 mM, 0.87 & 1.10 mM and 1.53 & 1.64 mM, respectively. Nystatin was implemented as positive control and showed effect with MIC values 0.007 and 0.061 mM. Compounds **56**, **57**, and **60** displayed moderate activity against *Staphylococcus aureus*, with MIC values of 15.72, 16.33 and 14.91 mM. Meanwhile, compounds **59** and **35** exhibited potent effects against *Staphylococcus aureus*, with MIC values of 0.49 and 0.83 mM. Rifampicin was used as a positive control and showed an effect at MIC valued = 0.003 mM for bacteria. Additionally, compound **59** showed higher antibacterial effects toward *S. aureus* than compound **35**. The present of a formyl group at the C-20 position might increase the activities for ajmaline indole alkaloids [44].

**Figure 10:**
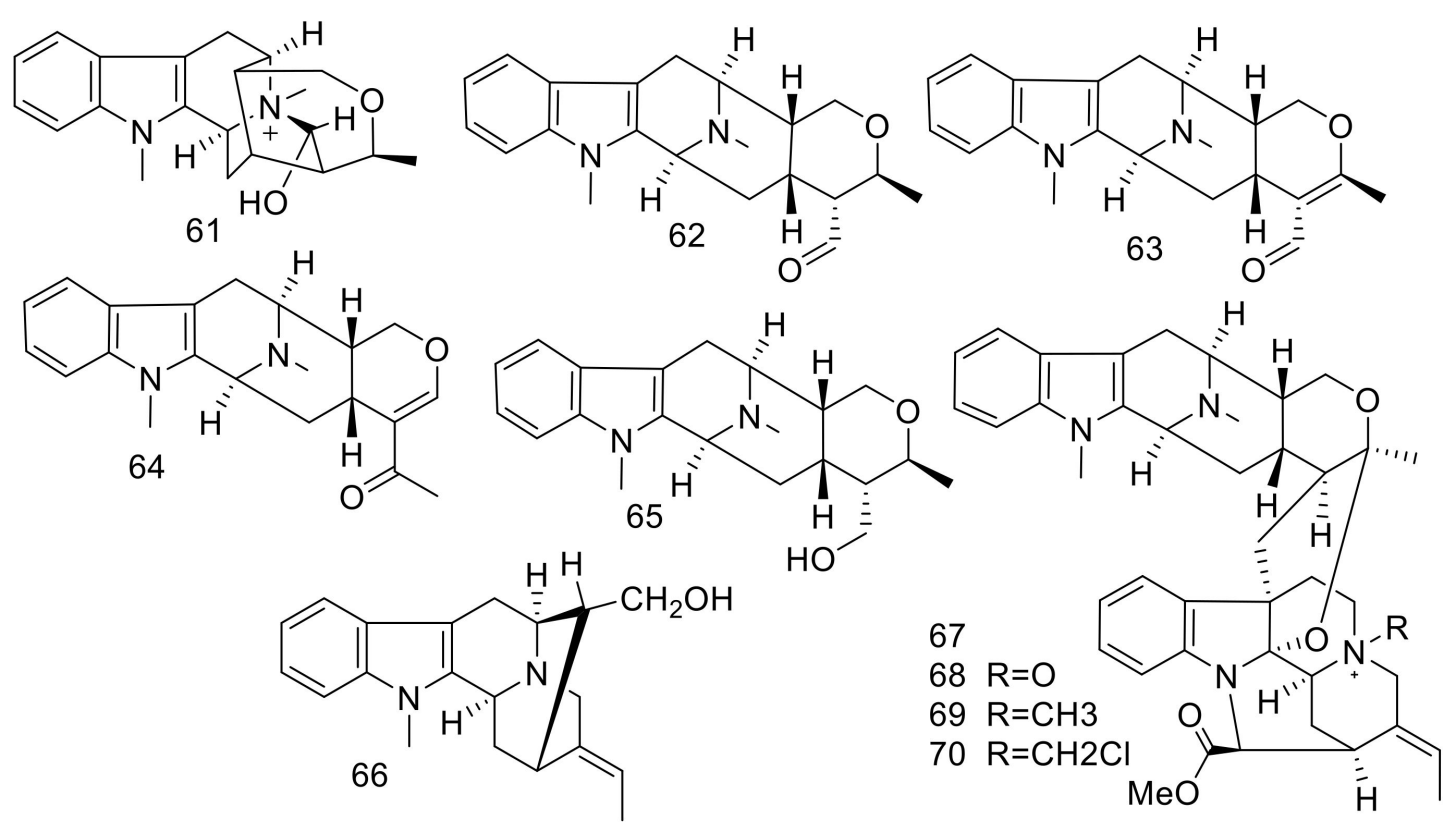
Compounds **61**–**70**.

The bioassay-guided fractionation of the stem bark of Vietnamese *Alstonia angustifolia* using the HT-29 human colon cancer cells, led to the reporting of six MIAs, *N*_(4)_-methyl-talpinine (**61**) [45], *N*_(4)_-meth-yl-*N*_(4)_,21-*seco*talpinine (**62**) [46], alstonerinal (**63**) [47], alstonerine (**64**) [48], macrocarpine B (**65**) [46], affinisine (**66**) [49], from the stem bark of *A. angustifolia.* Additionally, villalstonine (**67**), villalstonine *N*_(4)_-oxide (**68**) [50], villalstonidine D (**69**) and villalstonidine E (**70**) [51] (Figure 10) were identified from the same plant. 

Compounds **61** and **66** are sarpagine-type and compounds **62**–**65** are macroline-derived alkaloids whereas macroline-pleiocarpamine bisindole alkaloids are present in compounds **67**–**70**. 

Compound **61** showed significant inhibitory activity toward NF-κB (p65), with an ED_50_ value = 1.2 μM. Rocaglamide was employed as a positive control, with ED_50_ value = 0.9 μM. Compounds **61**–**64**, **66** and **68**–**70** showed anti-leishmanial activity toward the promastigotes of *Leishmania Mexicana*, with IC_50_ values < 183.5 μM. Compound **62** exhibited a potent effect, with IC_50_ value = 57.8 μM. Amphotericin B was employed as a positive control and exhibited potent effect against *L. mexicana* promastigote, with an IC_50_ value = 0.09 μM. The dimeric compounds **68**–**70,** which contain quaternary ammonium cation at *N*(4), exhibited potent effect than compound **67.** Additionally, compound **67** has not function group at *N*_(4)_ [45]. Also, the presence of formyl and acetyl groups in **62**–**64.** These moieties may enhance the effects of compounds belonging to macroline indole alkaloids compared with **65**. 

**Figure 11:**
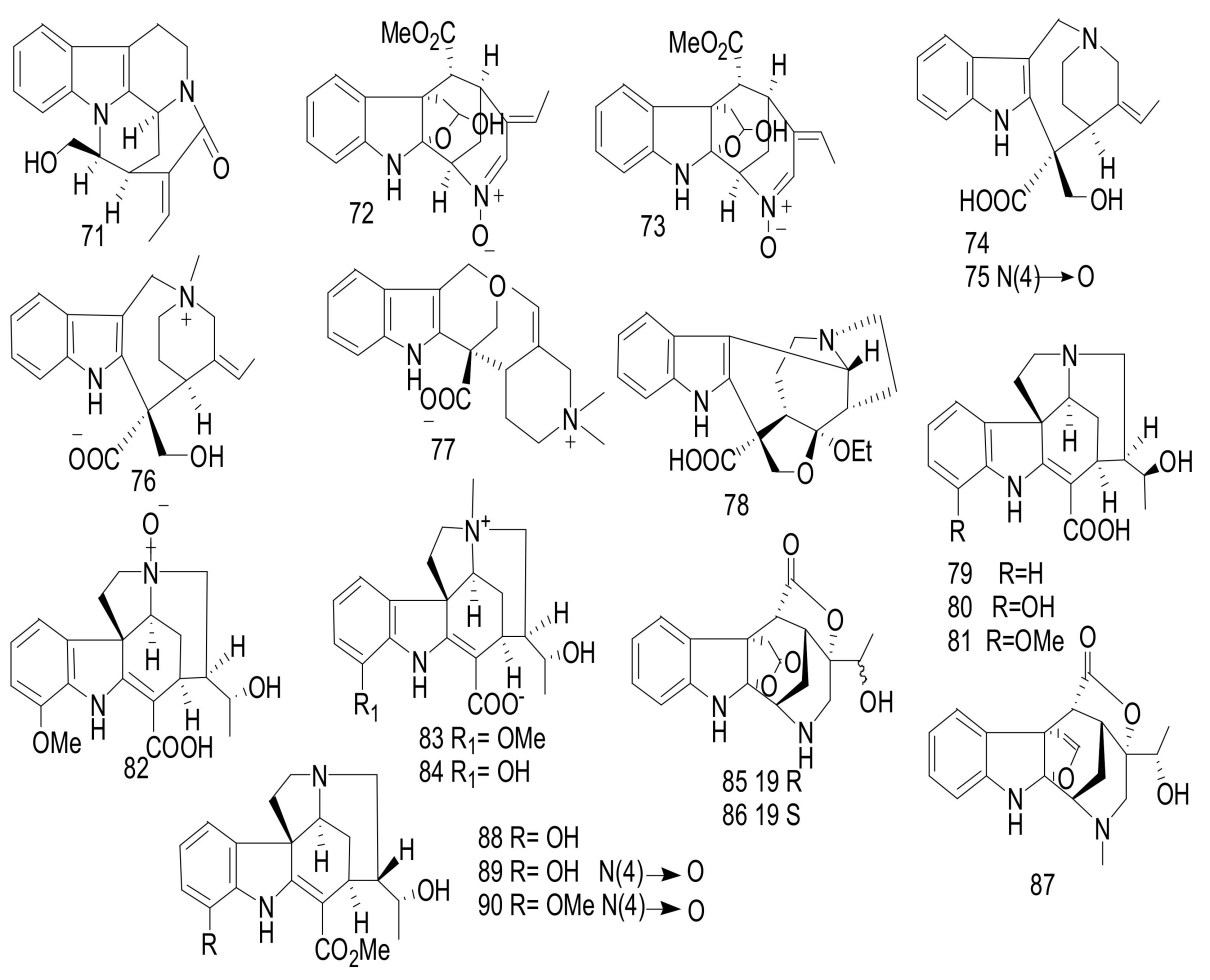
Compounds **71**–**90**.

Normavacurine-21-one (**71**), 5-hydroxy-19, 20-*E*-alschomine (**72**), and 5-hydroxy-19, 20-*Z*-alschomine (**73**) (Figure 11), were isolated from the leaves of *Alstonia scholaris* cultivated in Kunming, China [52]. Compound **71** exhibited a significant antimicrobial effect against *Enterococcus faecalis* ATCC 10541, with an MIC = 0.78 μg/mL, whereas compound **73** showed a significant effect against *Pseudomonas aeruginosa* ATCC 27853, with an MIC value = 0.781 μg/mL. Cefotaxime was used as a positive control, with an MIC = 0.19 μg/mL [52]. Alstoniascholarines A-Q (**74**–**90**), were identified from the leaves of *A. scholaris* collected from Yunnan [53,54]. Compounds **79** and **83** showed a potent antibacterial activity against *Pseudomonas aeruginosa* ATCC 27853, with MIC value = 3.13 mg/mL. Gentamycin was applied as a Positive control and showed an inhibitory effect, with an MIC value = 0.78 mg/mL. Additionally, compounds **77**, **80**, and **83** exhibited moderate antifungal activities toward *Epidermophyton floccosum* CBS 566.94, with MIC value s= 31.25 mg/mL. Griseofulvin was applied as a positive control and showed an inhibitory effect, with an MIC value = 7.81 mg/mL [53]. Compounds **85**–**90** showed no cytotoxicity against five tumor cell: MCF-7, A-549, HL-60, SW-480, and SMMC-7721[54].

**Figure 12:**
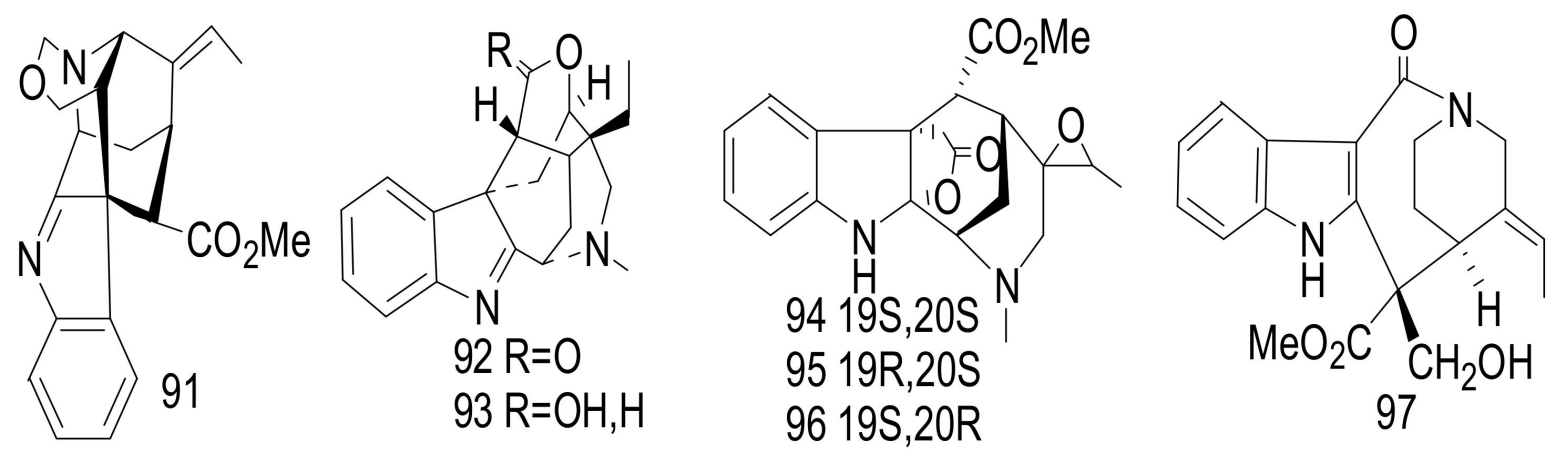
Compounds **91**–**97**.

Scholarisines H-O (**91**–**97**) (Figure 12) were isolated from the leaves of the Chinese *A. scholaris* [55]. The chemical structures were elucidated on the basis of comprehensive spectroscopic data and X-ray diffraction. Compounds **91**–**97** showed weak antibacterial activities against five strains: *Pseudomonas aeruginosa* ATCC 27853, *Staphylococcus aureus* ATCC 25922, *Escherichia coli* ATCC 11775, *Providencia smartii* ATCC 29916, and *Enterococcus faecalis* ATCC 10541), with MIC values = 100 μg/mL. Gentamycin was used as a positive control, with an MIC value < 2.00 μg/mL [55]. 

**Figure 13:**
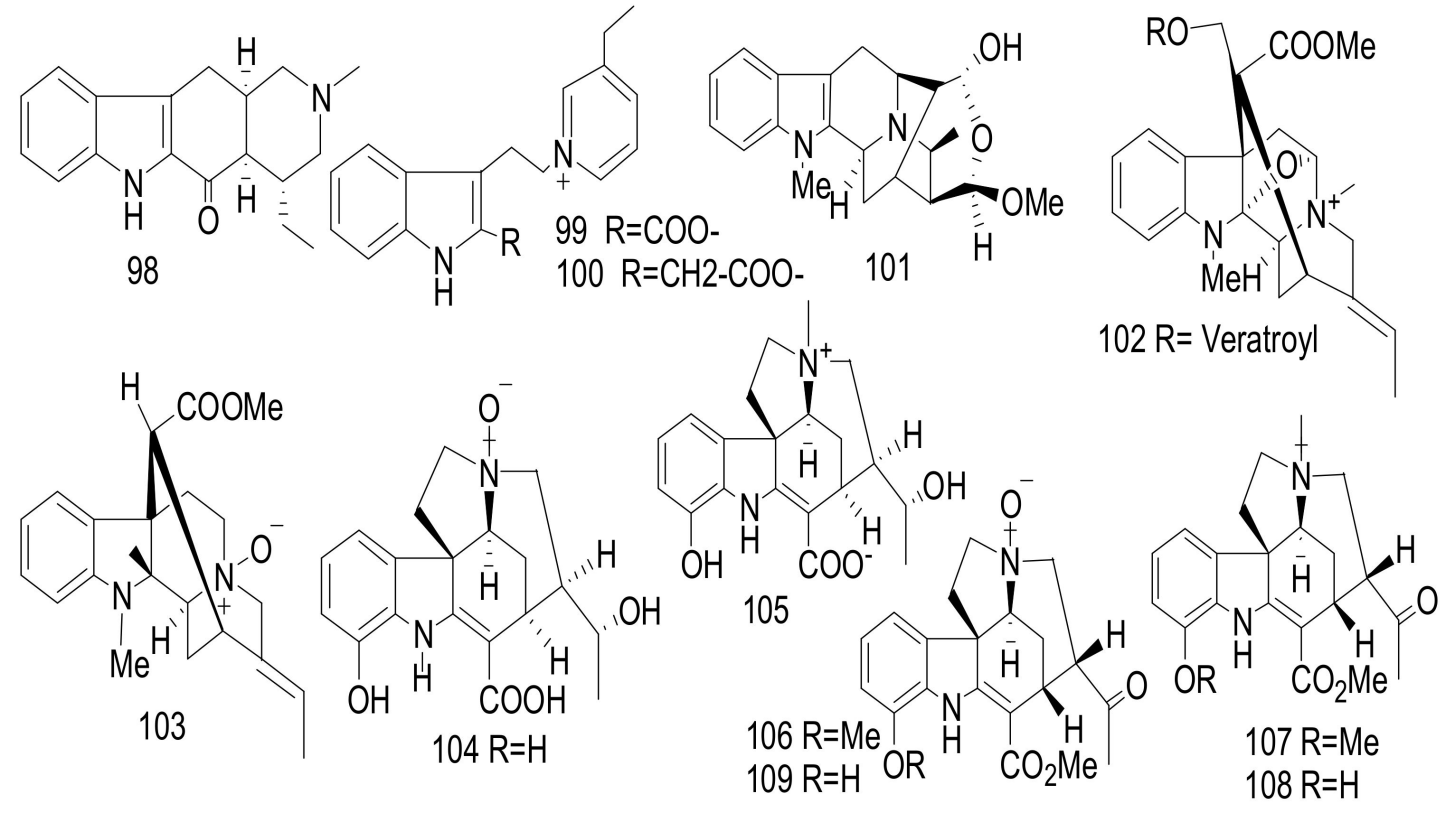
Compounds **98**–**109**.

A further study on the leaves and twigs of *A. scholaris* [56] led to identification of melosline A (**98**), B (**99**) and 1-[2-[2-(carboxymethyl) indole-3-yl] ethyl]-3-ethylpyridinium hydroxide inner salt (**100**) (Figure 13) [57]. Melosline A (**98**) was an unprecedented indole alkaloid, with a 6/5/6/6 tetracyclic ring skeleton. The structures were established by spectroscopic analyses. The absolute configuration of **98** was confirmed by the comparison of experimental data with the calculated electronic circular dichroism (ECD). Compound **98** showed a moderate cytotoxic activity against breast cancer (MCF-7), with an IC_50_ value = 39.78 μM. Cisplation was employed as a positive control [56].

Alstiyunnanenines A-E (**101**–**105**) (Figure 13) and alstoniascholarine I (**82**) (Figure 11), were isolated from *A. yunnanensis* [54,58]. Compounds **104**, **105,** and **82** displayed potent cytotoxicity against human gastric carcinoma (BGC-823 cells), human hepatocellular, (HepG2 cells), human myeloid leukemia (HL-60), human breast cancer (MCF-7), and osteosarcoma (SOSP-9607, MG-63, Saos-2, M663), with IC_50_ values ranging between 3.2 and 5.8 μM. Adriamycin was used as a positive control and exhibited cytotoxicity, with an IC_50_ value < 0.04 μM [58]. Three monoterpenoid indoles, alstomairines A-C (**106**–**108**) [59], together with alpneumine A (**109**) [60] were identified from the leaves of the chines *A. mairei*. Compounds **107** and **108** showed potent cytotoxic effects against osteosarcoma cells (U2-OS, Mg-63, Saos-2, and SOSP-9607) with IC_50_ values ranging from 9.2 to 13.0 μM, whereas compounds **106** and **109** had IC_50_ values < 15.0 μM. The presence of the methyl group on *N*-_4_ indicate increasing the cytotoxicity in that scholaricine-type (Figure 4) than the presence of *N*_(4)_-oxide moiety. Doxorubicin was used as Positive control and showed cytotoxicity, with an IC_50_ value < 0.03 μM [59].

**Figure 14:**
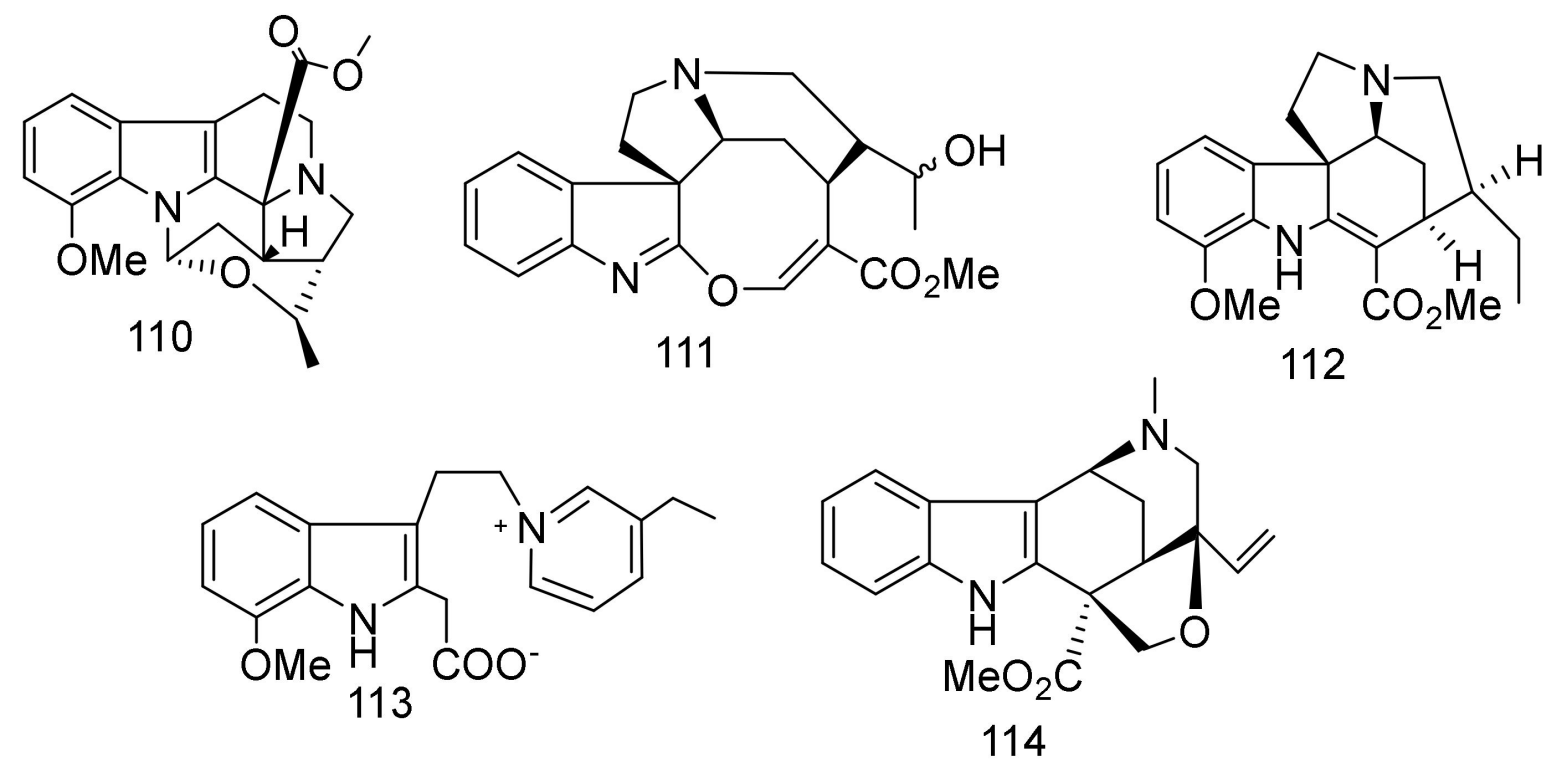
Compounds **110**–**114**.

Alstrostine G-K (**110**–**114**) (Figure 14), were identified from the Chinese *A. rostrata* [61]. Compounds **110**–**114** showed no cytotoxicity against HeLa, SGC-7901 gastric cancer, and A-549 lung cancer at 20 µM [61].

**Figure 15:**
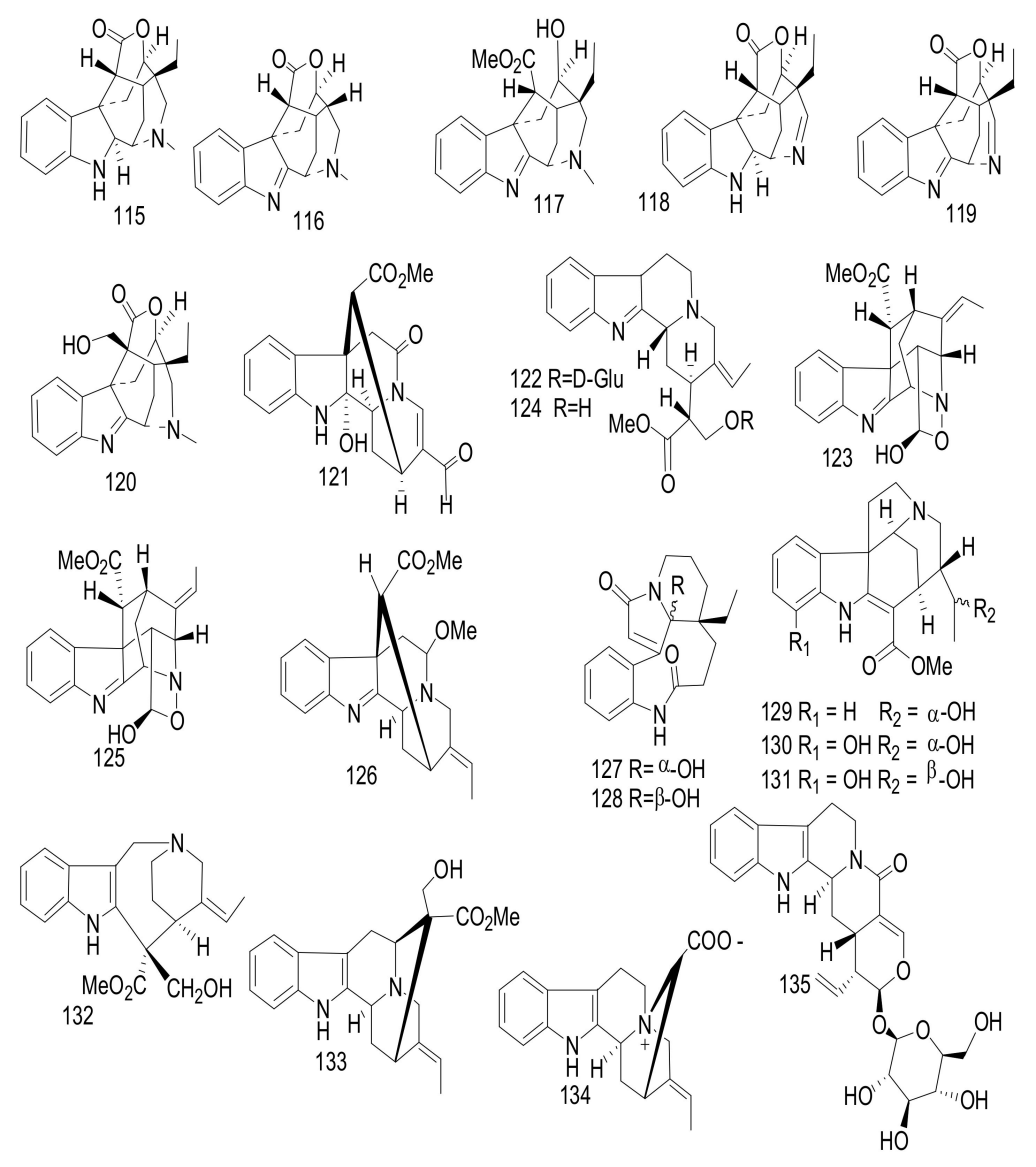
Compounds **115**–**135**.

Six nareline-type indoles including three cage-like skeletons, scholarisines T-V (**115**–**117**) [62] (Figure 15), and three previously identified analogues scholarisine W (**118**), scholarisine A (**119**), and scholarisine I (**92**) [55], were isolated from the leaves of the Chinese *A. scholaris* [56]. Compounds **115**–**117** displayed anti-bacterial effects against *Escherichia coli* ATCC 8739, with an MIC value = 0.78 μg/mL. Additionally, compound (**116**) inhibited the growth of *Bacillus subtilis* ATCC 6633 bacterium with an MIC value = 3.12 μg/mL and was referenced with cefotaxime as a positive control. The absence of the ethyl group at C-20 position indicated an increase in the anti-bacterial activities as in **116**, compared with compounds (**115** and **117**) [63]. Cefotaxime was used as a positive control and exhibited an inhibitory effect, with an MIC value of 0.39 μg/mL. There were scholarisines P-S (**120**–**123**), (16*R*)-*E*-isositsnikine (**124**) [64], nareline (**125**) [65], 5-methoxystrictamine (**126**), leuconolam (**127**), epileuconolam (**128**) [66], and *N*^b^-demethylalstogustine (**129**) [67]. Also, 19-epischolaricine (**130**), scholaricine (**131**), vallesamine (**132**) [68], akuammidine (**133**) [69], 17-*nor*-excelsinidine (**134**) [70], strictosamide (**135**) [71,72] and compounds **19** and **21**, were isolated from the same species. Compounds **123**, **19**, **21**, **130** and **133** exhibited significant NF-κB inhibitory activity with IC_50_ < 25 μM. Furthermore, compounds **19**, **126** and **130** inhibited TNFα-induced NF-κB activation in the same dose. Three nareline-type MIAs, compounds (**120**, **123** and **125**) were identified from *A. scholaris* [73].

**Figure 16:**
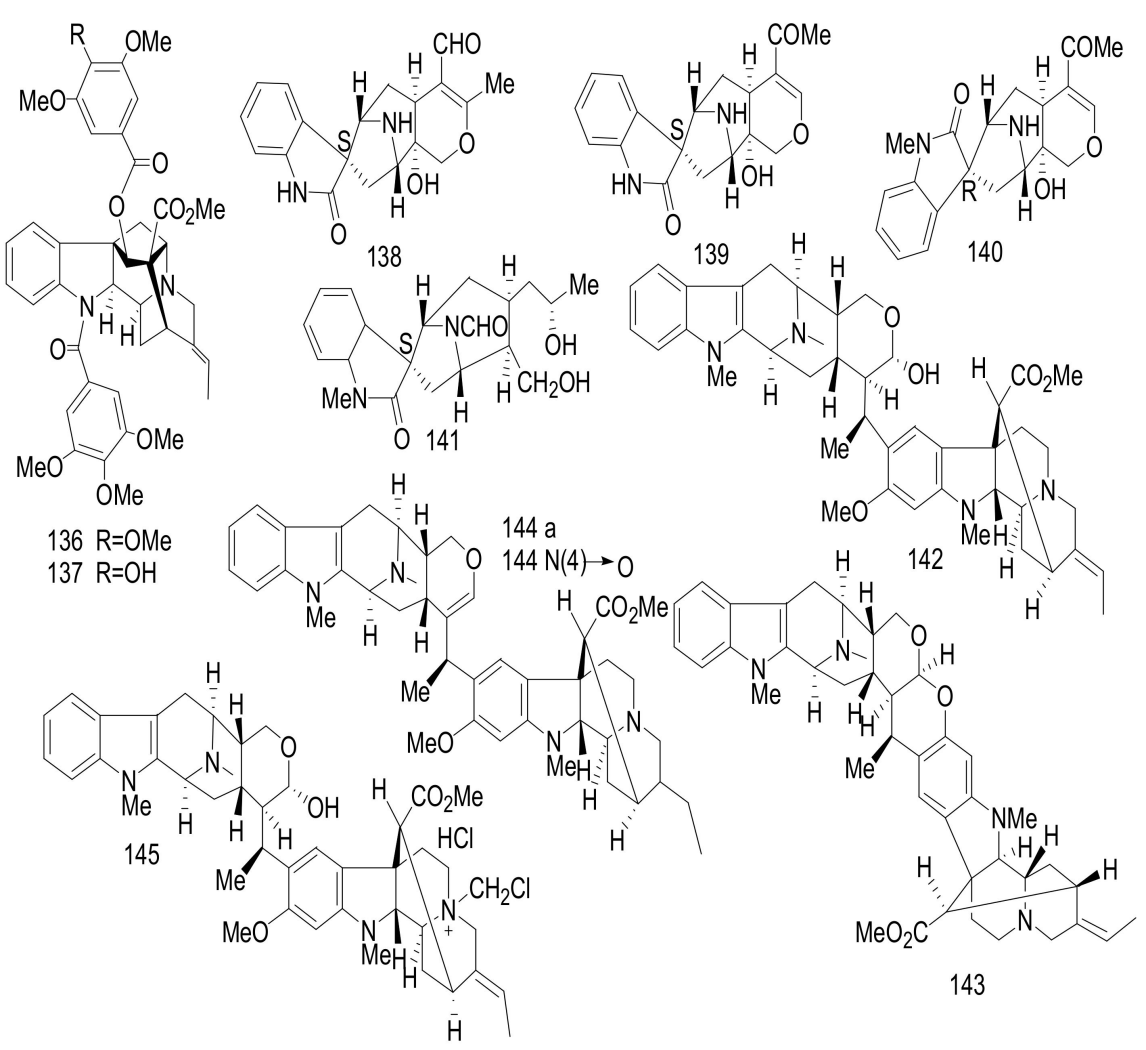
Compounds **136**–**145**.

Two ajmaline type MIAs, vincamaginine A (**136**), and vincamaginine B (**137**); four macroline oxindole- alstonisinines A (**138**) and B (**139**), alstonisinine C (**140**), and alstonoxine F (**141**); four bisindole compounds of macroline-akuammiline type; angustilongine A–D (**142**–**145**) (Figure 16) were reported from Malaysian *Alstonia penangiana* [73]. The structures of these alkaloids were determined by the interpretation of spectroscopic data and compounds **141**–**142**, were confirmed by X-ray diffraction analysis. Compounds **142** and **143** showed growth inhibitory activity against human prostate carcinoma (LNCaP and PC-3), human breast adenocarcinoma (MDA-MB-231 and MCF7), human colorectal carcinoma (HCT 116 and HT-29) and human lung carcinoma (A549). Furthermore, the potent effects of **142** and **143** against HT-29 cells were evaluated, with IC_50_ values = 0.7 ± 0.1 μM and 0.3 ± 0.0 μM, respectively (Cisplatin, IC_50_ >10 μM). Compound **143** exhibited an effect against vincristine-resistant KB cells, with an IC_50_ value of 0.7 ± 0.3 μM (Vincristine 0.3 ± 0.1 μM) [73]. 

**Figure 17:**
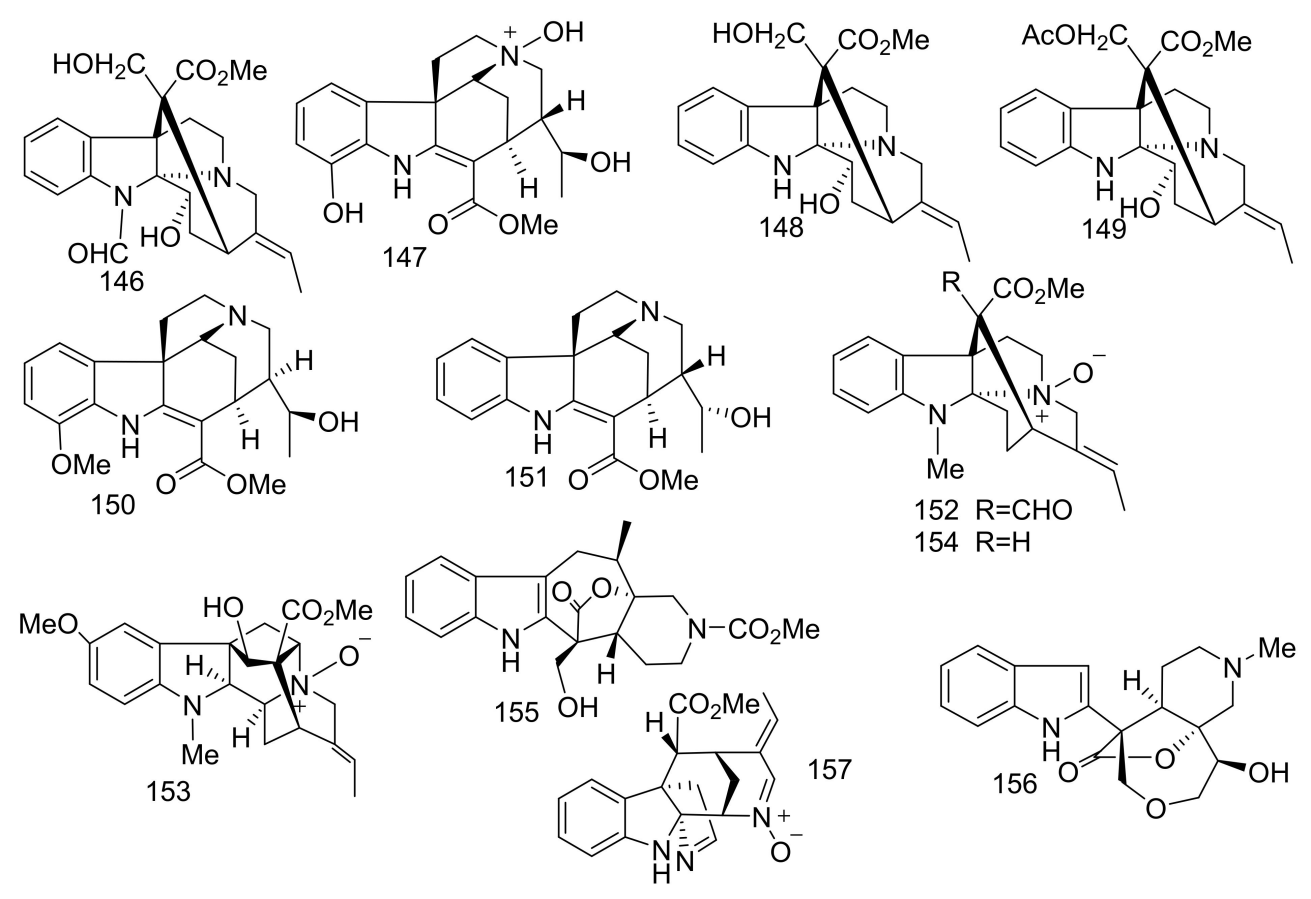
Compounds **146**–**157**.

Winphyllines A (**146**), B (**147**) [74], *N*_b_-demethylechitamine (**148**) [75], 17-*O*-acetylnorechitamine (**149**) (Figure 17) [76], 12-methoxyechitamidine (**150**) [67], and *N*_(4)_-demethylastogustine (**151**) [77] were isolated from the collected twigs of the Chinese *A. rostrata*. Compounds **146**–**151** exhibited cytotoxicity against cancer cells (HL-60, SMMC-7721, A-549, MCF-7, and SW480), with IC_50_ values = 40 μM [74]. A vincorine-type, 17-formyl-10-demethoxyvincorine *N*_(4)_-oxide (**152**), an ajmaline-type 10-methoxyalstiphyllanine H (**153**), and 10-demethoxyvincorine *N*_(4)_-oxide (**154**) were obtained from the leaves of *A. scholaris* [78]. The phytochemical investigation of *A. scholaris* led to the publication of alstoscholactine (**155**) and alstolaxepine (**156**) [79]. A further investigation on the leaves of Malaysian *A. scholaris* led to the reporting of alstobrogaline (**157**) [80]. Compounds **155** and **156** exhibited no cytotoxic effects, whereas **156** induced marked vasorelaxation in reported rat aortic rings precontracted with phenylephrine, with EC_50_ = 6.58 ± 3.66 μM and Emax = 93.9 ± 4.3% (cf. verapamil, EC_50_ = 0.55 ±0.19 μM and Emax = 106.4 ± 3.4%) [74]. Compound **157** showed weak cytotoxic activity against breast cancer cells MDA-MB-231 and MCF7, with IC_50_ values = 25.3 and 24.1 μM, respectively [80].

The scaffold of the reported monoterpene indole compounds from *A. scholaris* is affected by the geographical environment. Indian, Pakistanian and Thai. *A. scholaris* are rich with picrinine-type indole compounds, whereas, those identified from Indonesia and the Philippine*,* are rich in angustilobine-type [81]. Genus *Alstonia* was addressed as a source of angustilongines A (**142**) and B (**143**). These compounds showed more potent anticancer activities than those recognized from *A. penangiana,* although all of them belong to macroline- and akuammiline-type bisindole alkaloids. 

A review entitled "*Alstonia scholaris* and *Alstonia macrophylla*: A comparative review on traditional uses, phytochemistry and pharmacology" was published in 2014 and mentioned the compounds obtained from A. *scholaris* from 1976 to 2009, and from *A. macrophylla* from 1987 to 2013 [82]. A review published in 2018 entitled "The alstoscholarisine compounds: isolation, structure determination, biogenesis, biological evaluation and synthesis" studied the alstoscholarisine compounds obtained from *A. scholaris* [83]. Furthermore, a review published in 2016 called "An overview phytochemistry and chromatographic analysis of *Alstonia scholaris* used as a traditional medicine" discussed *A*. *scholaris* compounds which were reported between 1965 and 2009 [84].

The identified metabolites from *Alstonia* were categorized under two main classes: corynanthe and aspidosprma. Corynanthe contains eight subclasses: ajmaline-type (**18,** and **33**–**35**), picrinine-type (**19**–**21** and **42**–**44**), akummiline-type (**22**), vincorine-type (**47**, **50** and **148**–**149**), sarpagine-type (**61**, **66** and **101**), macroline-type (**62**–**65**), scholaricine-type (**104**–**109**) and macroline oxindole-type (**138**–**141**). Meanwhile,, aspidosprmia contains six subclasses: aspidosprma-type (**31** and **32**) vincamine-type (**82**–**84** and **88**–**90**), aspidofractinine-type (**1** and **4**), bisindole alkaloids macroline-pleiocarpamine-type (**67**–**70**), and macroline- akuammiline-type (**142**–**145**). Ajmaline derivatives with formyl group and/or a quaternary ammonium cation *N*(4) showed an interesting bioactivity. 

## 3. Kopsia 

*Kopsia* (Family Apocynaceae) contained 30 species with a distribution in China, India, Southeast Asia, and Australia. Sixteen species were grown in Malaysia [85], and five species were grown in Thailand [86]. These plants are considered as rich sources of indole-containing compounds. Traditionally, some of the species have been used for the treatment of tonsillitis, dropsy and rheumatoid arthritis. Several species have been reported to have antitumor, antimanic, antitussive and antileishmanial effects [87,88,89]. A review published in 2017 was interested in reporting indole alkaloids from genus *kopsia* plants regarding reversing multidrug resistance in vincristine-resistant KB cells for example, kopsirensine B, arboloscine A [90], grandilodines A and C, and lapidilectine B [91,92].

**Figure 18:**
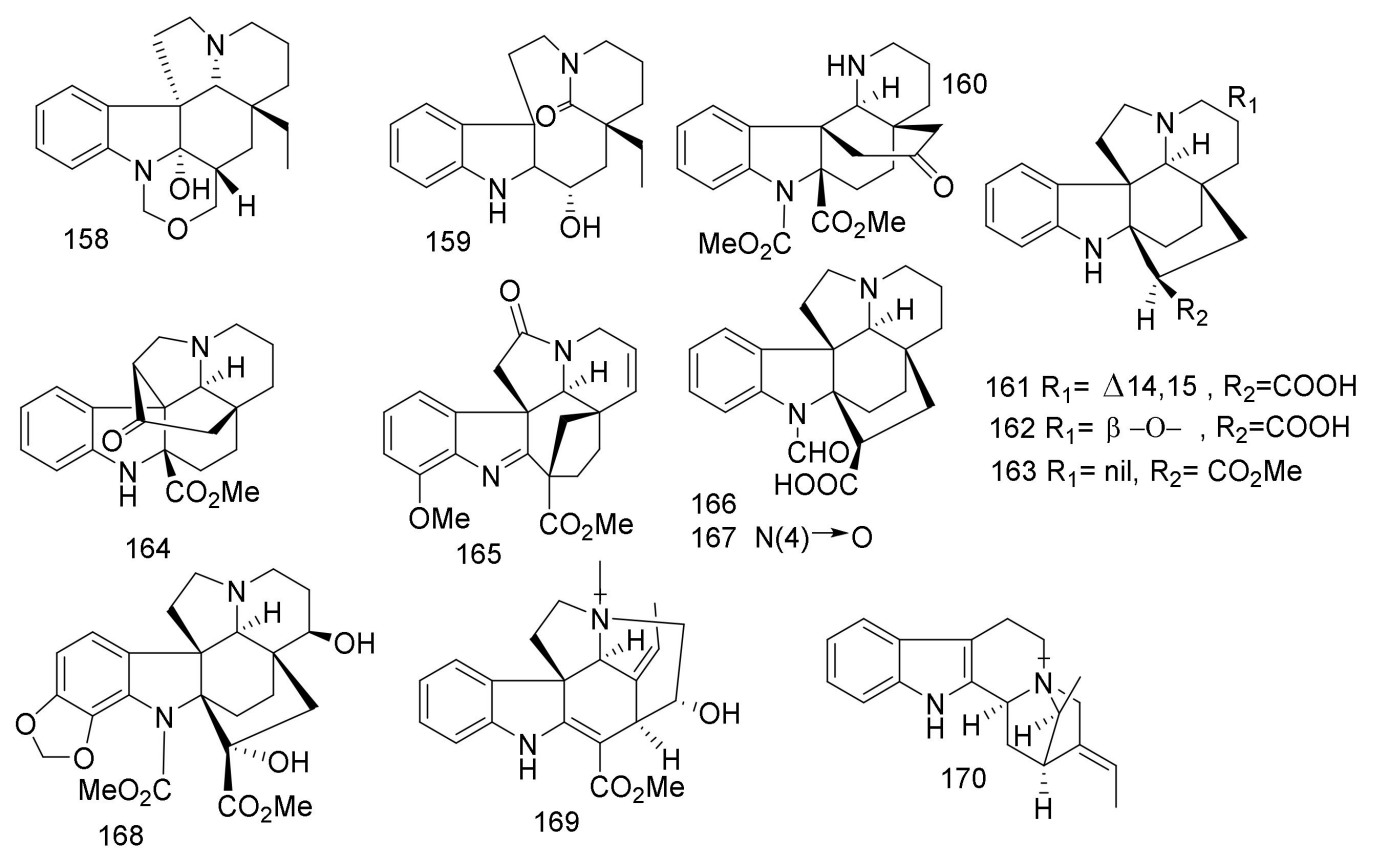
Compounds **158**–**170**.

Kopsiyunnanines G (**158**) and kopsiyunnanines H (**159**) (Figure 18) with an aspidosperma-containing skeleton were isolated from the aerial part of the Chinese *Kopsia arborea* [93]. Kopsihainins A (**160**), B (**161**), and C (**162**) were isolated as new compounds from *K. hainanensis* [89], along with the known compounds, kopsinine (**163**) [87] and methyl demethoxycarbonylchanofruticosinate (**164**) [94] were isolated from the stems of Chinese *K. hainanensis*. Compounds **163** and **164** showed significant antitussive activity, these compounds are within the aspidofractinine-type and methyl chanofruticosinate-type indoles, respectively. Compounds **163** and **164** inhibited coughing by 88% and 76%, respectively [83]. Compound **163** was more active, with an ID_50_ value = 0.11 mmoL/kg, whereas compound **164** exhibited an effect, with an ID_50_ value = 0.45 mmol/kg, (Codeine, ID_50_ = 0.1 mmol/kg) [90]. The link from C-2 to C-20 in compound **163** and the attachment of the methoxy carbonyl group at C-16 position promote the antitussive activity. 

Four alkaloids of aspidofractinine-type, singaporentine A (**165**), *N*_(1)_-formylkopsininic acid (**166**), *N*_(1)_-formylkopsininic acid-*N*_(4)_-oxide (**167**), and 15-hydroxykopsamine (**168**), along with an aspidospermatan-type, 14α-hydroxy-*N*_(4)_-methylcondylocarpine (**169**), and singaporentinidine (**170**) (Figure 18) were identified from the barks and leaves of Malaysian *K. singapurensis* [95].

**Figure 19:**
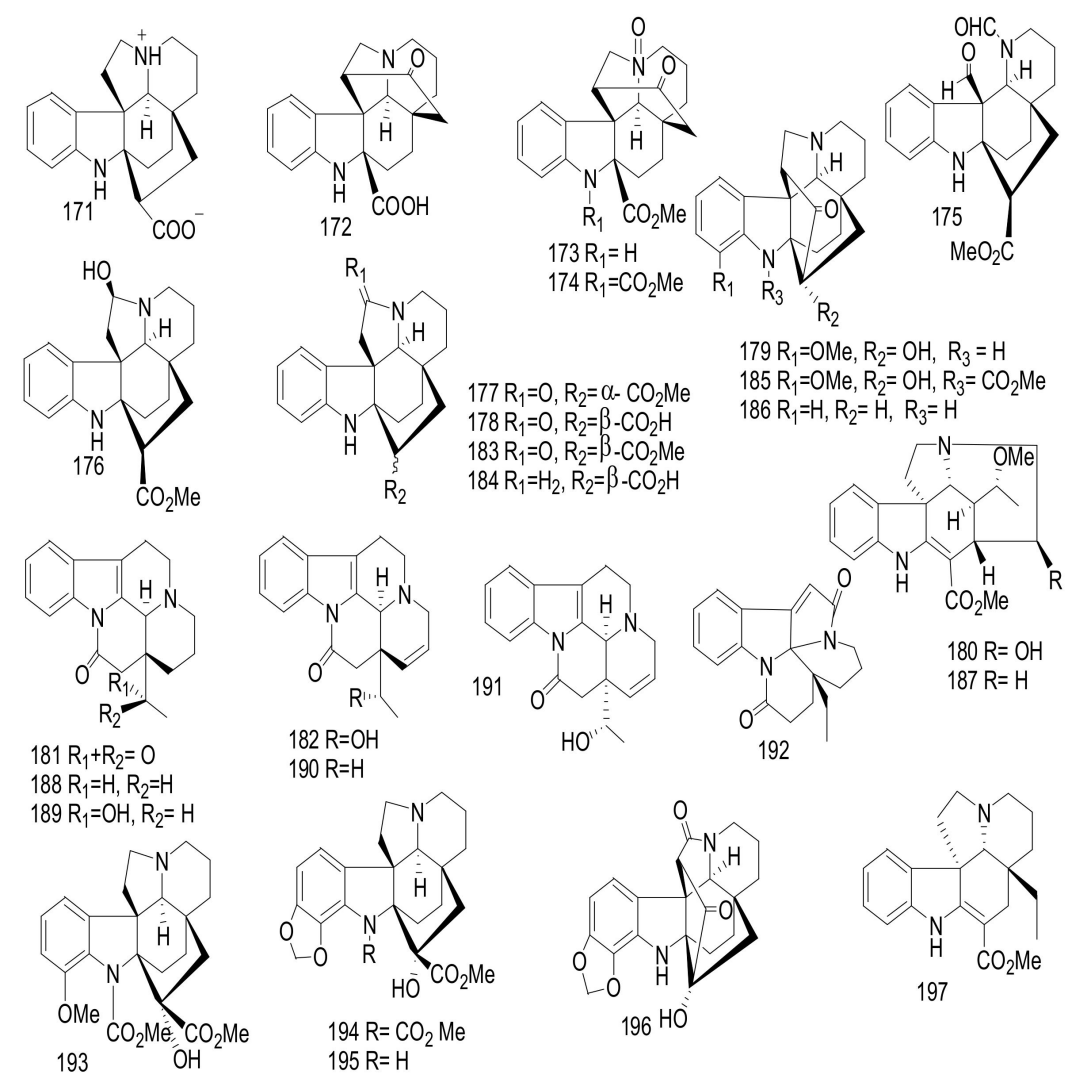
Compounds **171**–**197**.

From the leaves and stems of the Chinese medicinal plant *K. hainanensis*, four compounds, kopsininate (**171**), *N*_1_-decarbomethoxy chanofruticosinic acid (**172**), methyl *N_1_*- decarbomethoxy chanofruticosinate *N*_(4)_-oxide (**173**) and methyl chanofruticosinate *N*_(4)_-oxide (**174**) (Figure 19) were reported [96]. Compound **172** was the most effective against *Erwinia carotovora* bacterium, with an MIC of 7.8 mg/mL. Furthermore, compound **172** showed antifungal activities against four plant pathogenic fungi: *Penicillium italicum, Fusarium oxysporum* f. sp. Niveum, *Rhizoctonia solani* and *Fusarium oxysporum*. Cubense had an EC_50_ = from 15.2 to 43.8 μg/mL dose values. Compound **172** showed a potent effect towards *F. oxysporum* f. sp. Cubense, with an EC_50_ = 15.2 mg/mL. A comparison of this result with the positive control Midlothian, with an EC_50_ = 57.0 mg/mL showed compound **172** to be more active. The presence of carboxylic group attached to the C-2 position in **172** is important for antifungal activity, particularly, in methyl chanofruticosinate-type indoles [96]. 

Three aspidofractinie-type compounds, 5,6-secokopsinine (**175**), 5β-hydroxykopsinine (**176**), 16-*epi*-kopsinilam (**177**) [97], two kopsine-type metabolites, 5-oxokopsinic acid (**178**), and *N*_a_-demethoxycarbonyl-12-methoxykopsine (**179**) [97], a strychnos-type, 14(*S*)-hydroxy-19(R)- methoxytubotaiwine (**180**), and vincamine-type, and strychnos type 19-oxo-(−)-eburnamonine (**181**), 19(*S*)-hydroxy-Δ^14^-vincamone (**182**) [97], along with ten known compounds, **163** [87], kopsinilam (**183**) [98], kopsinic acid (**184**), 12-methoxykopsine (**185**) [99], kopsanone **186**), 19(R)- methoxytubotaiwine (**187**) [88], (−)-eburnamonine (**188**), 19-OH-(−)-eburnamonine (**189**), and Δ^14^-vincamone (**190**) [97] were yielded from the stem bark of the Thai *Kopsia jasminiflora* (Figure 19). Compounds **163**, **183**, and **184** belong to aspidofractinie-type, **185** and **186** belong to Kopsine-type, **187** belongs to strycno-type, **188**–**190** belonging to the vincamine- type MIAs.

The vincamine-type compound **182** showed a potent inhibitory activity against HT29, HCT116, and A549 cancer cells, with IC_50_ values = 0.36, 0.40, and 0.51 μM, respectively. Meanwhile, compounds **188** and **189** showed moderate activities with IC_50_ values ranging from 2.00 to 2.61 μM (Docetaxel, IC_50_ < 0.0005 μM). These results indicated the structural features that are necessary for the presence of a vincamine-type carbonyl group at the C-16 position, forming an amide function group, and a methylene group or hydroxyl methine at C-19 position in **182**, **188**, and **189** [97]. The presence of a double bond in the piperidine ring between C-15 and C-16 may be responsible for increasing the activity of compound **182**. 

A study on the content of twigs of *K. arborea* grown in Thailand revealed the isolation of a new MIA, phutdonginin (**191**) [100], an eburnane-type compound, together with eight known compounds, among them, **164 [87]**, **189** [88] melodinine E (**192**) [101], kopsilongine (**193**), kopsamine (**194**) [94], (−)-methylenedioxy-11,12-kopsinaline (**195**) [87], decarbomethoxykopsiline (**196**) [102], and vincadifformine (**197**) [103]. Only **194** and **196** displayed AChE inhibition activity with MIR values 12.5 and 6.25 μg, respectively, compared with reference drug galanthamine MIR = 0.004 μg. In addition, compounds **194** and 198 also displayed the weak acetylcholinesterase (AChE) inhibition of 23.3% and 45.7% in a microplate test at 1 mM. Compounds **191** and **189** showed moderate inhibition of bacterium toward Escherichia coli TISTR 780 with MIC = 32 μg/mL, with vancomycin and gentamycin references drugs with MIC values 0.125–0.25 μg/mL [100].

**Figure 20:**
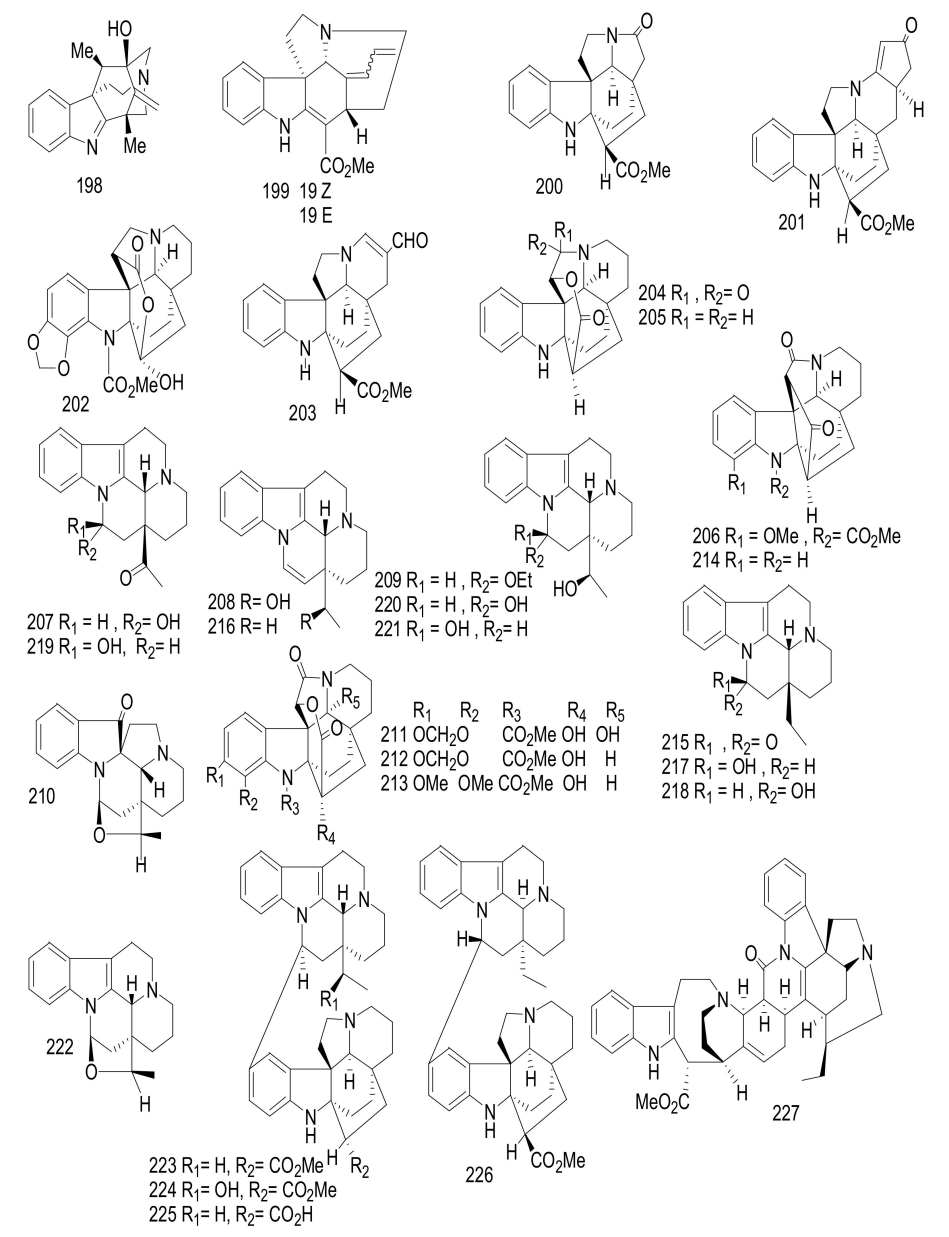
Compounds **198**–**227**.

Malaysian *Kopsia arborea* was investigated and arboridinine (**198**) [85] was reported (Figure 20). The further investigation of the aerial parts of *K. arborea* led to the isolation of kopsiyunnanines J1 and J2 (**199a** and **199b**) [104]. Compound **198** exhibited a moderate relaxation effect that was dependent on the contraction of phenylephrine-induced in the rat aortic rings, with an EC_50_ of 4.98 μM, and an E_max_ 60.6 ± 7.8% with the reference control isoprenaline with an EC_50_ value = 0.08 μM, and an E_max_ 79.7 ± 4.2% [85].

Seven aspidofractinine -type alkaloids, paucidirinine (**200**), paucidirisine (**201**), paucidactinine (**202**), pauciduridine (**203**), paucidactine D (**204**), paucidactine E (**205**), and paucidisine (**206**), along with Additionally, four eburnane skeleton, (−)-19-oxoisoeburnamine (**207**), (−)-19(*R*)-hydroxyeburnamenine (**208**), (−)-19(*R*)-hydroxy-*O*-ethylisoeburnamine (**209**), and larutienine B (**210**) were isolated from *Kopsia pauciflora* [91]. Moreover, twelve compounds, paucidactine A (**211**), paucidactine B (**212**) [105], paucidactine C (**213**) [88], 5, 22-dioxokopsane (**214**) [98], (+)-eburnamonine (**215**) [94], (+)-eburnamenine (**216**) [106], (−)-eburnamine (**217**), (+)-isoeburnamine (**218**) [94], (+)-19-oxoeburnamine (**219**) [105], (−)-19(*R*)-hydroxyisoeburnamine (**220**), (+)-19(*R*)-hydroxyeburnamine (**221**) [87], and larutienine A (**222**) [90] were published. Furthermore, three bisindole compounds have been identified, (−)-norpleiomutine (**223**), (+)-kopsoffinol (**224**) [107], and (−)-demethylnorpleiomutine (**225**) [87] and (+)-kopsoffine (**226**) (Figure 20) [107], were identified from the same species. A bisindole alkaloid were isolated by Kitajima at et from Yunnan *Kopsia arborea*, named Kopsiyunnanine M (**227**) [108]. 

Compounds **223** and **224** exhibited growth inhibitory activity against MCF-7, PC-3, A549, and HCT-116, with IC_50_ values ranging between 11.5 and 25.1 μM (Cisplatin, IC_50_ value in the range of 5.0–14.3 μM). The obliteration of the biological activity in **225** may be due to the presence of a carboxylic group in C-16, instead of a methoxycarbonyl group in **223** [91]. Arborisidine (**228**) and arbornamine (**229**) were isolated from a Malaysian *K. arborea*. Compound **228** represented a unique skeleton [109]. Compounds **228** and **229** exhibited no activities against KB, PC-3, HCT116, A549 and HT-29 cells [109]. 

**Figure 21:**
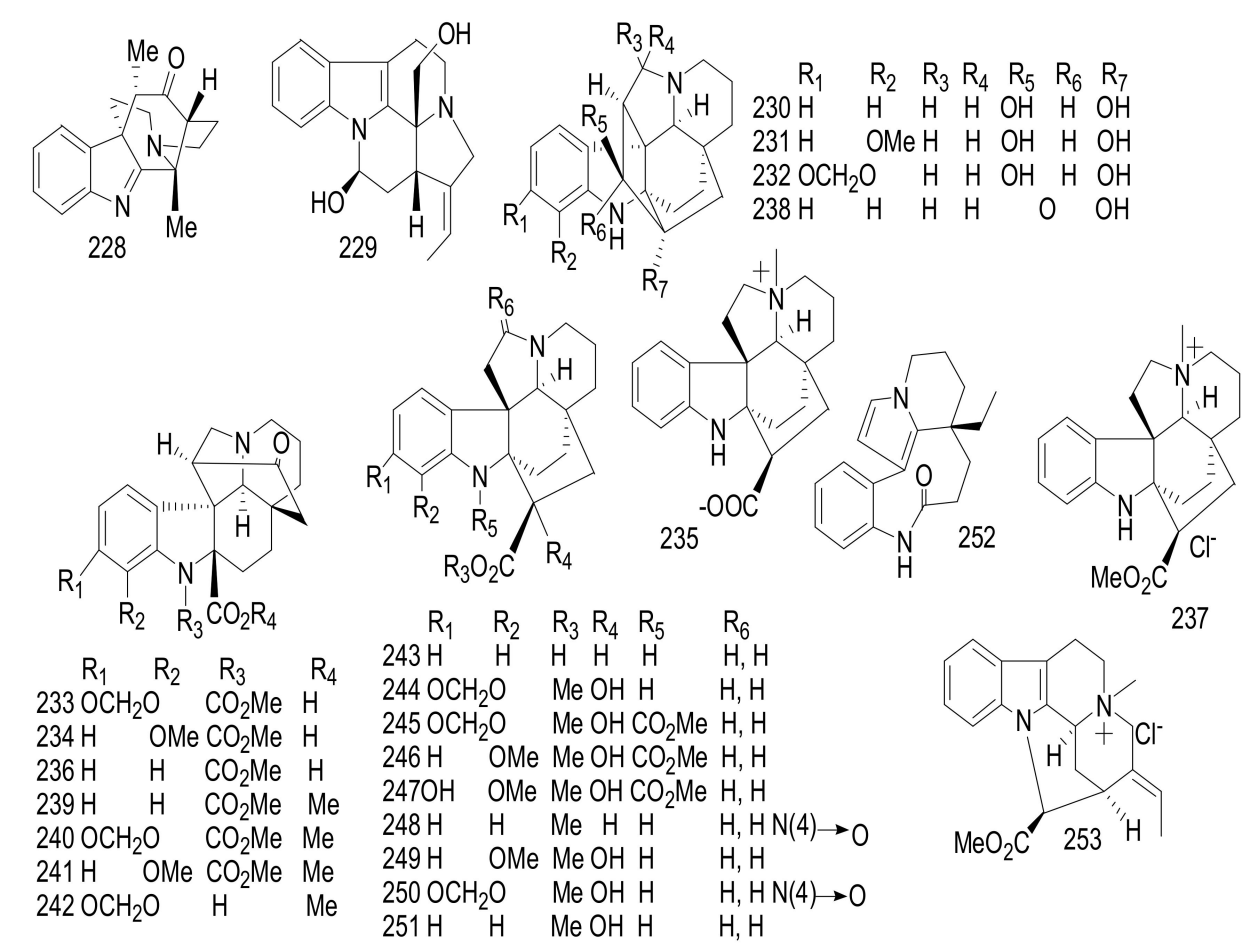
Compounds **228**–**253**.

Six new Kopsinidine C-E (**230**–**232**), 11,12-methylenedioxychanofruticosinic acid (**233**), 12-methoxychanofruticosinic acid (**234**), and N(4)-methylkopsininate (**235**), in addition to chanofruticosinic acid (**236**) as new natural compound [110], along with compounds **163**, **164**, **178**, **183**, **179**, and **215** (Figure 21) were isolated from *K. officinalis.* Additionally, Kopsinine methochloride (**237**), demethoxycarbonylkopsin (**238**) [111], methyl chanofruticosinate (**239**), methyl 11,12-methylenedioxychanofruticosinate (**240**) [94], methyl 12-methoxychanofruticosinate (**241**), methyl 11,12-methylenedioxy-*N*_1_-decarbomethoxychanofruticosinate (**242**) [112], kopsininic acid (**243**), and (−)-11,12-methylenedioxykopsinaline (**244**) [98] were identified from the same species. Furthermore, (−)-*N*-methoxycarbonyl-11,12-methylenedioxykopsinaline (**245**) [98], (−)-*N*-methoxycarbonyl- 12-methoxykopsinaline (**246**), *N*-carbomethoxy-11-hydroxy-12- methoxykopsinaline (**247**) [113], kopsinoline (**248**) [114], (−)-12-methoxykopsinaline (**249**) [98], 11,12-methylenedioxykopsinaline N(4)- oxide (**250**) [87], kopsinine B (**251**) [115], rhazinilam (**252**) [66], and pleiocarpamine methochloride (**253**) [116] were all isolated from the twigs and leaves of chines *K. officinalis*. Compound **252** displayed a significantly inhibition effect of the human T cell proliferation, which was activated by using anti-CD3/anti-CD28 antibodies, with an IC_50_ = 1.0 μM, showing stimulation, with an IC_50_ = 1.1 μM [110]. Compound **252** was indicated to have the highest cytotoxic effect due to the presence of a hydroxyl group in C-14 and C-15 position [110].

**Figure 22:**
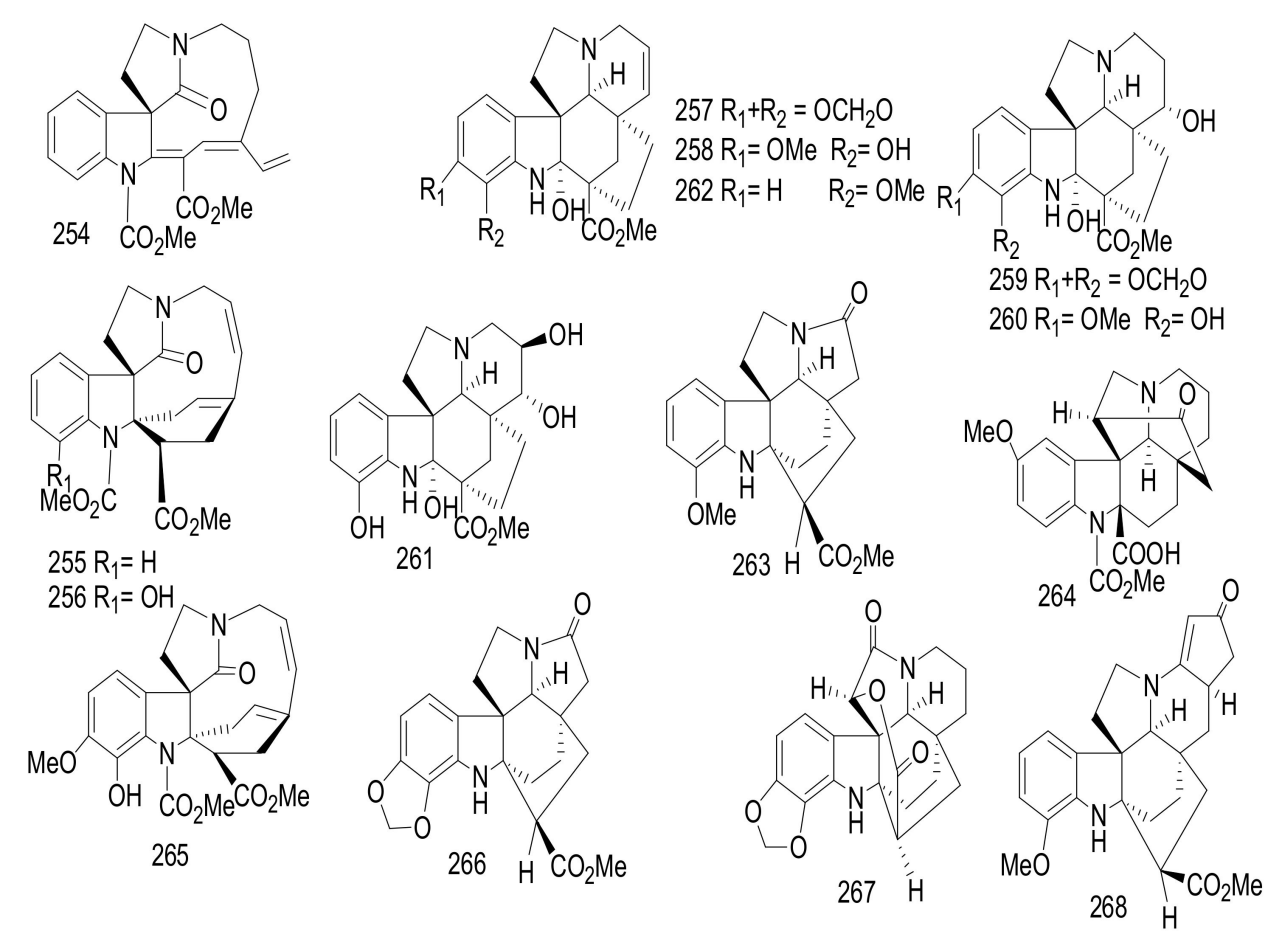
Compounds **254**–**268**.

Kopsioffines A-C (**254**–**256**) [117] (Figure 22) were isolated from the leaves and stems of *K. officinalis.* These compounds possess a relatively novel ten-membered lactam ring [117]. Additionally, five MIAs, Kopsifolines G-K (**257**–**261**) were identified from the same plant [118]. Moreover, kopsifoline A (**262**) was isolated from the aerial parts of an unidentified *Kopsia* sp. [119]. Compounds **259**–**261** exhibited cytotoxic effects against dermatoma (HS-1, A431, SCL-1, HS-4), gastric carcinoma (BGC-823), breast cancer (MCF-7), and colon cancer (SW480), with IC_50_ values in a range between 11.8 and 13.8; between 10.3 and 12.5; between 7.3 and 9.5 μM, respectively (Adriamycin, IC_50_ < 34 nM). Compound **261** showed a potent cytotoxic effect that may be due to the presence of two hydroxyl groups in the C-14 and C-15 positions, instead of one hydroxyl group at C-15 position in compounds **259** and **260**. Compounds **257**, **258** and **262** exhibited a weak cytotoxic effect with IC_50_ values > 20 μM. This may be due to the absence of a hydroxyl group in that position [118]. Compounds **254**–**256** exhibited weak inhibitory effects on yeast α-glucosidase in vitro with IC_50_ values > 50 μM [118]. Compounds **259**–**260** exhibited interesting antifungal and antimicrobial activities toward five pathogen bacteria *Escherichia coli*, *Pseudomonas aeruginosa, Enterobacter cloacae*, *Shigella dysenteriae and Klebsiella pneumoniae*), and also exhibited an antibacterial effect on the oral pathogens *Streptococcus viridans* and *Streptococcus mutans.* Netilmicin was used as a reference drug, with MIC values < 0.18 mm. 5-Flucytocine was used as a positive control with MIC values < 0.09 mM. Alkaloid **261** displayed the highest antimicrobial activity toward the tested pathogens, with an MIC value of 0.15–1.14 mM, while compounds **259** and **260** showed significant activities, with MIC values of 0.77–3.09 and 0.72–1.37 mM. Compounds **257**, **258** and **262** were inactive. The present of a hydroxyl group at the piperidine ring enhanced the anticancer and antimicrobial activity in this subtype of indoles [118]. The investigation of the aerial parts of *K. arborea* led to the isolation of three compounds: kopsiarborines A-C (**263**–**265**) [120]. Meanwhile, the study of the aerial parts of *K. officinalis* led to the reporting of three MIAs, kopsiaofficines A–C (**266**–**268**) (Figure 22) [121]. Compounds **263** and **264** showed significant cytotoxic activities against H446, H292, A549, H460, ATCC, and 95-D, with IC_50_ values < 20 μM, (Doxorubicin, IC_50_ value = 0.06 μM). Compound **264** exhibited a potent activity with IC_50_ values < 9.5 μM, and compound **265** was inactive [120]. Compound **268** exhibited a potent cytotoxicity against H446, A549, ATCC, 95-D, H460, H292, SPCA-1, and lung cancer cells, with IC_50_ values < 10 mM, while compound **266** showed some cytotoxic activity with IC_50_ value < 20 μM (Doxorubicin, IC_50_ = 13.7–33.7 nM) [121]. 

**Figure 23:**
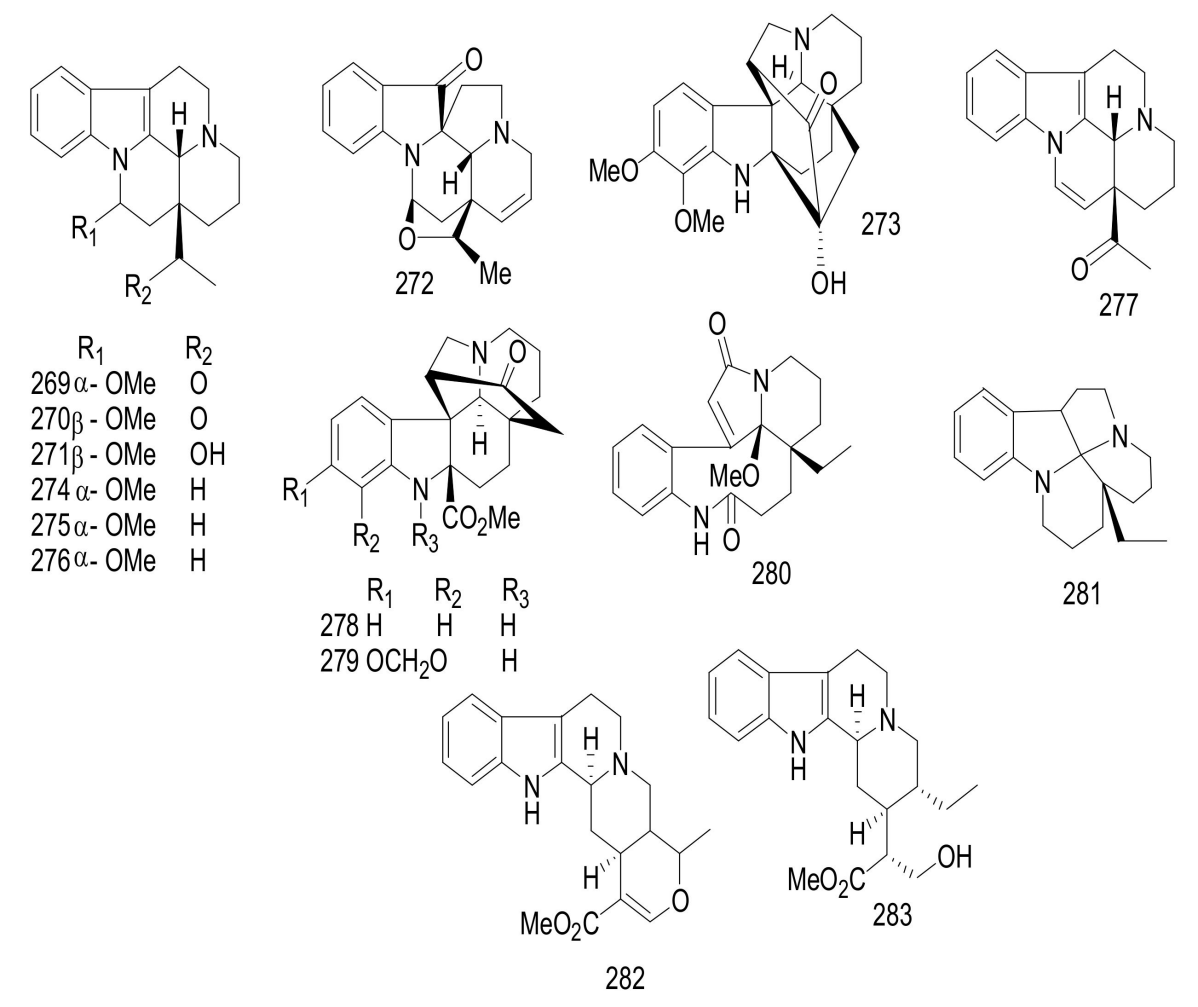
Compounds **269**–**283**.

Kopsiofficines H–L (**269**–**273**) [122] (Figure 23), together with fourteen compounds, **164**, **208**, **239**, **241**, (+)-*O*-methyleburnamine (**274**) [93], (−)-*O*-methylisoeburnamine (**275**) [123], 16-isoeburnamine (**276**) [124], 20-oxoeburnamenine (**277**) [125], methyl 11, 12-methylenedioxychanofruticosinate (**278**) [99], methyl N-(decarbomethoxy)-11, 12-(methylenedioxy) chanofruticosinate (**279**) [126], *O*-methylleuconolamm (**280**) [127], leuconodine D (**281**) [128], oxayohimban-16-carboxylic acid (**282**) [129], and 19, 20-dihydroisositsirikine (**283**) [130] (Figure 23), were identified from the stems of *K. officinalis* plant [122]. Compounds **164**, **241**, **270**, **271**, **274**, **275**, **279**, and **281** exhibited significant anti-inflammatory activity towards IL-1β, PGE2 and TNF-α at 5 μg/mL. Deametasona was used as a positive control at 10 μg/mL [122]. 

Table 1 methyl chanofruticosinate-type (**164**, **173**–**175**), aspidosoermatan-type (**169**, **199**), kopsine-type (**179**, **185**–**186**), strychnos-type (**180**, **187**), vincamine-type (**181**, **188**, **189**), paucidactine-type (**204**, **205**) and eburnane-type (**207**–**210**), all these subtypes belongs to the main class aspidospirma, and very few compounds belong to the main class of corynanthe-type indoles (Figure 4). Vincamine and methyl chanofruticosinate derivatives showed interesting biological activity.

## 4. Rauvolfia 

*Rauvolfia* (family Apocynaceae) contains 60 species. It contains trees or shrubs that are distributed in Africa, America, and Asia [131]. *Rauvolfia serpentine* is one of the most important medicinal plant that has been considered as a drug lead for a long time [132]. *Rauvolfia* has been used traditionally for the treatment of several diseases, such as high blood pressure (hypertensive), fever (malaria), arrhythmia, cancer, oxidative stress, microbial problems, intestinal spleen ailments, and various mental disorders [133]. Therapeutically, it is a source of monoterpenoid indoles, including ajmaline (antiarrhythmic), ajmalicine, yohimbine, reserpine (antihypertensive), and serpentine [133]. 

A review entitled "*Rauvolfia serpentina* L. Benth. ex Kurz. phytochemical, pharmacological and therapeutic aspects" was published in 2013 and evaluated various bioactive compounds as ajmaline, ajmalicine, deserpidine, reserpine, reserpiline, serpentine, rescinnamine and yohimbine [132]. A review entitled "Chemical and Biological Perspectives of Monoterpene Indole Compounds from *Rauwolfia* species" mentioned the compounds obtained until 2016 [134]. Another review described the structures and pharmacological potentials of the plant species *Rauvolfia tetraphylla* L. (Apocynaceae) [135].

**Figure 24:**
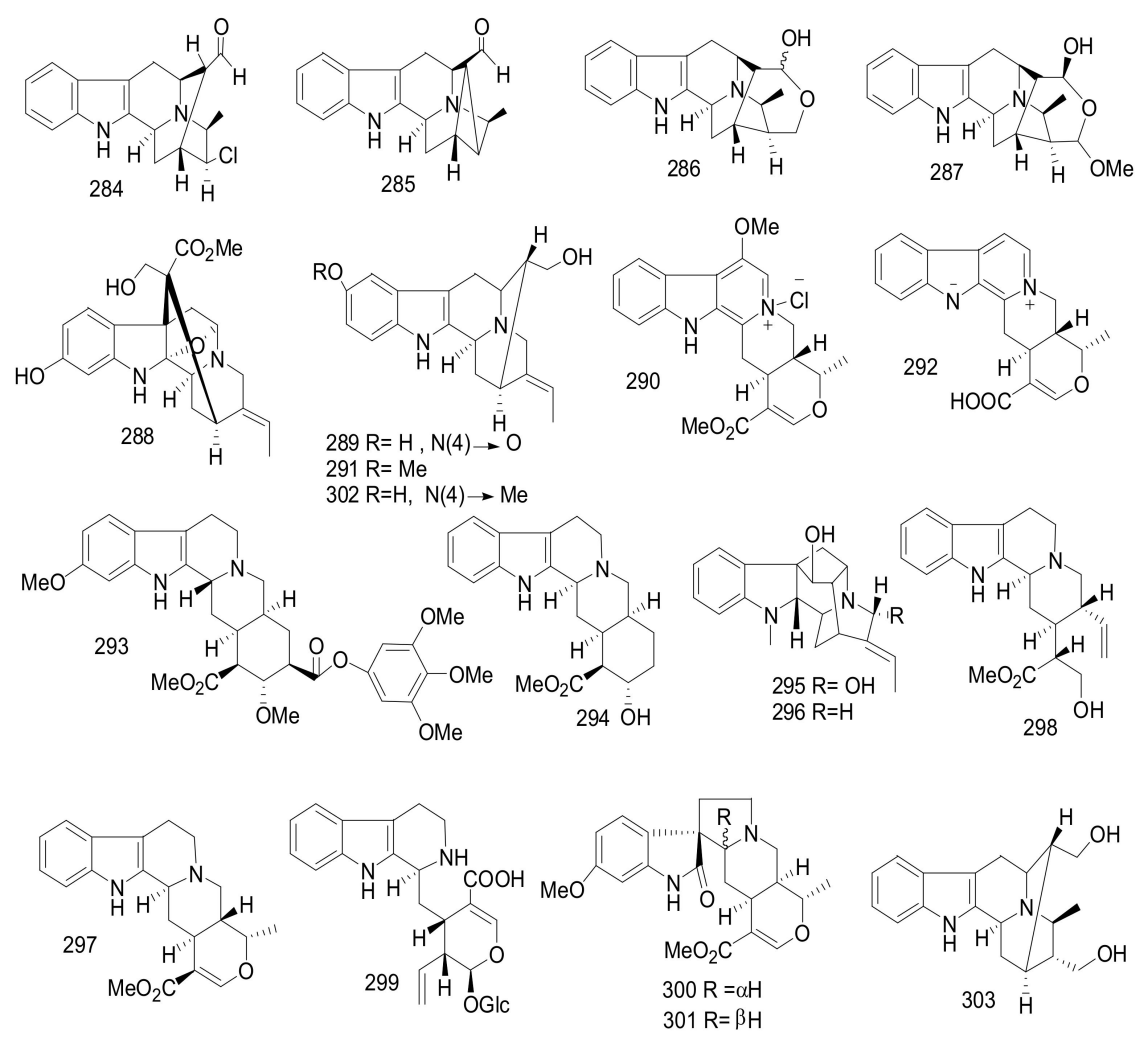
Compounds **284**–**303**.

Two normonoterpenoid indole compounds were isolated from the aerial parts of *Rauvolfia vomitoria*, rauvomines A (**284**) and B (**285**) [136] along with two known compounds peraksine (**286**) (Figure 24) [137] and alstoyunine A (**287**) [42]. Compound **285** displayed significant anti-inflammatory effects against murine macrophages (RAW 264.7), with an IC_50_ value = 39.6 μM, whereas, compounds **284**, **286** and **287** displayed moderate anti-inflammatory effects with IC_50_ values = 55.5, 65.2, and 75.3 μM, respectively, (Celecoxib, IC_50_ = 34.3 μM) [136]. Compound **285** showed a potent activity which maybe double the number of connections linking C-20 to C-16 in sarpagine-type indoles, compared with compound **284 [63].**

Three compounds, 11-hydroxyburnamine (**288**) and rauvoyunnanines A and B (**289**–**290**) were identified from Chinese *R. yunnanensis* [138]. Additionally, fourteen compounds **135** [139], lochnerine (**291**) [140], serpentinic acid (**292**) [141], reserpine (**293**) [142], (−)-yohimbine (**294**) [143], ajmaline (**295**) [143], mauiensine (**296**) [144], ajmalicine (**297**) [145], sitsirikine (**298**) [146], strictosidinic acid (**299**) [147], caboxine B (**300**) [148], isocaboxine B (**301**) [148], spegatrine (**302**) [149], and 19(*S*),20(*R*)-dihydroperaksine (**303**) [150] (Figure 24) were isolated also from chines *R. yunnanensis*. Compound **293** displayed a weak cytotoxicity against HT-29 and SW480, with IC_50_ values = 35.2 and 45.3 μM, respectively. Auranofin was used as a positive control and showed cytotoxicity with IC_50_ values = 2.5 and 3.9 μM, respectively. Compounds **294** and **299** displayed immunosuppressive activities on human T cell proliferation, with IC_50_ values = 5.9 and 5.0 μM, respectively. All compounds except **294** and **299** showed weak activities with IC_50_ values > 50 μM [138]. The metabolites were identified from genus *Rauvolfia* and were categorized under the corynanthe-type. The compounds were also classified under three subclasses including: sarpagine-type **284**–**285**, picraline-type **288** and ajmaline-type **295**–**296** and **298** [138].

## 5. Ervatamia

The *genus Ervatamia* contains 120 species. It is distributed in Asia and Australian. Of which, fifteen species and five varieties are grown in south China. *Ervatamia* is a rich source of iboga-type MIAs, which is characterized by structural novelty and biological diversity including neuroprotective, anti-tumor, and anti-addiction activities [151,152,153]. 

**Figure 25:**
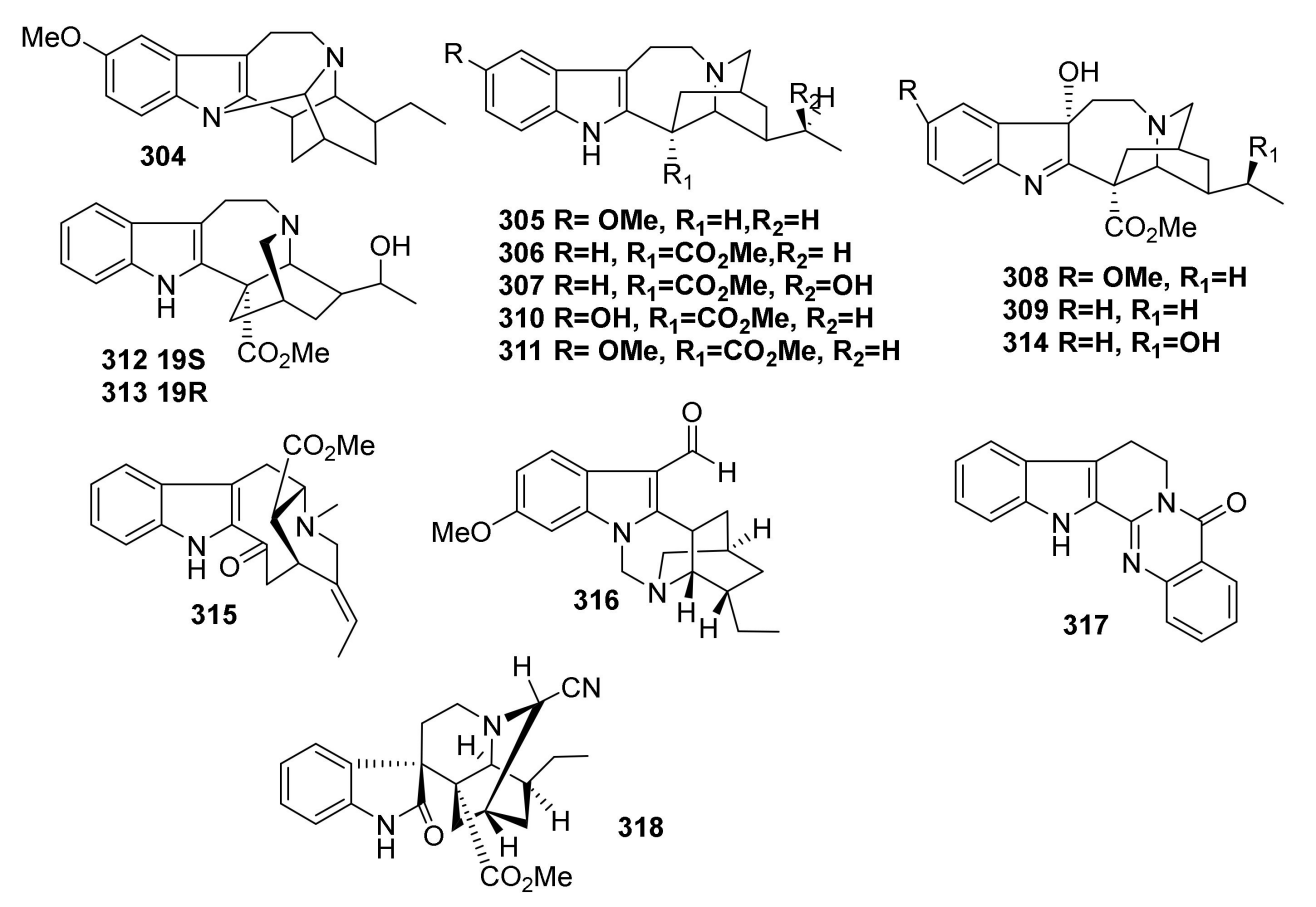
Compounds **304**–**318**.

Six Iboga-type compounds: ervataine (**304**) [151], ibogaine (**305**) [154], coronaridine (**306**) [49], heyneanine (**307**) [155], voacangine hydroxyindolenine (**308**) [156,157] and coronaridine hydroxyindolenine (**309**) [158,159] (Figure 25), were obtained from the Chinese *Ervatamia yunnanensis* [151]. 

Compound **306** exhibited significant protective effects toward MPP^+^ (1-methyl-4-phenylpyridinium) and induced damage in primary cortical neurons with an IC_50_ = 12.5 μM. Parkinson’s disease (PD) is caused by MPP^+^ a toxic agent that interferes with the function of mitochondria, thus causing neuronal damage and death. Brain-derived neurotrophic factor (BDNF) was used as a positive control and showed an inhibitory effect, with an IC_50_ value = 200 ng/mL [49]. 

Eight compounds, coronaridine (**306**) [49], coronaridine hydroxyindolenine (**309**) [158,159], 10-hydroxycoronaridine (**310**) [160], voacangine (**311**) [153], 19(*S*)-heyneanine (**312**) [160], 19(*R*)-heyneanine (**313**) [161], heyneanine hydroxyindolenine (**314**) [162], and vobasine (**315**) [163], were identified from the stems of *E. hainanensis*. Compounds **306**, **309**–**315** displayed acetylcholinesterase inhibitory activities. Compounds **306** and **311** displayed a potent cholinesterase inhibitory effect, with IC_50_ values = 8.6 and 4.4 μM, respectively. Galantamine was used as a reference drug, with an IC_50_ = 3.2 μM, that is used for Alzheimer’s disease [164]. Compound **310** possessed a hydroxyl group at the phenyl moiety, which was replaced by proton in compound **306**. This led to a decrease in the inhibitory activity of AChE in **306** compared to **310**. The methoxy group at the phenyl moiety in **311**, led to an improvement in the activity. This indicated that the electron-donor substituents attached at the phenyl group were important for the improvement of AChE inhibition [164]. 

Ervachinine E (**316**) [165] and rutaecarpine (**317**) [166] were isolated from *E. chinensis* [165]. It displayed moderate antitumor activities against HL-60, SMMC-7721, A-549, and SW480 cancer cells, with values of IC_50_ ranging between 6.59 and 14.70 μM. (Cisplatin, IC_50_ values between 1.00 and 26.75 μM) [165]. 

The compound Ervahainine A (**318**), an oxindole derivative that is cyano-substituted, was identified from the twigs and leaves of *E. hainanensis* [167]. Compound **318** showed growth inhibitory activities toward HepG2 and HepG2/ADM cells with IC_50_ values of 12.47 ± 0.24 and 17.68 ± 0.31 μM [167].

**Figure 26:**
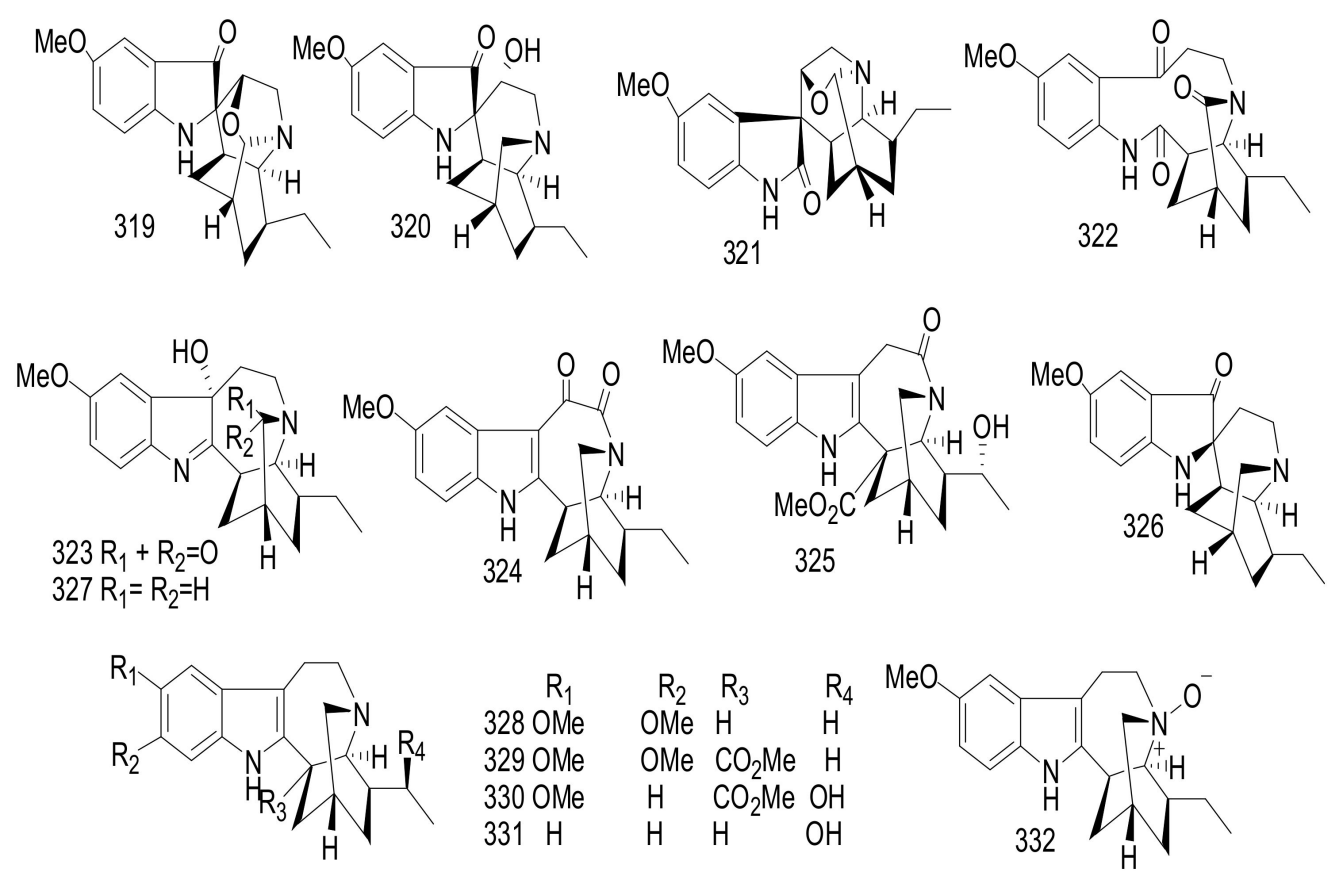
Compounds **319**–**332**.

Seven new iboga-type derivatives: ervaoffines A–D (**319**–**322**), (7*S*)-3-oxoibogaine hydroxyindolenine (**323**), ibogaine-5,6-dione (**324**), and 19-*epi*-5-oxovoacristine (**325**), along with ten compounds, **305**, **307**, **311**, iboluteine (**326**) [168], (7*S*)- ibogaine hydroxyindolenine (**327**) [157], ibogaline (**328**) [169], conopharyngine (**329**) [170], voacristine (**330**) [171], 19*S*-hydroxyibogamine (**331**) [172], and ibogaine *N*_4_-oxide (**332**) [173,174] (Figure 26), were isolated from the twigs and leaves of *E. officinalis*.

**Figure 27:**
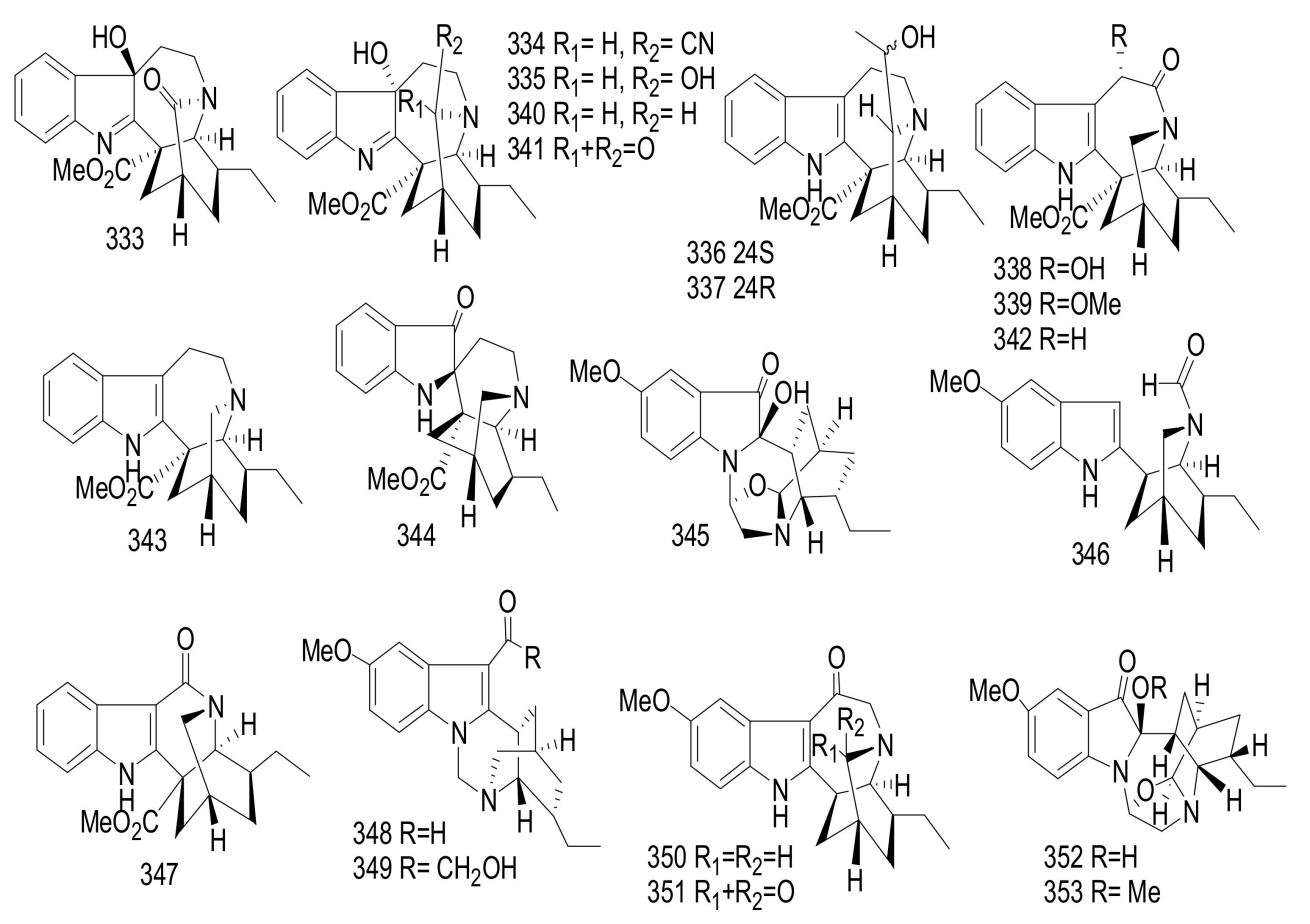
Compounds **333–353**.

Seven compounds, 3-oxo-7*R*-coronaridine hydroxyindolenine (**333**), 3*S*-cyano-7*S*-coronaridine hydroxyindolenine (**334**), 3*R*-hydroxy-7*S*-coronaridine hydroxyindolenine (**335**), 3*S*-(24*S*-hydroxyethyl)-coronaridine (**336**), 3*S*-(24*R*-hydroxyethyl)-coronaridine (**337**), 5-oxo-6*S*-hydroxycoronaridine (**338**) and 5-oxo-6*S*-methoxy-coronaridine (**339**) [175], along with six others, **306**, 7*S-*coronaridine hydroxyindolenine (**340**) [176], 3-oxo-7*S*-coronaridine hydroxylindolenine (**341**) [177], 5-oxocoronaridine (**342**) [177], 3-oxocoronaridine (**343**) [178] and pseudoindoxyl coronaridine (**344**) [177], (Figure 27) from identified from twigs and leaves of *E. hainanensis* [175]. 

Another study on the twigs and leaves of *E. officinalis* led to the reporting of three MIAs, ervaoffines E–G (**345**–**347**) [179], and six compounds **306**, **342**, lirofoline A (**348**), lirofoline B (**349**) [172], 6-oxo-ibogaine (**350**) [180], and 8-oxo-ibogaine lactam (**351**) [179,180,181]. Compound **347** showed a significant neuroprotective effect towards damage induced by oxygen-glucose deprivation (OGD) of the cortical neurons cultured of ischemic stroke in vitro, with an IC_50_ = 100 μM, Neuroserpin was used as a reference drug, with an IC_50_ = 20 ng/mL [179]. Two compounds were obtained from the roots of *E. chinensis*, erchinines A and B (**352**,**353**) [63]. Both compounds **352** and **353** displayed a potent significant antibacterial activity toward *Bacillus subtilis* which was better than that of the antibacterial drugs fibraurtine with an MIC = 25 μM and berberine with an MIC = 12.5 μM that are derived from plant. Additionally, compound **352** displayed an equal antifungal effect against *(Trichophyton rubrum*) to the reference drug griseofulvin, with an MIC = 6.25 μM.

**Figure 28:**
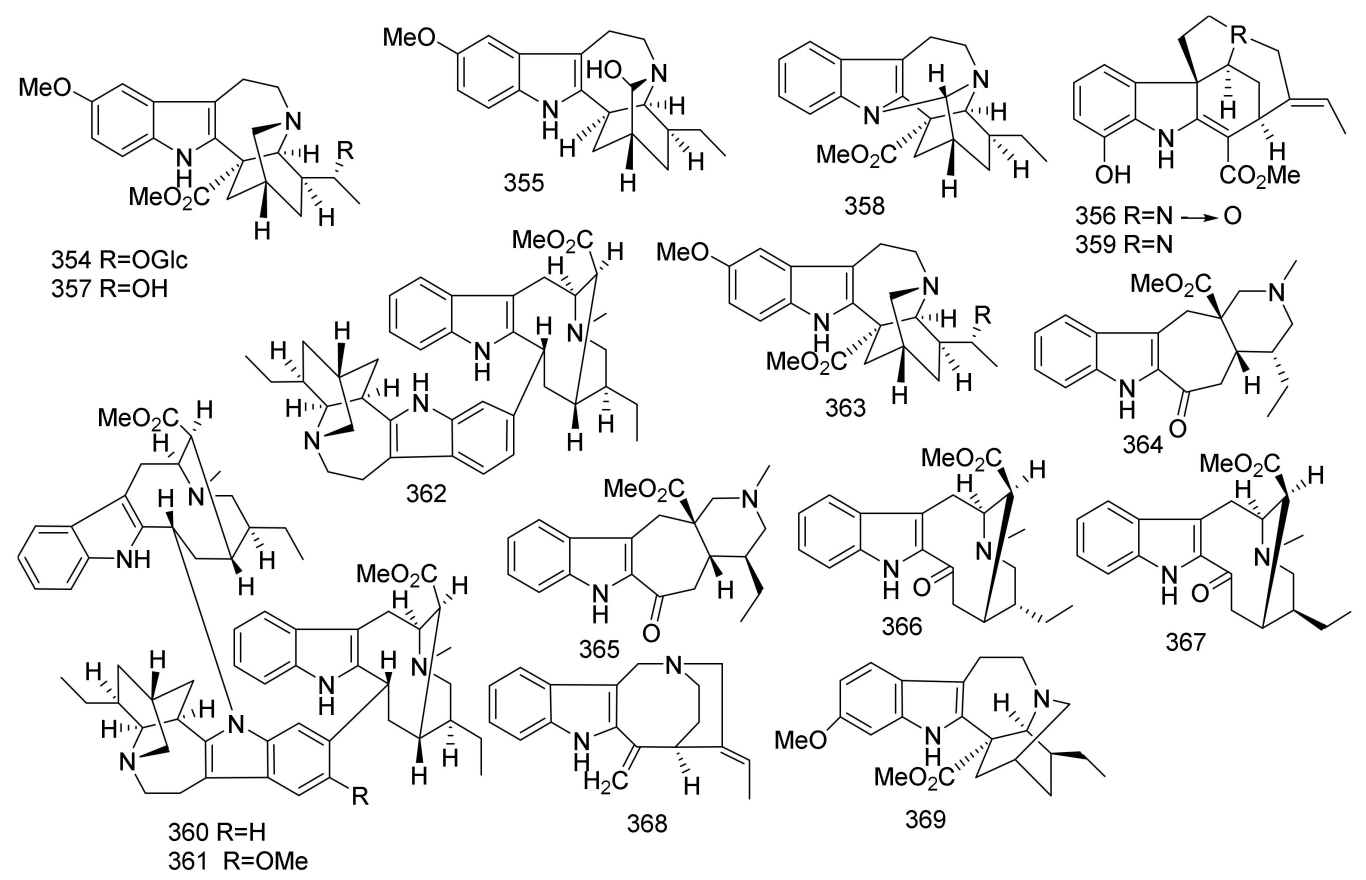
Compounds **354**–**369**.

Ervapandine A (**354**) [182], 3*R*-hydroxyibogaine (**355**) [182], and 12-hydroxyakuammicine *N*_4_-oxide (**356**) [182], along with four known ones, **313**, **305**, 19-*epi*-voacristine (**357**) **[183]**, taberdivarine I (**358**) [184] and 12-hydroxyakuamicine (**359**) [185], (Figure 28) were identified from the leaves and twigs of Chinese *E. pandacaqui* [182]. 

**Figure 29:**
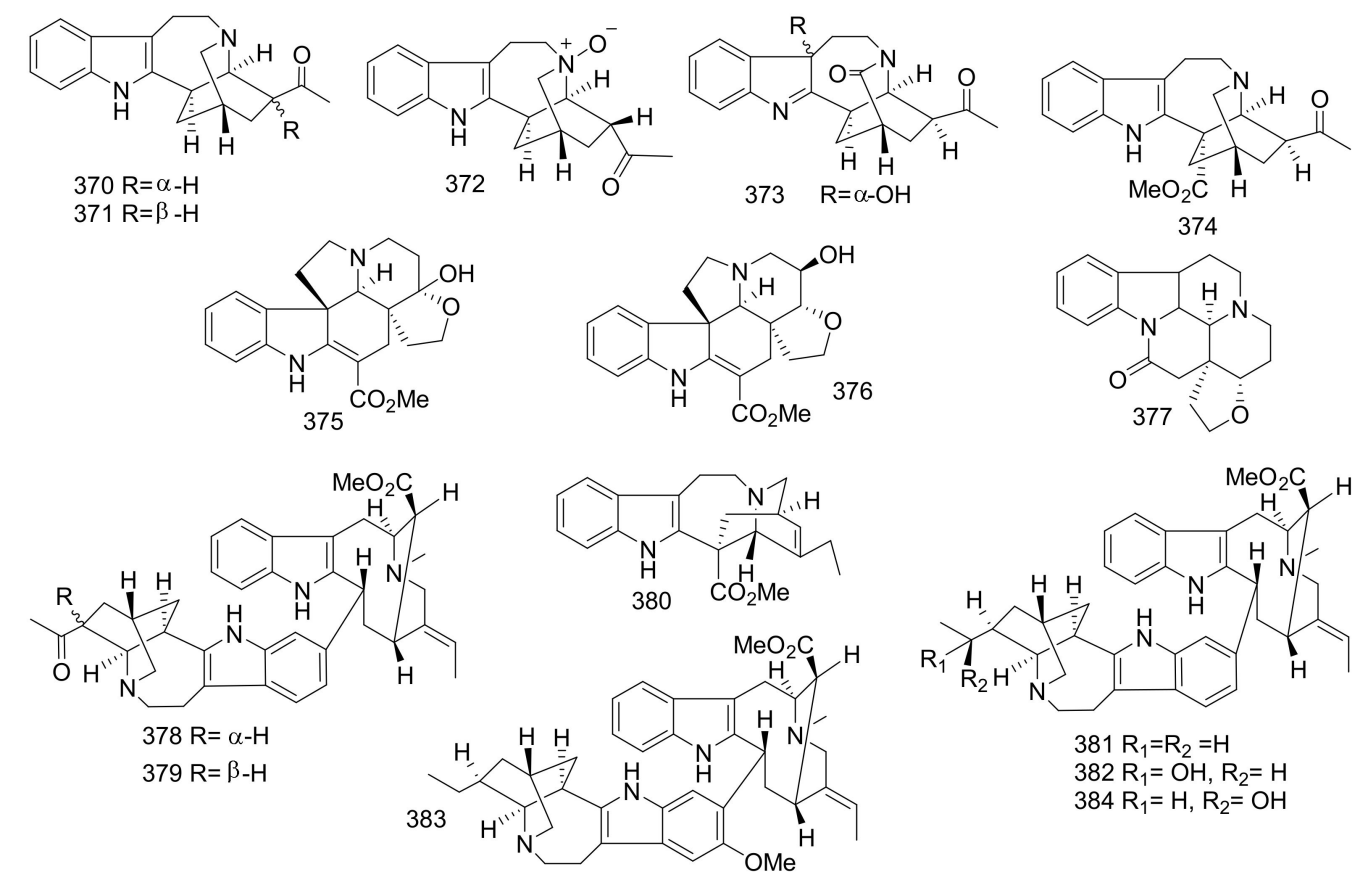
Compounds **370**–**384**.

Liu et al. (2018) [186] studied the roots of *E. divaricate* and identified two unprecedented trimeric MIAs, Ervadivamines A (**360**) and B (**361**), together with the dimeric compound, 19,20-dihydroervahanine A (**362**), (Figure 29) and two monomeric ones, ibogaine (**305**) and Ibogamine **(363**) **[187]**. Compound **359** displayed a moderate cytotoxic effect against MCF-7, with an IC_50_ value = 33.61 μM [182]. Compound **360** showed a significant positive cytotoxicity against MCF-7, A-549, HT-29 and HepG2/ADM and showed potent effect against HepG2/ADM, with an IC_50_ value = 12.55 ± 0.54 μM (Adriamycin, IC_50_ = 45.70 ± 2.15 μM) [186]. 

Two pair of MIAs epimers composed of, ervatamine (**364**), [188] 20-*epi*-ervatamine (**365**), [188] dregamine (**366**), and [188] tabernaemontanine (**367**) [188] and two compounds, apparicine (**368**) [189] and isovoacangine (**369**) [190], were isolated from *E. yunnanensis* [191].

The *Ervatamia* genus is known to produce iboga-type indole derivatives, which contain two subclasses, flabelliformide-type (**364**, **365**) and apparicine-type (**368**) (Figure 28), with compounds belongonging to the main class corynathe. The iboga-type showed an interesting bioactivity in the nervous system.

## 6. Tabernaemontana

The *Genus Tabernaemontana* (subfamily Rauvolfioideae) contains 110 species, which are distributed throughout tropical and subtropical regions. Thirty species are grown in Brazil, whereas, 44 species were grown in America and the rest in different places around the world. Traditionally, the plants of this genus have been used for the treatment of hypertension, sore throat, and abdominal pain [6,192]. A review article entitled “Brazilian *Tabernaemontana* genus: indole compounds and phytochemical activities” activities was published in 2016 [6]. It concerned in the monomeric and dimeric MIAs reported from the genus. A review article entitled: A review on *tabernaemontana* spp.: Multipotential medicinal plant, shows the MIAs reported from this genus until 2015 [6]. 

Conodusine A-E (**370**–**374**), apocidine A (**375**) and B (**376**), conoduzidine A (**377**), tabernamidine A (**378**) and B (**379**) (Figure 29) were isolated from the Malaysian stem-bark of *Tabernaemontana corymbose* malaysian [193]. Additionally, thirty-two compounds were also identified from the same plant, including **307**, **314**, **338**, (+)-catharanthine (**380**), tabernamine (**381**) [194], 19′(*S*)-hydroxytabernamine (**382**) [195], and 19′(*R*)-hydroxytabernamine (**383**) [195]. 16′-decarbomethoxyvoacamine (**384**) **[180]** (Figure 29). The chemical structures were determined based on analysis of the NMR and MS spectral data. However, compounds **370, 372, 374, 375** and **377** were confirmed by X-ray diffraction analyses. **371** and **371** belong to iboga alkaloids and tabernamidine B is an iboga-containing bisindole. Tabernamidine B (**379**) is notable for the presence of an α-substituted acetyl group at C-20 of the iboga carbon skeleton. The absolute configuration of (+)-conodusine E was based on an analysis of the ECD data in correlation with (−)-heyneanine and X-ray analysis. Compounds **381**–**384** exhibited growth inhibitory effects against drug-sensitive KB/S, with an IC_50_ value < 4.7 μM and vincristine-resistant (KB/VJ300) cells with an IC_50_ value < 4.2 μM. For that type of human oral cancer cell lines, vincristine was used as a reference drug with an IC_50_ value < 1.8 nM [193].

Two compounds, isoakuammiline (**385**) and 18-hydroxypseudovincadifformine (**386**) [196], have been reported from the American fruits of *T. litoralis.* Additionally, five compounds 3,19-oxidocoronaridine (**387**) [196], strictosidine (**388**) [196], **306**, heyneanine **307**, and tabersonine (**17**), have been identified from the same species [196]. Strictosidine is the major alkaloid in fruit arils, however in the capsule strictosidine it was converted to mainly iboga and pseudoaspidosperma alkaloids. However, in seeds, strictosidine was converted to both iboga and aspidosperma alkaloids, but the only major iboga alkaloid, coronaridine, was not substituted, whereas in fruit capsule coronaridine was oxidized to form heyneanine and 3,19-oxidocoronaridine. 

**Figure 30:**
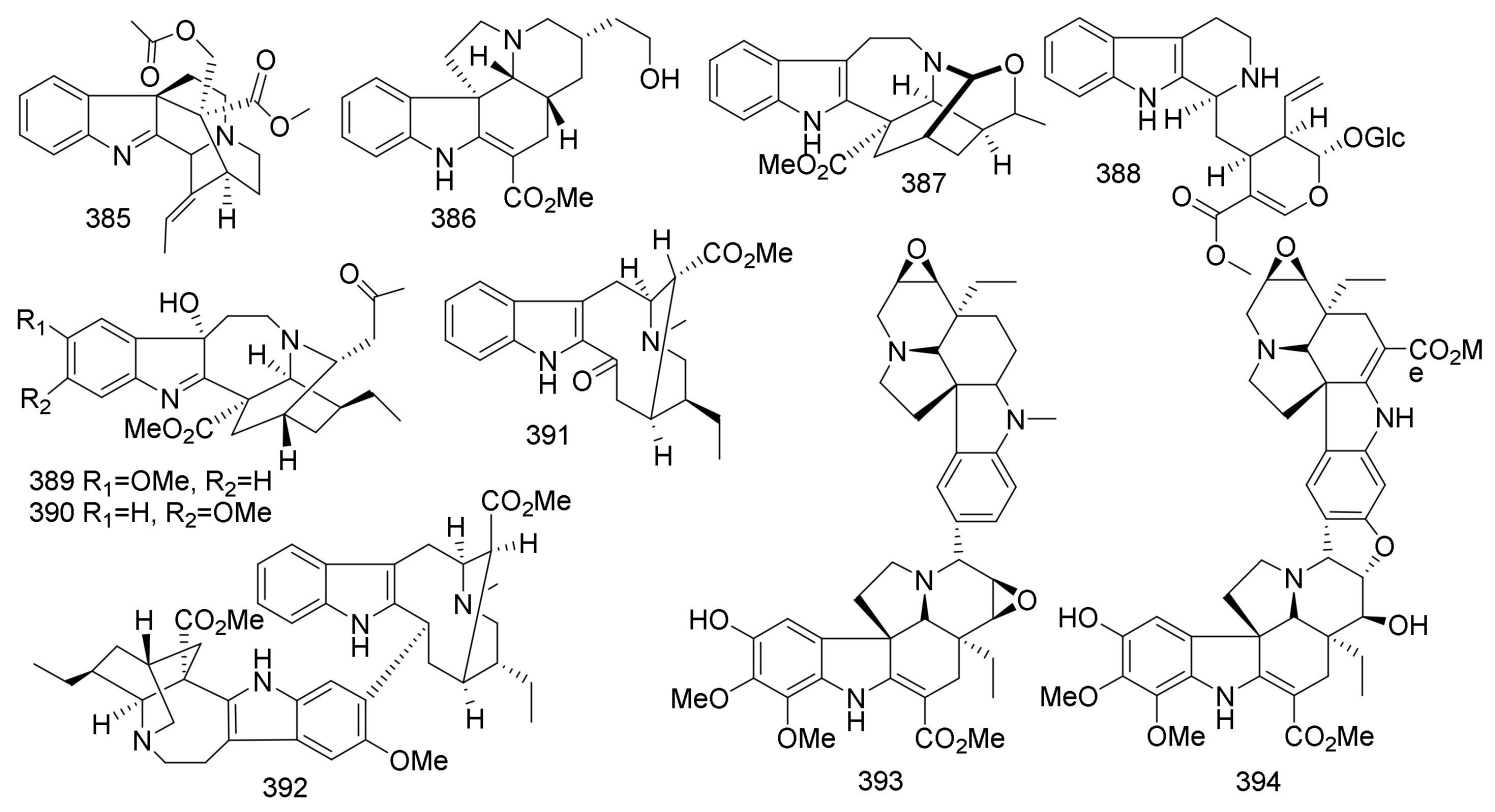
Compounds **385**–**394**.

Tabervarines A (**389**) and B (**390**) [197], **311**, **369,** vobasidine C (**391**) [198], **311**, **368**, ervadivaricatine B (**392**) [187], pedunculine (**393**) [199], tabernaemontanine (**367**) [198] and polyervine (**394**) [200] were published from the twigs and leaves of the Chinese *T. divaricate* (Figure 30). Compounds **388** and **389** exhibited a weak cytotoxic effect against cancer MCF-7, SMMC-7721, HL-60, A-549, and SW480 cells at a value > 40 μM [197].

**Figure 31:**
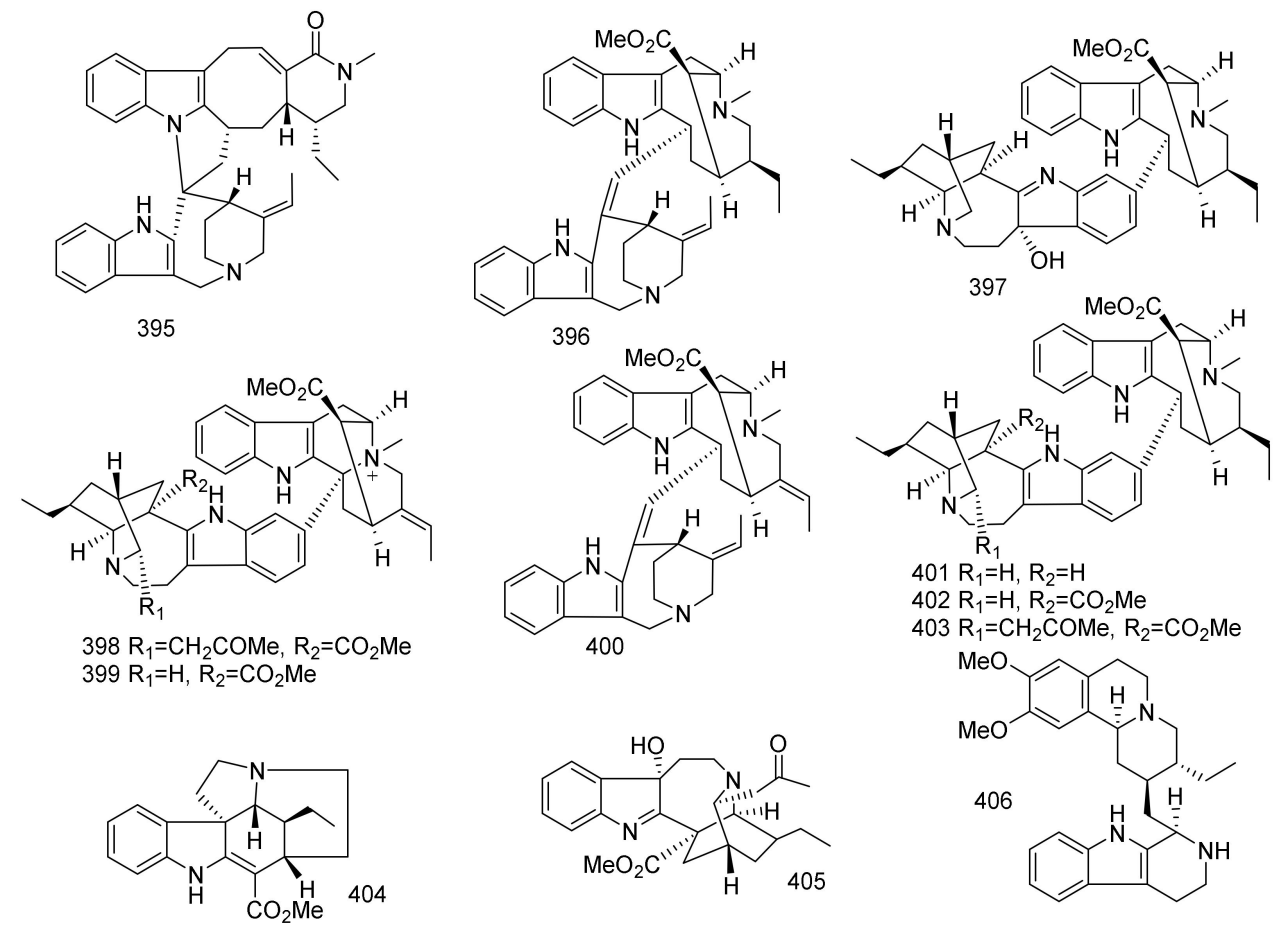
Compounds **395**–**406**.

**Figure 32:**
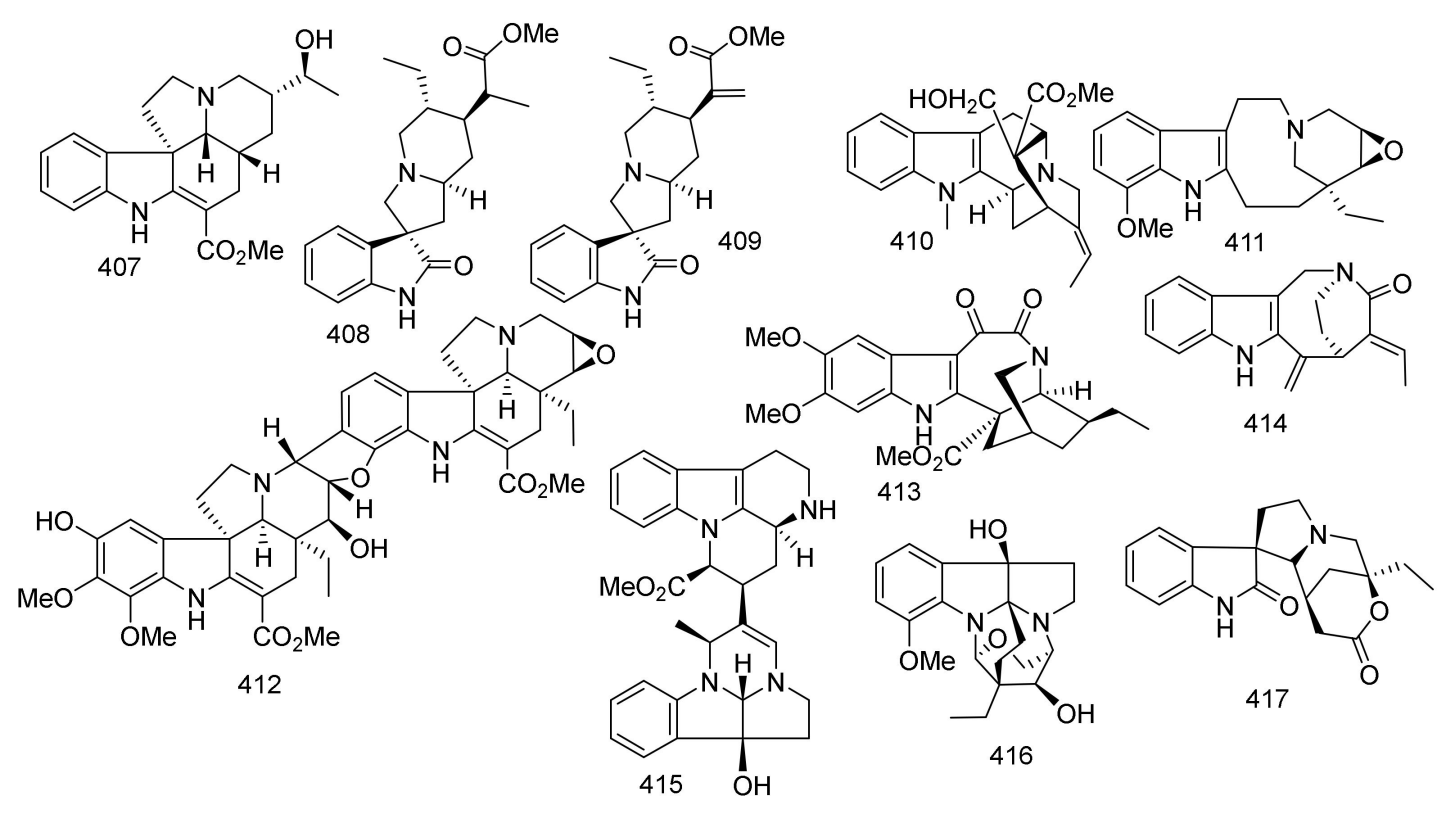
Compounds **407**–**417**.

Four new bisindole compounds, flabellipparicine (**395**), 19,20-dihydrovobparicine (**396**), 10′- demethoxy-19,20-dihydrovobatensine D (**397**) and 3′-(2-oxopropyl)ervahanine A (**398**) [201], together with ten known compounds, **381**, **368**, ervahanine A (**399**) [202], vobparicine (**400**) [203], 19,20-dihydrotabernamine (**401**) [204], 19,20-dihydrotabernamine A (**402**) [205], taberdivarine E (**403**) [184], tubotaiwine (**404**) [206], hydroxy-3-(2-oxopropyl)coronaridineindolenine (**405**) [204], and deoxytubulosine (**406**) [201] (Figure 31) were identified from the stems of *T. divaricate*. Compounds **368**, **395**–**403** and **406** exhibited cytotoxic activities against MCF-7 and A-549 with IC_50_ values < 8.1 μM. Compound **406** exhibited the highest effects against MCF-7 and A-549 with IC_50_ values of 0.1 and 0.2 nM, respectively. 7-ethyl-10-hydroxycamptothecin (SN38) was employed as a positive control and showed cytotoxic effect, with an IC_50_ value < 2 nM [201]. The presence of β-carboline benzoquinolizidine nucleus played an important role in increasing the cytotoxicity in **406**, whereas, compounds (**368** and **395**–**403)** possessed two NH indolic group [201]. 

(3*R*,7*S*,14*R*,19*S*,20*R*)-19-hydroxypseudovincadifformine (**407**) [207], 17-demethoxy-hydroisorhyn chophylline (**408**) [208], 17-demethoxy-isorhynchophylline (**409**) [208], voachalotine (**410**) [171], 12-methoxyl-voaphylline (**411**) [209], and conophylline (**412**) [209] (Figure 32) were isolated from the branches and leaves of Chinese *T. bufalina*. Compound **412** showed potent cytotoxic activities against B16 and MDA-MB-231 cells with IC_50_ values of 0.13 and 8.9 μM, respectively. Gambogic acid was used as a positive control with IC_50_ values 22.1 and 13.5 μM, respectively [207].

Two compounds, 5,6-dioxo-11-methoxy voacangine (**413**), and (−)-apparicin-21-one (**414**), and heyneanine (**307**), were identified from the fruits of cameroonean *T. contorta* [210] lipopolysaccharides (LPS)-stimulated RAW 264.7 macrophage cells. BAY 11-7082 was used as positive control with 10 μM [210]. Tabernabovines A–C (**415**–**417**) were isolated from *T. bovina* [211]. Compound **415** displayed potent inhibitory activity of NO production in LPS-stimulated RAW 264.7 macrophages with IC_50_ value 44.1 value μM. l-NMMA was used as a positive control and showed an inhibitory effect with IC_50_ value = 48.6 μM [211].

Previous studies have proven that various bisindole compounds have more effect than monomeric indole compounds, including the dimeric indoles such as (euburnane–aspidospermatan, euburnane–ibogan, akuammidine–ibogan, aspidospermatan– aspidospermatan and vobasine–strychnan) type compounds. Interestingly, dimeric indoles showed more cytotoxicity than the monomeric units. 

The *Tabernaemontana* genus produced iboga type indoles, which contained four subclasses, such as vincamine-type, apparicine-type and akuammidine, these compounds which belongs to the main class aspidosperma and corynanthe, respectively.

## 7. Rhazya

**Figure 33:**
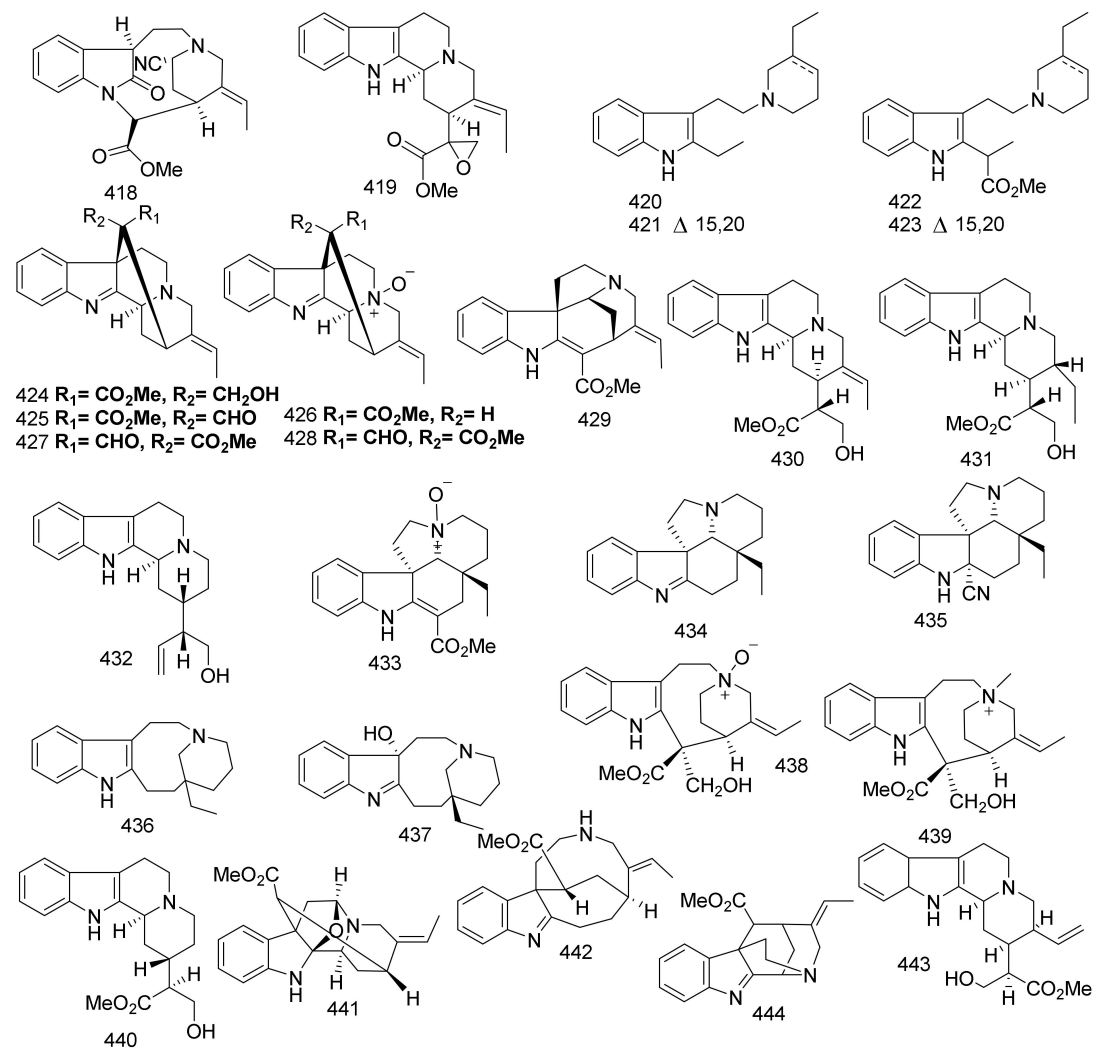
Compounds **418**–**444**.

*Rhazya* comprises two species, *Rhazya stricta* (*R. stricta*) and *Rhazya orientalis* (*R. stricta*) [212]. *R. orientalis* grown in western Thrace and northeastern Turkey [213] whereas, *R. stricta* is grown in South Asia (Afghanistan, Pakistan and India) and on the Arabian Peninsula (Saudi Arabian, Qatar, UEA, Iraq) and Iran. *Rhazya* is a rich source of indole-containing compounds. Traditionally, it is has been used to cure various diseases, such as fever, rheumatism, inflammation, skin infections, sore throat, diabetes, and stomach disorders. For example, strictanol, sewarine, tetrahydrosecamine vallesiachotamine and tetrahydrosecaminediol exhibit anticancer properties [213,214,215,216,217,218]. A recent study on the aerial parts of *R. stricta* by Ahmad et al. [215], several MIAs were isolated including, three new, secopleiocarpamine A (**418**), 16,17-Epoxyisositsirikine (**419**), and 2-Ethyl-3[2-(3-ethyl-1,2,3,6-tetrahydropyridine)ethyl]-indole (**420**) [215] (Figure 33), five previously reported compounds from other Apocynaceae genera (**126**, **127**, **133**, **298** and **404**), and a number of previously isolated MIAs from the same species: 2-ethyl-3[2-(3-ethylpiperidine)ethyl]-indole (**421**), tetrahydrosecodine (**422**), 16,17-dihydrosecodine (**423**) [216], deacetylakuammilin (**424**) [217], rhazimal (**425**), strictamine-*N*-oxide (**426**) [218], rhazinaline (**427**) [212], rhazinaline *N*_b_-oxide (**428**) [219], akuammicine (**429**) [220], 16*R*-*E*-isositsirikine (**430**) [221], dihydrositsirikine (**431**) [222], antirhine (**432**) [129], vincadifformine *N*_(4)_-oxide (**433**) [223], eburenine (**434**) [93], winchinine B (**435**), quebrachamine (**436**) [224] and strictanol (**437**) (Figure 33) [215,225] were isolated from *R. stricta.* Furthermore, 16-*epi*-stemmadenine-*N*-oxide (**438**) (Figure 33), stemmadenine-*N*-methyl (**439**), and 20-*epi*-antirhine (**440**) were reported from *R. stricta* [226]. Additionally, isopicrinine (**441**) was isolated from the leaves of *R. stricta*, collected from Bahra, Saudi Arabia [227]. Abdul-Hameed *et al.* (2021) [228] identified two new indole alkaloids named, epirhazyaminine (**442**) and 20-*epi*-sitsirikine (**443**), together with five known compounds, **430**, **432**, **434**, **437** and strictamine (**444**) were obtained from the aerial parts of *R. stricta*, collected from AL-Madinah city, Saudi Arabia [228]. Compounds **418, 422**, **428, 432**, **434**, and **436** exhibited moderate growth inhibitory activities toward Candida strains (*C. guilliermondii, C. albicans, C. krusei, C. lusitaniae and C. glabrata*) with MIC values ranging from 3.125 to 50 μg/mL. (Amphotericin B, MIC value < 1 μg/mL) [213]. Compound **438** displayed a cytotoxic effect against HCT-116, PC-3, and HepG2, with IC_50_ values = 2.20, 2.25, and1.9 μM, respectively, (Cisplatin, IC_50_ values ≤ 0.90 μM). Furthermore, compound **439** significantly hindered of the cancer cells to migration and preventing the wound healing at 24 and 48 h (from 81 and 77% to 68 and 46%, respectively). It also inhibited proliferation and prevented cell migration of all cancer cell was evaluated, with an IC_50_ = 70 μM [223]. Compound **441** displayed a potent cytotoxic effect towards MCF-7, with an IC_50_ value = 240 μM [224]. Compounds **430**, **432**, **434**, **437,** and **442**–**444** displayed weak activities against three cancer cell lines (HCT-116, PC-3, and HepG2), with IC_50_ in the range of 45.0 ± 0.012 and 85.0 ± 0.068 μM against HCT-116, IC_50_ in the range 39.0 ± 0.012 and 87.0 ± 0.068 μM against PC-3, and IC_50_ in the range 72.0 ± 0.164 and 87.0 ± 0.032 μM against HepG-2μM) against HepG-2 [225]. The *Rhazya* genus contains many MIAs subclasses, such as secodine-type (**420**–**424**), akuammiline-type (**426**), akummicine-type (**428**) and picrinine-type (**441**), (Figure 3), with compounds belonging to the main classes aspidosperma and corynanthe.

## 8. Biosynthesis of Monoterpenoid Indole Alkaloids

Monoterpenoidal indoles are obtained from the reaction of tryptamine with secologanin terpenoid. Condensation of tryptamine with Secologanin produces strictosidine by the Mannich-link reaction. The deglycosylation of strictosidine converts it to a hemiacetal. Opening the hemiacetal led to forming an aldehyde group, which then reacts with the (N-4) secondary amine of strictosidine to form 4,21-dehydrocorynanthenine. Allylic isomerization moves the double bond of vinyl to a conjugation with iminium nitrogen that generates dehydrogeissoschizine, which is then cyclized to form cathenamine. The reduction of cathenamine in the presence of NADPH forms ajmalicine (corynanthe-type) [229]. 

**Scheme 1:**
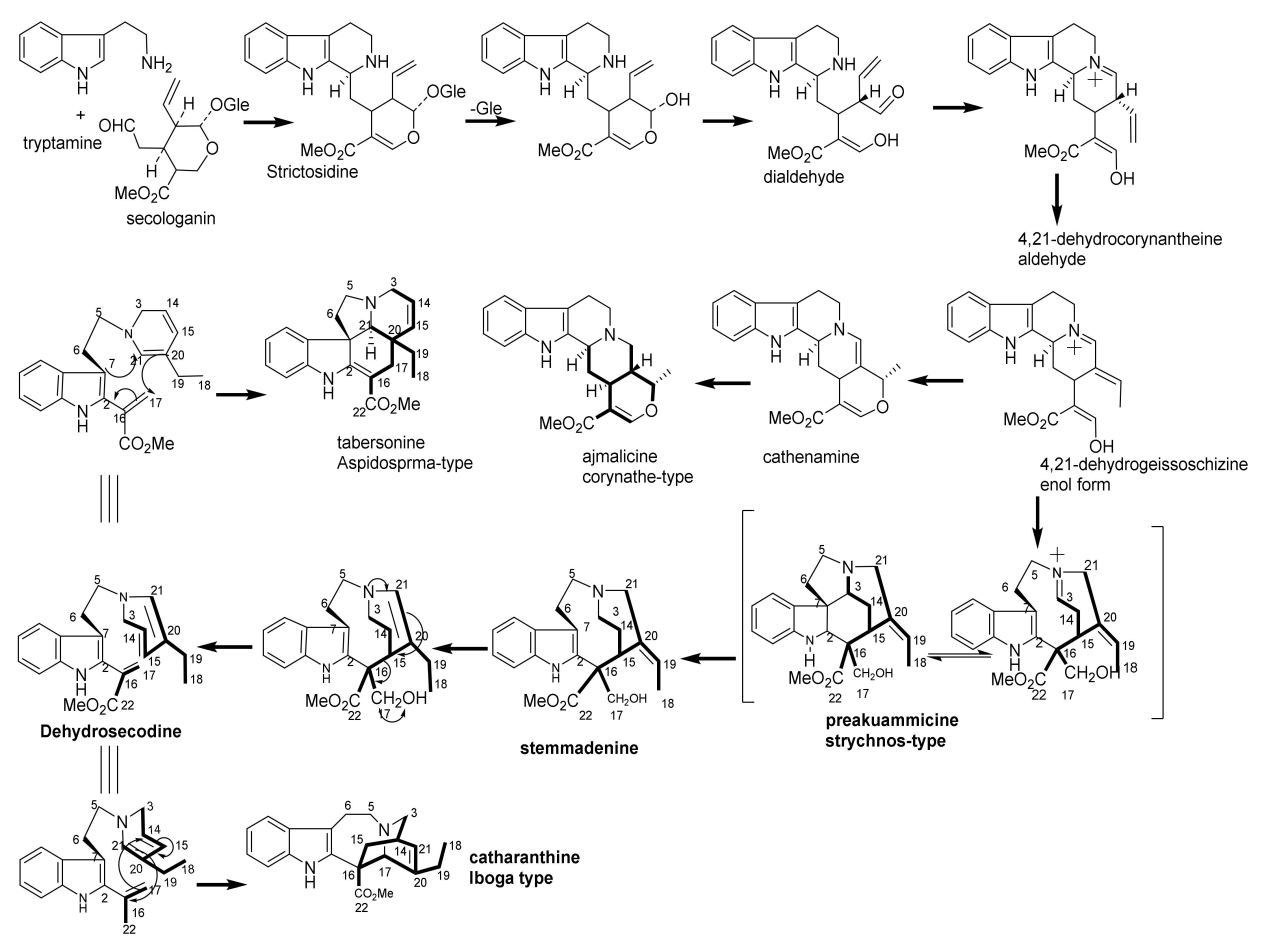
Biosynthesis of corynanthe, aspidosperma and iboga indoles.

The formation of Preakuammicine occurs from dehydrogeissoschizine. Preakuammicine intermediate (strychnos-type) is the common precursor of the strychnos, aspidosperma and iboga indole alkaloids. Preakuammicine reduced to form stemmadenine, then rearranged to form the acrylic ester dehydrosecodine, which is a common intermediate for iboga and aspidosperma skeletons. Tabersonine (aspidosperma type) and catharanthine (iboga type) are formed the Diels-Alder reaction (Scheme 1) [229].

**Scheme 2:**
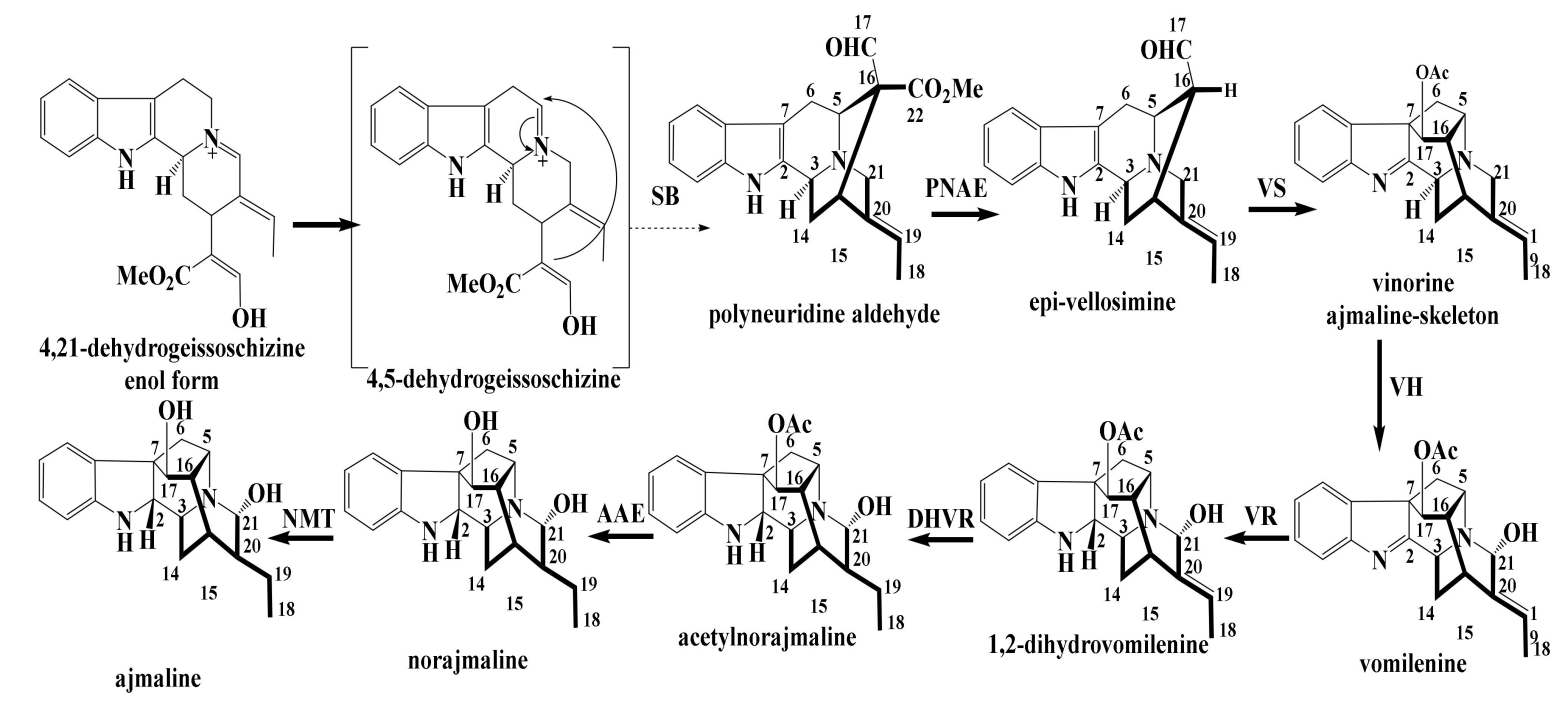
Biosynthesis of ajmaline indole alkaloids. (SB) Sarpagan bridge enzyme; polyneuridine aldehyde reductase (PNAE), vinorine synthase (VS), vinorine hydroxylase (VH), vomilenine reductase (VR), dihydrovomilenine reductase (DHVR) 17-*O*-acetyl-ajmalanesterase (AAE), norajmaline-*N*-methyltransferase (NMT).

Polyneuridine aldehyde (sarpagan type) is an intermediate compound of the ajmaline pathway. The possibility of a mechanism where the sarpagan bridge enzyme converts an isomer of 4,21-dehydrogeissoschizine to polyneuridine aldehyde is shown (Scheme 2). Polyneuridine aldehyde methyl ester is hydrolyzed by polyneuridine aldehyde esterase, generating an acid which decarboxylates, to yield *epi*-vellosamine. *Epi*-vellosamine transforms to the ajmaline alkaloid vinorine. The hydroxylation of vinorine to vomilene is caused by the vinorine hydroxylase enzyme. After formation of vomilene, two step reduction occurs. First, the indolenine bond is reduced by an NADPH enzyme to yield 1,2-dihydrovomilenene. The second step, reducing the 1,2-dihydrovomilenene to acetylnorajmaline by a 1,2-dihydrovomilenene reductase enzyme. The acetyl linkage of acetylnorajmaline is hydrolyzed by acetylesterase to yield norajmaline. Finally, the production of ajmaline by N-methyl transferase of a methyl group at the indole nitrogen of norajmaline occurs (Scheme 2) [229,230].

It is noteworthy to mention that, sarpagine, ajmaline, and macroline alkaloids are biosynthetically similar or all derived from the same origin. Whereas, sarpagine can be converted into macroline by means of Michael addition [231], on the other hand macroline can be converted into sarpagine by through a retro-Michael reaction [231,232,233]. Similarly, some sarpagine-containing alkaloids can be converted into ajmalines under strong acidic conditions, which refers to the great similarity between them [233].

## 9. Conclusions and Future Prospectives

Natural products have an unprecedented molecular conformity with a diversity of functionalities. These characteristics enable them to produce biological effects, which validates the initial step for a drug lead. In recent years, the majority of new drugs reported have been natural or originated from natural sources. Alkaloids are an important source of drugs. It is noteworthy that, many alkaloids displaying fascinating molecular structures with diverse physiological and pharmacological effects have been isolated from plant families. The Apocynaceae family has been noted as a unique producer of biologically active natural metabolites such as vincristine, vinblastine, reserpine and yohimbine. This review is interested in discussing the metabolites produced from six genera belong to the family Apocynaceae. These six genera contain 400 species, which represent 20% of the Apocynaceae family. Only 30 species, which represent 7.5% of the total species of the six genera were studied. Chemical investigation of these genera led to the reporting of 444 MIAs, in the period between 2010 until December 2020, which were discussed in this review.

**Figure 34:**
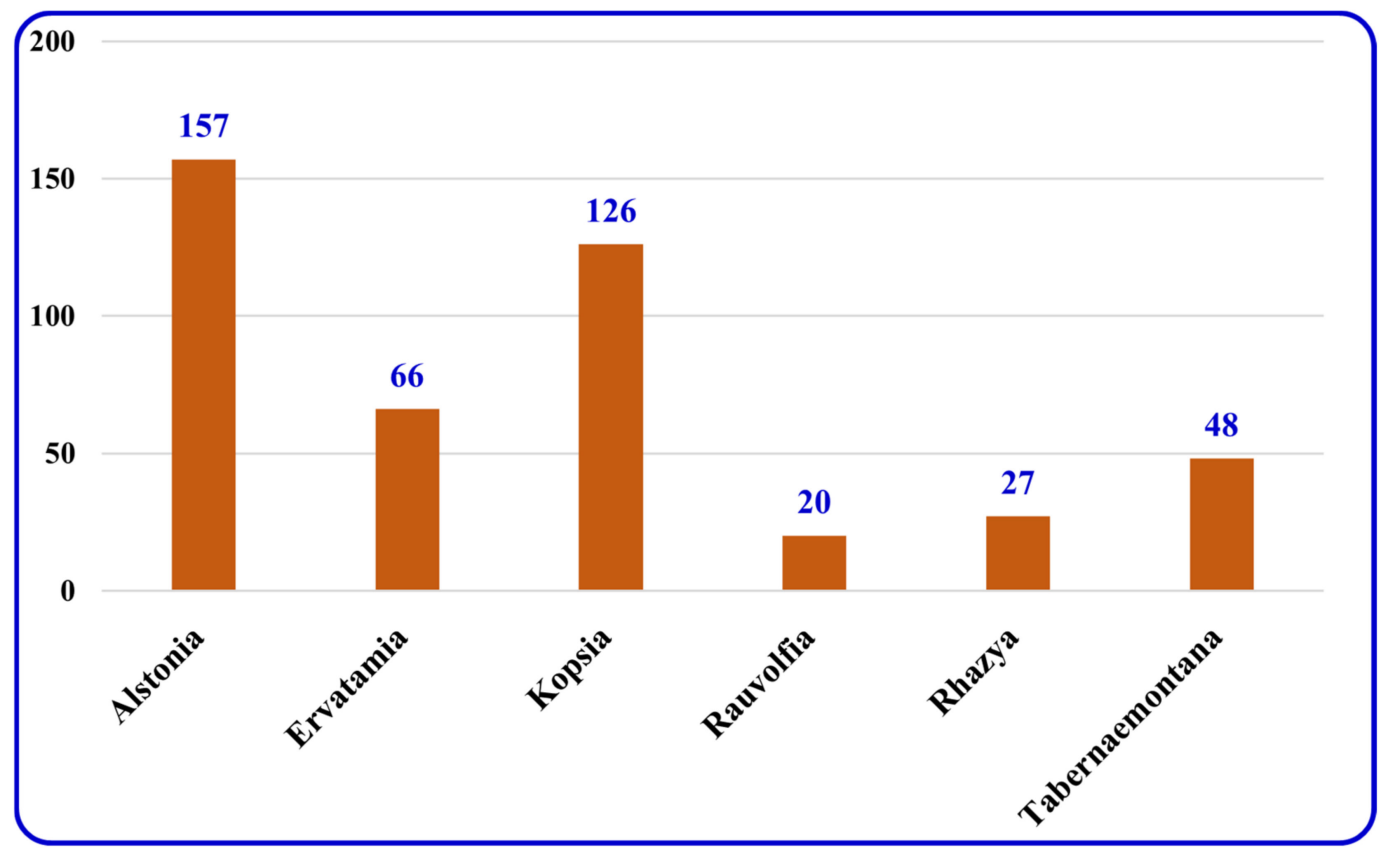
Number of compounds isolated from the six genera.

**Figure 35:**
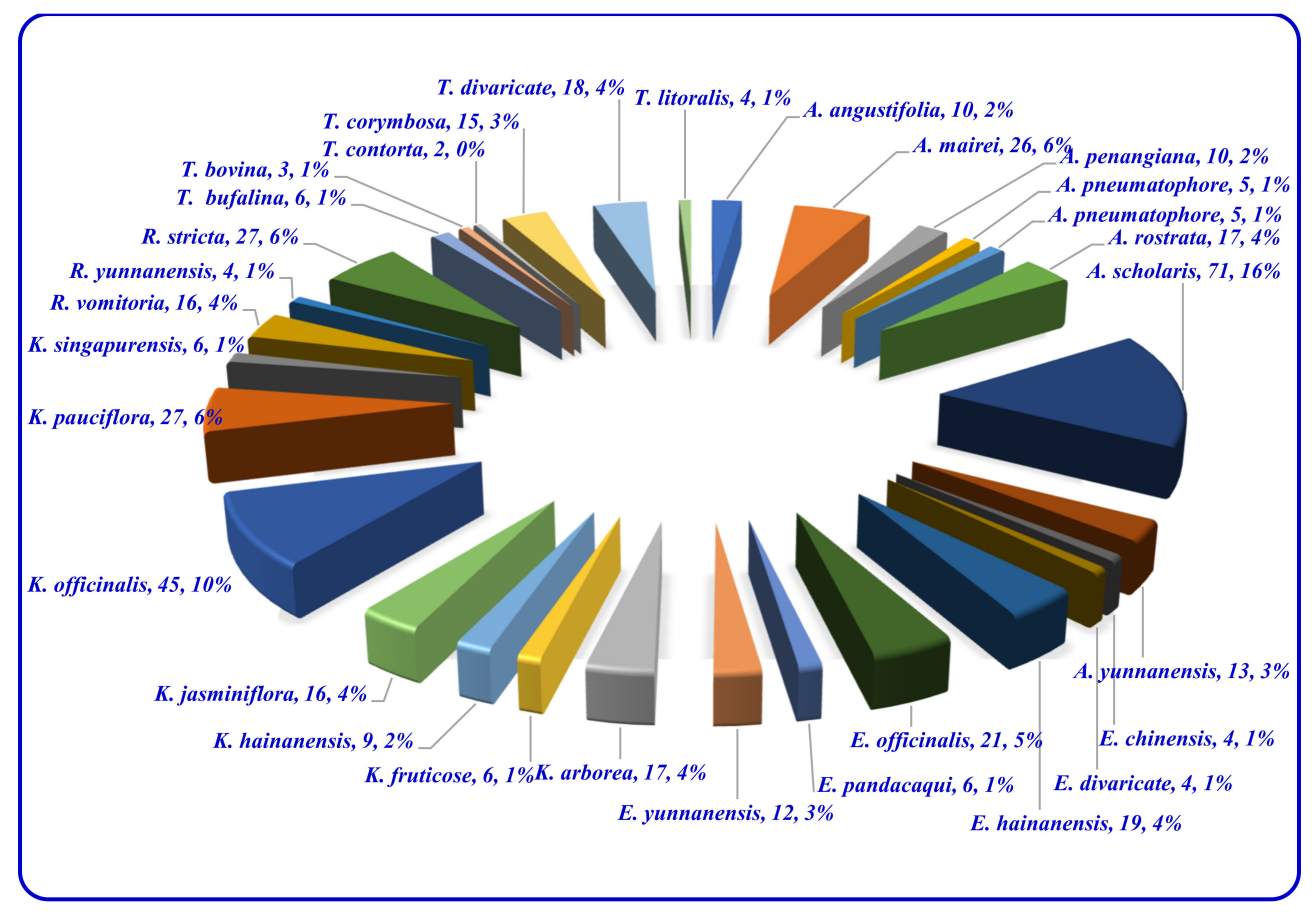
Percentage of reported compounds from the species.

Figure 34 illustrates the number of compounds isolated from the six species; there are 157 (35.4%), 126 (28.4%), 66 (14.9%), 48 (10.8%), 27 (6.1%), and 20 (4.4 %), from *Alstonia*, *Kopsia*, *Ervatamia*, *Tabernaemontana*, *Rhazya* and *Rauvolfia*, respectively. We believe that the six genera are interesting candidate for further investigation. This record coincided with the data illustrated in Figure 35. For example, *Alstonia scholaris* is a species that belongs to the genus *Alstonia* that has produced the highest number of MIAs (71 compounds) and represents 45.2 % of the MITs identified from the same genus between 2010 and 2020. The second and third most interesting species are *Kopsia officinalis* and *Kopsia pauciflora* which produced 45 and 27 compounds, respectively. These two species represent 35.7% and 21.4% of the total compounds produced from the genus *Kopsia*. The fourth most interesting species belong to the genus *Alstonia* (*Alstonia mairei*), which produced 26 compounds and represents 16.5 % of the MITs identified from the genus Alstonia. 

**Figure 36:**
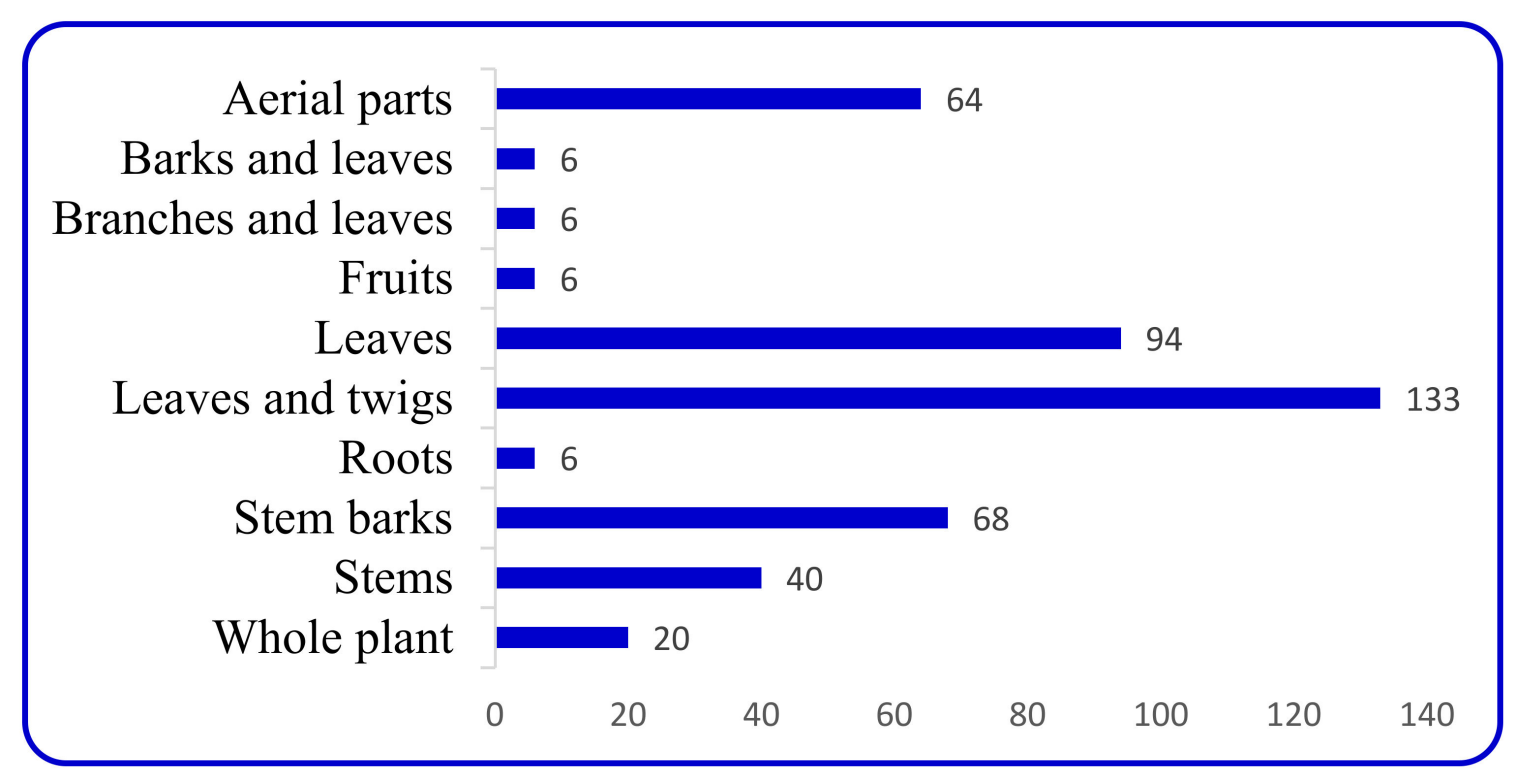
Number of compounds identified from different organs.

It is interesting that the majority of compounds were isolated from twigs and leaves as illustrated in Figure 36. Additionally, the majority of the examined species belonging to the selected six genera were Chinese species and led to the identification of 360 compounds. 

**Figure 37:**
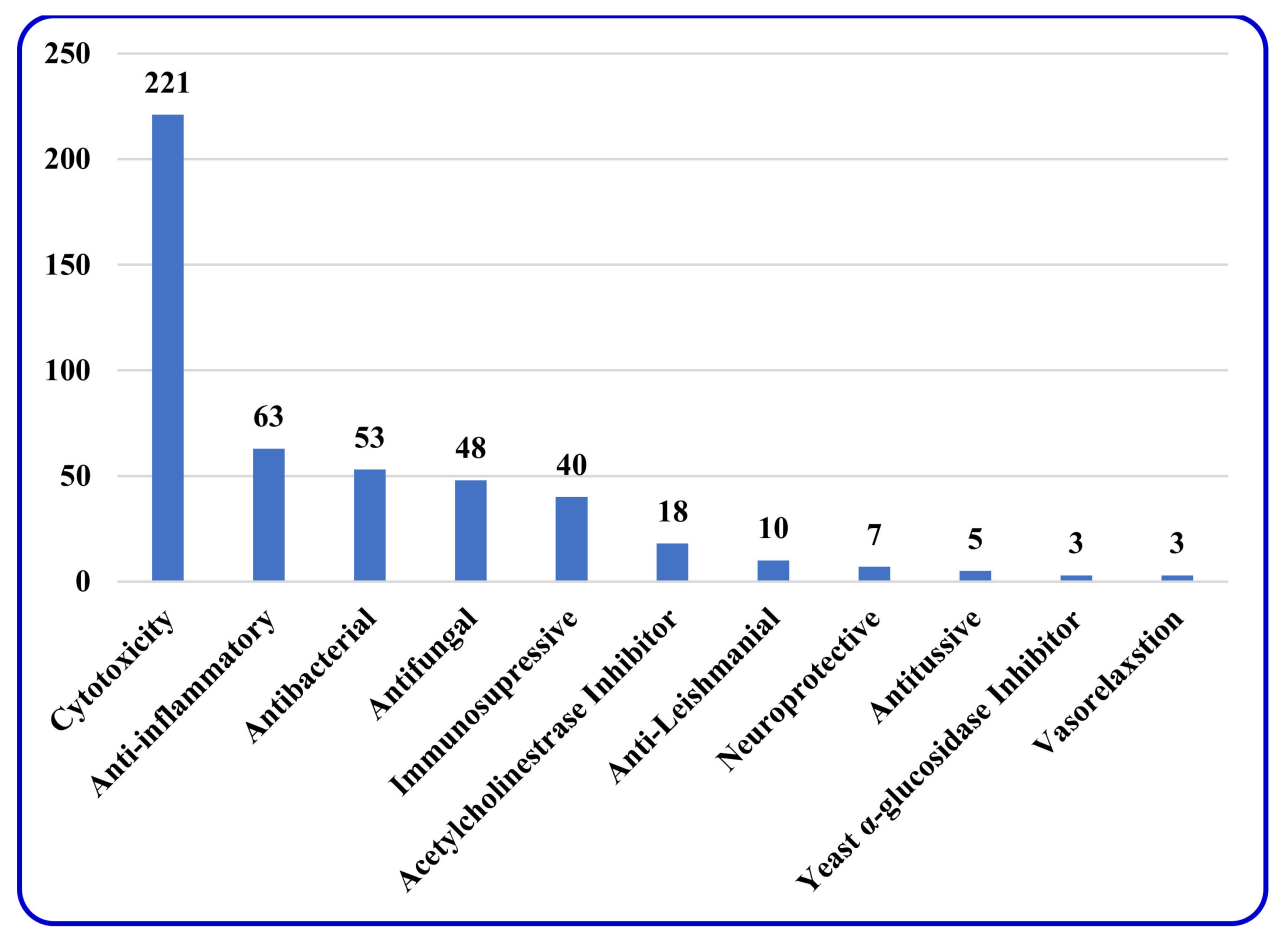
Number of compounds versus biological activities.

Figure 37 presents the biological activities of the compounds. The prominent activity was cytotoxicity followed by anti-inflammatory and antimicrobial activities. Thus, these compounds could be a source of anticancer drugs. 

The family of terpene indole alkaloids has been discovered for over a century. There are numbers of total syntheses studies of these intricate scaffolds have been achieved. Additionally, several reviews and book chapters, as well as the references therein, are interested in the synthetic efforts have been reported.

## Figures and Tables

**Table 1:** Monoterpenoid indole alkaloids from the six species of Apocynaceae.

Comp No	Compound Name	Class Type	Source	Part	Country	Activities
**1**	(14a,15a)-14,15-Epoxy Aspidofractinine	Aspidofractinine	*Alstonia mairei*	Leaves and twigs	China	Cytotoxicity
**2**	Maireine A	Aspidosperma	*Alstonia mairei*	Leaves and twigs	China	Cytotoxicity
**3**	Maireine B	Aspidosperma	*Alstonia mairei*	Leaves and twigs	China	Cytotoxicity
**4**	Venalstonine	Aspidofractinine	*Alstonia mairei*	Leaves and twigs	China	Cytotoxicity
**5**	(−)-Minovincinine	Aspidosperma	*Alstonia mairei*	Leaves and twigs	China	Cytotoxicity
**6**	(−)-11-Methoxymino Vincinine	Aspidosperma	*Alstonia mairei*	Leaves and twigs	China	Cytotoxicity
**7**	(−)-Echitovenine	Aspidosperma	*Alstonia mairei*	Leaves and twigs	China	Cytotoxicity
**8**	Echitovenaldine	Aspidosperma	*Alstonia mairei*	Leaves and twigs	China	Cytotoxicity
**9**	Echitovenidine	Aspidosperma	*Alstonia mairei*	Leaves and twigs	China	Cytotoxicity
**10**	11-Methoxyechitovenidine	Aspidosperma	*Alstonia mairei*	Leaves and twigs	China	Cytotoxicity
**11**	Echitoveniline	Aspidosperma	*Alstonia mairei*	Leaves and twigs	China	Cytotoxicity
**12**	11-Methoxyechitoveniline	Aspidosperma	*Alstonia mairei*	Leaves and twigs	China	Cytotoxicity
**13**	Echitoserpidine	Aspidosperma	*Alstonia mairei*	Leaves and twigs	China	Cytotoxicity
**14**	11-Methoxyechitoserpidine	Aspidosperma	*Alstonia mairei*	Leaves and twigs	China	Cytotoxicity
**15**	Vindolinine	Aspidosperma	*Alstonia mairei*	Leaves and twigs	China	Cytotoxicity
**16**	Lochnericine	Aspidosperma	*Alstonia mairei*	Leaves and twigs	China	Cytotoxicity
**17**	Tabersonine	Aspidosperma	*Alstonia mairei*	Leaves and twigs	China	Cytotoxicity
**18**	Perakine	Ajmaline	*Alstonia mairei*	Leaves and twigs	China	Cytotoxicity
**19**	Picrinine	Picrinine	*Alstonia mairei*	Leaves and twigs	China	Cytotoxicity
**20**	Deacetylpicraline 3,4,5-Trimethoxybenzoate	picraline	*Alstonia mairei*	Leaves and twigs	China	Cytotoxicity
**21**	Picralinal	picraline	*Alstonia mairei*	Leaves and twigs	China	Cytotoxicity
**22**	Rhazimol	Akummidine	*Alstonia mairei*	Leaves and twigs	China	Cytotoxicity
**23**	Alsmaphorazines A	Scholaricine	*Alstonia pneumatophore*	Leaves	Malysia	Anti-inflammatory
**24**	Alsmaphorazine B	Scholaricine	*Alstonia pneumatophore*	Leaves	Malysia	Anti-inflammatory
**25**	Alstrostine A	Strictosidine	*Alstonia rostrata*	Leaves and twigs	China	Cytotoxicity
**26**	Alstrostine B	Strictosidine	*Alstonia rostrata*	Leaves and twigs	China	Cytotoxicity
**27**	Alstrostine C	Akummicine	*Alstonia rostrata*	Leaves and twigs	China	Cytotoxicity
**28**	Alstrostine D	Akummicine	*Alstonia rostrata*	Leaves and twigs	China	Cytotoxicity
**29**	Alstrostine E	Akummicine	*Alstonia rostrata*	Leaves and twigs	China	Cytotoxicity
**30**	Alstrostine F	Corynanthe	*Alstonia rostrata*	Leaves and twigs	China	Cytotoxicity
**31**	11-Hydroxy-6,7-Epoxy-8-Oxo-Vincadifformine	Aspidosperma	*Alstonia yunnanensis*	Whole plant	China	Cytotoxicity
**32**	14-Chloro-15-Hydroxyvinca Difformine	Aspidosperma	*Alstonia yunnanensis*	Whole plant	China	Cytotoxicity
**33**	Perakine *N*_4_-Oxide	Ajmaline	*Alstonia yunnanensis*	Whole plant	China	Cytotoxicity
**34**	Raucaffrinoline *N*_4_-Oxide	Ajmaline	*Alstonia yunnanensis*	Whole plant	China	Cytotoxicity
**35**	Vinorine *N*_1_,*N*_4_-Dioxide	Ajmaline	*Alstonia yunnanensis*	Whole plant	China	Cytotoxicity
**36**	Oxovincadifformine	Aspidosperma	*Alstonia yunnanensis*	Whole plant	China	Cytotoxicity
**37**	Vinorine *N*_4_-Oxide	Ajmaline	*Alstonia yunnanensis*	Whole plant	China	Cytotoxicity
**38**	Vinorine	Ajmaline	*Alstonia yunnanensis*	Whole plant	China	Cytotoxicity
**39**	Alsmaphorazine C	Octahydropyrrolo[2,3-b]pyrrole and 2-azabicyclo[3.3.1]nonane units	*Alstonia pneumatophore*	Leaves	Malaysia	Cytotoxicity
**40**	Alsmaphorazine D	Octahydropyrrolo[2,3-b]pyrrole and 2,8-diazabicyclo[3.3.1]nonane units	*Alstonia pneumatophore*	Leaves	Malaysia	Cytotoxicity
**41**	Alsmaphorazine E	Octahydropyrrolo[2,3-b]pyrrole and 2,8-diazabicyclo[3.3.1]nonane units	*Alstonia pneumatophore*	Leaves	Malaysia	Cytotoxicity
**42**	Scholarisin I	picraline	*Alstonia scholaris*	Leaves	China	Cytotoxicity, Anti-inflammatory Antifungal
**43**	Scholarisin II	picraline	*Alstonia scholaris*	Leaves	China	Cytotoxicity, Anti-inflammatory Antifungal
**44**	Scholarisin III	picraline	*Alstonia scholaris*	Leaves	China	Cytotoxicity, Anti-inflammatory, Antifungal
**45**	Scholarisin IV	picraline	*Alstonia scholaris*	Leaves	China	Cytotoxicity, Anti-inflammatory, Antifungal
**46**	Scholarisin V	picraline	*Alstonia scholaris*	Leaves	China	Cytotoxicity, Anti-inflammatory, Antifungal
**47**	Scholarisin VI	picraline	*Alstonia scholaris*	Leaves	China	Cytotoxicity, Anti-inflammatory, Antifungal
**48**	Scholarisin VII	picraline	*Alstonia scholaris*	Leaves	China	Cytotoxicity, Anti-inflammatory, Antifungal
**49**	(3*R*,5*S*,7*R*,15*R*,16*R*,19*E*)-Scholarisine F	picrinine	*Alstonia scholaris*	Leaves	China	Cytotoxicity, Anti-inflammatory, Antifungal
**50**	3-*Epi*-Dihydrocorymine	Vincorine	*Alstonia scholaris*	Leaves	China	Cytotoxicity, Anti-inflammatory, Antifungal
**51**	(*E*)-16-Formyl-5α-Methoxystrictamine	picraline	*Alstonia scholaris*	Leaves	China	Cytotoxicity, Anti-inflammatory, Antifungal
**52**	Alstolactine A	Corynanthe	*Alstonia scholaris*	Leaves	China	Antibacterial
**53**	Alstolactine B	Corynanthe	*Alstonia scholaris*	Leaves	China	Antibacterial
**54**	Alstolactine C	Corynanthe	*Alstonia scholaris*	Leaves	China	Antibacterial
**55**	Alistonitrine A	Corynanthe	*Alstonia scholaris*	Leaves	China	Anti-inflammatory
**56**	6,7-Epoxy-8-Oxo-Vincadifformine	Aspidosperma	*Alstonia rupestris*	Aerial parts	China	Cytotoxicity, Antifungal, Antibacterial
**57**	11-Acetyl-6,7-Epoxy-8-Oxo-Vincadifformine	Aspidosperma	*Alstonia rupestris*	Aerial parts	China	Cytotoxicity, Antifungal, Antibacterial
**58**	11-Hydroxy-14-Chloro-15-Hydroxyvincadifformine	Aspidosperma	*Alstonia rupestris*	Aerial parts	China	Cytotoxicity, Antifungal, Antibacterial
**59**	Perakine *N*_1_,*N*_4_-Dioxide	Ajmaline	*Alstonia rupestris*	Aerial parts	China	Cytotoxicity, Antifungal, Antibacterial
**60**	11-Hydroxy-6,7-Epoxy-8-Oxovincadifformine	Aspidosperma	*Alstonia rupestris*	Aerial parts	China	Cytotoxicity, Antifungal, Antibacterial
**61**	*N*_(4)_-Methyl-Talpinine	Sarpagine	*Alstonia angustifolia*	Stem bark	Vietnam	Anti-inflammatory, Anti-Leishmanial
**62**	*N*_(4)_-Meth-Yl-*N*_(4)_,21-*Seco*talpinine	Macroline	*Alstonia angustifolia*	Stem bark	Vietnam	Anti-inflammatory, Anti-Leishmanial
**63**	Alstonerinal	Macroline	*Alstonia angustifolia*	Stem bark	Vietnam	Anti-inflammatory, Anti-Leishmanial
**64**	Alstonerine	Macroline	*Alstonia angustifolia*	Stem bark	Vietnam	Anti-inflammatory, Anti-Leishmanial
**65**	Macrocarpine B	Macroline	*Alstonia angustifolia*	Stem bark	Vietnam	Anti-inflammatory, Anti-Leishmanial
**66**	Affinisine	Sarpagine	*Alstonia angustifolia*	Stem bark	Vietnam	Anti-inflammatory, Anti-Leishmanial
**67**	Villalstonine	Macroline-Pleiocarpamine	*Alstonia angustifolia*	Stem bark	Vietnam	Anti-inflammatory, Anti-Leishmanial
**68**	Villalstonine *N*_(4)_-Oxide	Macroline-Pleiocarpamine	*Alstonia angustifolia*	Stem bark	Vietnam	Anti-inflammatory, Anti-Leishmanial
**69**	Villalstonidine D	Macroline-Pleiocarpamine	*Alstonia angustifolia*	Stem bark	Vietnam	Anti-inflammatory, Anti-Leishmanial
**70**	Villalstonidine E	Macroline-Pleiocarpamine	*Alstonia angustifolia*	Stem bark	Vietnam	Anti-inflammatory, Anti-Leishmanial
**71**	Normavacurine-21-One	Pleiocarpaman	*Alstonia scholaris*	Leaves	China	Antibacterial
**72**	5-Hydroxy-19,20-*E*-Alschomine	Corynanthe	*Alstonia scholaris*	Leaves	China	Antibacterial
**73**	5-Hydroxy-19,20-Z-Alschomine	Corynanthe	*Alstonia scholaris*	Leaves	China	Antibacterial
**74**	Alstoniascholarine A	Corynanthe	*Alstonia scholaris*	Leaves	China	Antibacterial, Anti- Fungal
**75**	Alstoniascholarine B	Corynanthe	*Alstonia scholaris*	Leaves	China	Antibacterial, Anti- Fungal
**76**	Alstoniascholarine C	Corynanthe	*Alstonia scholaris*	Leaves	China	Antibacterial, Anti- Fungal
**77**	Alstoniascholarine D	Corynanthe	*Alstonia scholaris*	Leaves	China	Antibacterial, Anti- Fungal
**78**	Alstoniascholarine E	Corynanthe	*Alstonia scholaris*	Leaves	China	Antibacterial, Anti- Fungal
**79**	Alstoniascholarine F	Scholarisine	*Alstonia scholaris*	Leaves	China	Antibacterial, Anti- Fungal
**80**	Alstoniascholarine G	Scholarisine	*Alstonia scholaris*	Leaves	China	Antibacterial, Anti- Fungal
**81**	Alstoniascholarine H	Scholarisine	*Alstonia scholaris*	Leaves	China	Antibacterial, Anti- Fungal
**82**	Alstoniascholarine I	Scholarisine	*Alstonia scholaris*	Leaves	China	Antibacterial, Anti- Fungal
**83**	Alstoniascholarine J	Scholarisine	*Alstonia scholaris*	Leaves	China	Antibacterial, Anti- Fungal
**84**	Alstoniascholarine K	Scholarisine	*Alstonia scholaris*	Leaves	China	Antibacterial, Anti- Fungal
**85**	Alstoniascholarine L	Corynanthe	*Alstonia scholaris*	Leaves	China	Cytotoxicity
**86**	Alstoniascholarine M	Corynanthe	*Alstonia scholaris*	Leaves	China	Cytotoxicity
**87**	Alstoniascholarine N	Corynanthe	*Alstonia scholaris*	Leaves	China	Cytotoxicity
**88**	Alstoniascholarine O	Scholarisine	*Alstonia scholaris*	Leaves	China	Cytotoxicity
**89**	Alstoniascholarine P	Scholarisine	*Alstonia scholaris*	Leaves	China	Cytotoxicity
**90**	Alstoniascholarine Q	Scholarisine	*Alstonia scholaris*	Leaves	China	Cytotoxicity
**91**	Scholarisine H	Corynanthe	*Alstonia scholaris*	Leaves	China	Antibacterial
**92**	Scholarisine I	Nareline	*Alstonia scholaris*	Leaves	China	Antibacterial
**93**	Scholarisine J	Nareline	*Alstonia scholaris*	Leaves	China	Antibacterial
**94**	Scholarisine K	Corynanthe	*Alstonia scholaris*	Leaves	China	Antibacterial
**95**	Scholarisine L	Corynanthe	*Alstonia scholaris*	Leaves	China	Antibacterial
**96**	Scholarisine M	Corynanthe	*Alstonia scholaris*	Leaves	China	Antibacterial
**97**	Scholarisine N	Corynanthe	*Alstonia scholaris*	Leaves	China	Antibacterial
**98**	Melosline A	Corynanthe	*Alstonia scholaris*	Leaves and twigs	China	Cytotoxicity
**99**	Melosline B	Secodine	*Alstonia scholaris*	Leaves and twigs	China	Cytotoxicity
**100**	1-[2-[2-(Carboxymethyl) Indole-3-Yl] Ethyl]-3-Ethylpyridinium Hydroxide Inner Salt	Secodine	*Alstonia scholaris*	Leaves and twigs	China	Cytotoxicity
**101**	Alstiyunnanenine A	Sarpagine	*Alstonia Yunnanensis*	Aerial parts	China	Cytotoxicity
**102**	Alstiyunnanenine B	Picraline	*Alstonia Yunnanensis*	Aerial parts	China	Cytotoxicity
**103**	Alstiyunnanenine C	Akummiline	*Alstonia Yunnanensis*	Aerial parts	China	Cytotoxicity
**104**	Alstiyunnanenine D	Scholaricine	*Alstonia Yunnanensis*	Aerial parts	China	Cytotoxicity
**105**	Alstiyunnanenine E	Scholaricine	*Alstonia Yunnanensis*	Aerial parts	China	Cytotoxicity
**106**	Alstomairine A	Scholaricine	*Alstonia Mairei*	Leaves	China	Cytotoxicity
**107**	Alstomairine B	Scholaricine	*Alstonia Mairei*	Leaves	China	Cytotoxicity
**108**	Alstomairine C	Scholaricine	*Alstonia Mairei*	Leaves	China	Cytotoxicity
**109**	Alpneumine A	Scholaricine	*Alstonia Mairei*	Leaves	China	Cytotoxicity
**110**	Alstrostine G	Corynanthe	*Alstonia rostrata*	Bark and trunks	China	Cytotoxicity
**111**	Alstrostine H	Corynanthe	*Alstonia rostrata*	Bark and trunks	China	Cytotoxicity
**112**	Alstrostine I	Scholarisine	*Alstonia rostrata*	Bark and trunks	China	Cytotoxicity
**113**	Alstrostine J	Secodine	*Alstonia rostrata*	Bark and trunks	China	Cytotoxicity
**114**	Alstrostine K	Corynanthe	*Alstonia rostrata*	Bark and trunks	China	Cytotoxicity
**115**	Scholarisine T	Nareline	*Alstonia scholaris*	Leaves	China	Antibacterial
**116**	Scholarisine U	Nareline	*Alstonia scholaris*	Leaves	China	Antibacterial
**117**	Scholarisine V	Nareline	*Alstonia scholaris*	Leaves	China	Antibacterial
**118**	Scholarisine W	Nareline	*Alstonia scholaris*	Leaves	China	Antibacterial
**119**	Scholarisine A	Nareline	*Alstonia scholaris*	Leaves	China	Antibacterial
**120**	Scholarisine P	Nareline	*Alstonia scholaris*	Leaves	China	Anti-inflammatory
**121**	Scholarisine Q	Akuammiline	*Alstonia scholaris*	Leaves	China	Anti-inflammatory
**122**	Scholarisine R	Corynanthe	*Alstonia scholaris*	Leaves	China	Anti-inflammatory
**123**	Scholarisine S	Nareline	*Alstonia scholaris*	Leaves	China	Anti-inflammatory
**124**	(16*R*)-*E*-Isositsnikine	Corynanthe	*Alstonia scholaris*	Leaves	China	Anti-inflammatory
**125**	Nareline	Nareline	*Alstonia scholaris*	Leaves	China	Anti-inflammatory
**126**	5-Methoxystrictamine	Akuammiline	*Alstonia scholaris*	Leaves	China	Anti-inflammatory
**127**	Leuconolam	Aspidosperma	*Alstonia scholaris*	Leaves	China	Anti-inflammatory
**128**	Epileuconolam	Aspidosperma	*Alstonia scholaris*	Leaves	China	Anti-inflammatory
**129**	*N*_b_-Demethylalstogustine	Scholarisine	*Alstonia scholaris*	Leaves	China	Anti-inflammatory
**130**	19-Epischolaricine	Scholarisine	*Alstonia scholaris*	Leaves	China	Anti-inflammatory
**131**	Scholaricine	Scholarisine	*Alstonia scholaris*	Leaves	China	Anti-inflammatory
**132**	Vallesamine	Corynanthe	*Alstonia scholaris*	Leaves	China	Anti-inflammatory
**133**	Akuammidine	Akuammidine	*Alstonia scholaris*	Leaves	China	Anti-inflammatory
**134**	17-*Nor*-Excelsinidine	Corynanthe	*Alstonia scholaris*	Leaves	China	Anti-inflammatory
**135**	Strictosamide	Corynanthe	*Alstonia scholaris*	Leaves	China	Anti-inflammatory
**136**	Vincamaginine A	Ajmaline	*Alstonia penangiana*	Leaves	Malaysia	Cytotoxicity
**137**	Vincamaginine B	Ajmaline	*Alstonia penangiana*	Leaves	Malaysia	Cytotoxicity
**138**	Alstonisinine A	Macroline Oxindole	*Alstonia penangiana*	Leaves	Malaysia	Cytotoxicity
**139**	Alstonisinine B	Macroline Oxindole	*Alstonia penangiana*	Leaves	Malaysia	Cytotoxicity
**140**	Alstonisinine C	Macroline Oxindole	*Alstonia penangiana*	Leaves	Malaysia	Cytotoxicity
**141**	Alstonoxine F	Macroline Oxindole	*Alstonia penangiana*	Leaves	Malaysia	Cytotoxicity
**142**	Angustilongine A	Macroline-Akuammiline	*Alstonia penangiana*	Leaves	Malaysia	Cytotoxicity
**143**	Angustilongine B	Macroline-Akuammiline	*Alstonia penangiana*	Leaves	Malaysia	Cytotoxicity
**144**	Angustilongine C	Macroline-Akuammiline	*Alstonia penangiana*	Leaves	Malaysia	Cytotoxicity
**145**	Angustilongine D	Macroline-Akuammiline	*Alstonia penangiana*	Leaves	Malaysia	Cytotoxicity
**146**	Winphyllines A	Vincorine	*Alstonia rostrata*	Twigs	China	Cytotoxicity
**147**	Winphyllines B	Scholarisine	*Alstonia rostrata*	Twigs	China	Cytotoxicity
**148**	*N*_b_-Demethylechitamine	Vincorine	*Alstonia rostrata*	Twigs	China	Cytotoxicity
**149**	17-*O*-Acetylnorechitamine	Vincorine	*Alstonia rostrata*	Twigs	China	Cytotoxicity
**150**	12- Methoxyechitamidine	Scholarisine	*Alstonia rostrata*	Twigs	China	Cytotoxicity
**151**	*N*_(4)_-Demethylastogustine	Scholarisine	*Alstonia rostrata*	Twigs	China	Cytotoxicity
**152**	17-Formyl-10-Demethoxyvincorine *N*_(4)_-Oxide	Vincorine	*Alstonia scholaris*	Leaves	China	_
**153**	10-Methoxyalstiphyllanine H	Ajmaline	*Alstonia scholaris*	Leaves	China	_
**154**	10-Demethoxyvincorine *N*_(4)_-Oxide	Vincorine	*Alstonia scholaris*	Leaves	China	_
**155**	Alstoscholactine	Corynanthe	*Alstonia scholaris*	Leaves	Malaysia	Vasorelaxation Cytotoxicity
**156**	Alstolaxepine	Corynanthe	*Alstonia scholaris*	Leaves	Malaysia	Vasorelaxation Cytotoxicity
**157**	Alstobrogaline	Corynanthe	*Alstonia scholaris*	Leaves	Malaysia	Cytotoxicity
**158**	Kopsiyunnanines G	Aspidosperma	*Kopsia arbora*	Aerial parts	China	_
**159**	Kopsiyunnanines H	Aspidosperma	*Kopsia arbora*	Aerial parts	China	_
**160**	Kopsihainin A	Aspidosperma	*Kopsia hainanensis*	Stems	China	Antitussive
**161**	Kopsihainin B	Aspidofractinine	*Kopsia hainanensis*	Stems	China	Antitussive
**162**	Kopsihainin C	Aspidofractinine	*Kopsia hainanensis*	Stems	China	Antitussive
**163**	Kopsinine	Aspidofractinine	*Kopsia hainanensis*	Stems	China	Antitussive
**164**	Methyl Demethoxycarbonylchanofruticosinate	Methyl Chanofruticosinate	*Kopsia hainanensis*	Stems	China	Antitussive
**165**	Singaporentine A	Aspidofractinine	*Kopsia singapurensis*	Barks and leaves	Malaysia	_
**166**	*N*_(1)_-Formylkopsininic Acid	Aspidofractinine	*Kopsia singapurensis*	Barks and leaves	Malaysia	_
**167**	*N*_(1)_-Formylkopsininic Acid-*N*_(4)_-Oxide	Aspidofractinine	*Kopsia singapurensis*	Barks and leaves	Malaysia	_
**168**	15-Hydroxykopsamine	Aspidofractinine	*Kopsia singapurensis*	Barks and leaves	Malaysia	_
**169**	14α-Hydroxy-*N*_(4)_-Methylcondylocarpine	Aspidosoermata	*Kopsia singapurensis*	Barks and leaves	Malaysia	_
**170**	Singaporentinidine	Corynanthe	*Kopsia singapurensis*	Barks and leaves	Malaysia	_
**171**	Kopsininate	Aspidofractinie	*Kopsia hainanensis*	Leaves and stems	China	Antifungal, Antibacterial
**172**	*N*_1_-Decarbomethoxy Chanofruticosinic Acid	Methyl Chanofruticosinate	*Kopsia hainanensis*	Leaves and stems	China	Antifungal, Antibacterial
**173**	Methyl *N*_1_- Decarbomethoxy Chanofruticosinate *N*_(4)_-Oxide	Methyl Chanofruticosinate	*Kopsia hainanensis*	Leaves and stems	China	Antifungal, Antibacterial
**174**	Methyl Chanofruticosinate *N*_(4)_-Oxide	Methyl Chanofruticosinate	*Kopsia hainanensis*	Leaves and stems	China	Antifungal, Antibacterial
**175**	5,6-Secokopsinine	Aspidofractinine	*Kopsia jasminiflora*	Stem barks	Thailand	Cytotoxicity
**176**	5β-Hydroxykopsinine	Aspidofractinine	*Kopsia jasminiflora*	Stem barks	Thailand	Cytotoxicity
**177**	16-*Epi*-Kopsinilam	Aspidofractinine	*Kopsia jasminiflora*	Stem barks	Thailand	Cytotoxicity
**178**	5-Oxokopsinic Acid	Aspidofractinine	*Kopsia jasminiflora*	Stem barks	Thailand	Cytotoxicity
**179**	*N*_a_-Demethoxycarbonyl-12-Methoxykopsine	Kopsine	*Kopsia jasminiflora*	Stem barks	Thailand	Cytotoxicity
**180**	14(*S*)-Hydroxy-19(*R*)- Methoxytubotaiwine	Strychnos	*Kopsia jasminiflora*	Stem barks	Thailand	Cytotoxicity
**181**	19-Oxo-(−)-Eburnamonine	Vincamine	*Kopsia jasminiflora*	Stem barks	Thailand	Cytotoxicity
**182**	19(*S*)-Hydroxy-Δ^14^-Vincamone	Vincamine	*Kopsia jasminiflora*	Stem barks	Thailand	Cytotoxicity
**183**	Kopsinilam	Aspidofractinine	*Kopsia jasminiflora*	Stem barks	Thailand	Cytotoxicity
**184**	Kopsinic Acid	Aspidofractinine	*Kopsia jasminiflora*	Stem barks	Thailand	Cytotoxicity
**185**	12-Methoxykopsine	Kopsine	*Kopsia jasminiflora*	Stem barks	Thailand	Cytotoxicity
**186**	Kopsanone	Kopsine	*Kopsia jasminiflora*	Stem barks	Thailand	Cytotoxicity
**187**	19(*R*)- Methoxytubotaiwine	Strychnos	*Kopsia jasminiflora*	Stem barks	Thailand	Cytotoxicity
**188**	(−)-Eburnamonine	Vincamine	*Kopsia jasminiflora*	Stem barks	Thailand	Cytotoxicity
**189**	19-OH-(−)-Eburnamonine	Vincamine	*Kopsia jasminiflora*	Stem barks	Thailand	Cytotoxicity, Acetylcholinesterase inhibitor
**190**	Δ^14^-Vincamone	Vincamine	*Kopsia jasminiflora*	Stem barks	Thailand	Cytotoxicity
**191**	Phutdonginin	Eburnane	*Kopsia arborea*	Twigs	Thailand	Antibacterial, Acetylcholinesterase inhibition
**192**	Melodinine E	Aspidosperma	*Kopsia arborea*	Twigs	Thailand	Antibacterial, Acetylcholinesterase inhibition
**193**	Kopsilongine	Aspidofractinine	*Kopsia arborea*	Twigs	Thailand	Antibacterial, Acetylcholinesterase inhibition
**194**	Kopsamine	Aspidofractinine	*Kopsia arborea*	Twigs	Thailand	Antibacterial, Acetylcholinesterase inhibition
**195**	(−)-Methylenedioxy-11,12-Kopsinaline	Aspidofractinine	*Kopsia arborea*	Twigs	Thailand	Antibacterial, Acetylcholinesterase inhibition
**196**	Decarbomethoxykopsiline	Kopsine	*Kopsia arborea*	Twigs	Thailand	Antibacterial, Acetylcholinesterase inhibition
**197**	Vincadifformine	Aspidosperma	*Kopsia arborea*	Twigs	Thailand	Antibacterial, Acetylcholinesterase inhibition
**198**	Arboridinine	Corynanthe	*Kopsia arborea*	_	Malaysia	Relaxation Effect
**199**	Kopsiyunnanines J1 and J2	Aspidosoermata	*Kopsia arborea*	Aerial parts	China	_
**200**	Paucidirinine	Aspidofractinine	*Kopsia pauciflora*	Stem bark	Malaysia	Cytotoxicity
**201**	Paucidirisine	Aspidofractinine	*Kopsia pauciflora*	Stem bark	Malaysia	Cytotoxicity
**202**	Paucidactinine	Aspidosperma	*Kopsia pauciflora*	Stem bark	Malaysia	Cytotoxicity
**203**	Pauciduridine	Aspidofractinine	*Kopsia pauciflora*	Stem bark	Malaysia	Cytotoxicity
**204**	Paucidactine D	Paucidactine	*Kopsia pauciflora*	Stem bark	Malaysia	Cytotoxicity
**205**	Paucidactine E	Paucidactine	*Kopsia pauciflora*	Stem bark	Malaysia	Cytotoxicity
**206**	Paucidisine	Kopsine	*Kopsia pauciflora*	Stem bark	Malaysia	Cytotoxicity
**207**	(−)-19-Oxoisoeburnamine	Eburnane	*Kopsia pauciflora*	Stem bark	Malaysia	Cytotoxicity
**208**	(−)-19(*R*)-Hydroxyeburnamenine	Eburnane	*Kopsia pauciflora*	Stem bark	Malaysia	Cytotoxicity
**209**	(−)-19(*R*)-Hydroxy-*O*-Ethylisoeburnamine	Eburnane	*Kopsia pauciflora*	Stem bark	Malaysia	Cytotoxicity
**210**	Larutienine B	Eburnane	*Kopsia pauciflora*	Stem bark	Malaysia	Cytotoxicity
**211**	Paucidactine A	Paucidactine	*Kopsia pauciflora*	Stem bark	Malaysia	Cytotoxicity
**212**	Paucidactine B	Paucidactine	*Kopsia pauciflora*	Stem bark	Malaysia	Cytotoxicity
**213**	Paucidactine C	Paucidactine	*Kopsia pauciflora*	Stem bark	Malaysia	Cytotoxicity
**214**	5, 22-Dioxokopsane	Kopsine	*Kopsia pauciflora*	Stem bark	Malaysia	Cytotoxicity
**215**	(+)-Eburnamonine	Eburnane	*Kopsia pauciflora*	Stem bark	Malaysia	Cytotoxicity,
**216**	Eburnamenine	Eburnane	*Kopsia pauciflora*	Stem bark	Malaysia	Cytotoxicity
**217**	(−)-Eburnamine	Eburnane	*Kopsia pauciflora*	Stem bark	Malaysia	Cytotoxicity
**218**	(+)-Isoeburnamine	Eburnane	*Kopsia pauciflora*	Stem bark	Malaysia	Cytotoxicity
**219**	(+)-19-Oxoeburnamine	Eburnane	*Kopsia pauciflora*	Stem bark	Malaysia	Cytotoxicity
**220**	(−)-19(*R*)-Hydroxyisoeburnamine	Eburnane	*Kopsia pauciflora*	Stem bark	Malaysia	Cytotoxicity
**221**	(+)-19(*R*)-Hydroxyeburnamine	Eburnane	*Kopsia pauciflora*	Stem bark	Malaysia	Cytotoxicity
**222**	Larutienine A	Eburnane	*Kopsia pauciflora*	Stem bark	Malaysia	Cytotoxicity
**223**	(−)-Norpleiomutine	Eburnane- Aspidofractinine	*Kopsia pauciflora*	Stem bark	Malaysia	Cytotoxicity
**224**	(+)-Kopsoffinol	Eburnane- Aspidofractinine	*Kopsia pauciflora*	Stem bark	Malaysia	Cytotoxicity
**225**	(−)-Demethylnorpleiomutine	Eburnane- Aspidofractinine	*Kopsia pauciflora*	Stem bark	Malaysia	Cytotoxicity
**226**	(+)-Kopsoffine	Vincamine- Aspidofractinine	*Kopsia pauciflora*	Stem bark	Malaysia	Cytotoxicity
**227**	Kopsiyunnanine M	Scholarisine- Corynanthe	*Kopsia arborea*	Aerial partss	China	_
**228**	Arborisidine	Pericine	*Kopsia arborea*	Whole plant	Malayan	Cytotoxicity
**229**	Arbornamine	Arbornane	*Kopsia arborea*	Whole plant	Malayan	Cytotoxicity
**230**	Kopsinidine C	Kopsine	*Kopsia officinalis$*	Twigs and leaves	China	Immunosuppressive activity
**231**	Kopsinidine D	Kopsine	*Kopsia officinalis$*	Twigs and leaves	China	Immunosuppressive activity
**232**	Kopsinidine E	Kopsine	*Kopsia officinalis$*	Twigs and leaves	China	Immunosuppressive activity
**233**	11,12-Methylenedioxychanofruticosinic Acid	Methyl Chanofruticosinate	*Kopsia officinalis*	Twigs and leaves	China	Immunosuppressive activity
**234**	12-Methoxychanofruticosinic Acid	Methyl Chanofruticosinate	*Kopsia officinalis*	Twigs and leaves	China	Immunosuppressive activity
**235**	*N*_(4)_-Methylkopsininate	Aspidofractinine	*Kopsia officinalis*	Twigs and leaves	China	Immunosuppressive activity
**236**	Chanofruticosinic Acid	Methyl Chanofruticosinate	*Kopsia officinalis*	Twigs and leaves	China	Immunosuppressive activity
**237**	Kopsinine Methochloride	Aspidofractinine	*Kopsia officinalis*	Twigs and leaves	China	Immunosuppressive activity
**238**	Demethoxycarbonylkopsin	Kopsine	*Kopsia officinalis*	Twigs and leaves	China	Immunosuppressive activity
**239**	Methyl Chanofruticosinate	Methyl Chanofruticosinate	*Kopsia officinalis*	Twigs and leaves	China	Immunosuppressive activity
**240**	Methyl 11,12-Methylenedioxychanofruticosinate	Methyl Chanofruticosinate	*Kopsia officinalis*	Twigs and leaves	China	Immunosuppressive activity
**241**	Methyl 12-Methoxychanofruticosinate	Methyl Chanofruticosinate	*Kopsia officinalis*	Twigs and leaves	China	Immunosuppressive activity
**242**	Methyl 11,12-Methylenedioxy-*N*_1_-Decarbomethoxychanofruticosinate	Methyl Chanofruticosinate	*Kopsia officinalis*	Twigs and leaves	China	Immunosuppressive activity
**243**	Kopsininic Acid	Aspidofractinine	*Kopsia officinalis*	Twigs and leaves	China	Immunosuppressive activity
**244**	(−)-11,12-Methylenedioxykopsinaline	Aspidofractinine	*Kopsia officinalis*	Twigs and leaves	China	Immunosuppressive activity
**245**	(−)-*N*-Methoxycarbonyl-11,12-Methylenedioxykopsinaline	Aspidofractinine	*Kopsia officinalis*	Twigs and leaves	China	Immunosuppressive activity
**246**	(−)-*N*-Methoxycarbonyl- 12-Methoxykopsinaline	Aspidofractinine	*Kopsia officinalis*	Twigs and leaves	China	Immunosuppressive activity
**247**	*N*-Carbomethoxy-11-Hydroxy-12- Methoxykopsinaline	Aspidofractinine	*Kopsia officinalis*	Twigs and leaves	China	Immunosuppressive activity
**248**	Kopsinoline	Aspidofractinine	*Kopsia officinalis*	Twigs and leaves	China	Immunosuppressive activity
**249**	(−)-12-Methoxykopsinaline	Aspidofractinine	*Kopsia officinalis*	Twigs and leaves	China	Immunosuppressive activity
**250**	11,12-Methylenedioxykopsinaline *N*_(4)_- Oxide	Aspidofractinine	*Kopsia officinalis*	Twigs and leaves	China	Immunosuppressive activity
**251**	Kopsinine B	Aspidofractinine	*Kopsia officinalis*	Twigs and leaves	China	Immunosuppressive activity
**252**	Rhazinilam	Aspidosperma	*Kopsia officinalis*	Twigs and leaves	China	Immunosuppressive activity
**253**	Pleiocarpamine Methochloride	Corynanthe	*Kopsia officinalis*	Twigs and leaves	China	Immunosuppressive activity
**254**	Kopsioffine A	Aspidosperma	*Kopsia officinalis*	Leaves and stems	China	Yeast α-glucosidase inhibitory
**255**	Kopsioffine B	Aspidosperma	*Kopsia officinalis*	Leaves and stems	China	Yeast α-glucosidase inhibitory
**256**	Kopsioffine C	Aspidosperma	*Kopsia officinalis*	Leaves and stems	China	Yeast α-glucosidase inhibitory
**257**	Kopsifoline G	Aspidosperma	*Kopsia fruticose*	Aerial parts	China	Cytotoxicity, Antifungal, Antibacterial
**258**	Kopsifoline H	Aspidosperma	*Kopsia fruticose*	Aerial parts	China	Cytotoxicity, Antifungal, Antibacterial
**259**	Kopsifoline I	Aspidosperma	*Kopsia fruticose*	Aerial parts	China	Cytotoxicity, Antifungal, Antibacterial
**260**	Kopsifoline J	Aspidosperma	*Kopsia fruticose*	Aerial parts	China	Cytotoxicity, Antifungal, Antibacterial
**261**	Kopsifoline K	Aspidosperma	*Kopsia fruticose*	Aerial parts	China	Cytotoxicity, Antifungal, Antibacterial
**262**	Kopsifoline A	Aspidosperma	*Kopsia fruticose*	Aerial parts	China	Cytotoxicity, Antifungal, Antibacterial
**263**	Kopsiarborine A	Aspidofractinine	*Kopsia arborea*	Aerial parts	China	Cytotoxicity
**264**	Kopsiarborine B	Methyl Chanofruticosinate	*Kopsia arborea*	Aerial parts	China	Cytotoxicity
**265**	Kopsiarborine C	Aspidosperma	*Kopsia arborea*	Aerial parts	China	Cytotoxicity
**266**	Kopsiaofficine A	Aspidofractinine	*Kopsia officinalis*	Aerial parts	China	Cytotoxicity
**267**	Kopsiaofficine B	Paucidactine	*Kopsia officinalis*	Aerial parts	China	Cytotoxicity
**268**	Kopsiaofficine C	Aspidofractinine	*Kopsia officinalis*	Aerial parts	China	Cytotoxicity
**269**	Kopsiofficine H	Eburnane	*Kopsia officinalis*	Stems	China	Anti-inflammatory
**270**	Kopsiofficine I	Eburnane	*Kopsia officinalis*	Stems	China	Anti-inflammatory
**271**	Kopsiofficine J	Eburnane	*Kopsia officinalis*	Stems	China	Anti-inflammatory
**272**	Kopsiofficine K	Eburnane	*Kopsia officinalis*	Stems	China	Anti-inflammatory
**273**	Kopsiofficine L	Kopsine	*Kopsia officinalis*	Stems	China	Anti-inflammatory
**274**	(+)-*O*-Methyleburnamine	Eburnane	*Kopsia officinalis*	Stems	China	Anti-inflammatory
**275**	(−)-*O*-Methylisoeburnamine	Eburnane	*Kopsia officinalis*	Stems	China	Anti-inflammatory
**276**	16-Isoeburnamine	Eburnane	*Kopsia officinalis*	Stems	China	Anti-inflammatory
**277**	20-Oxoeburnamenine	Eburnane	*Kopsia officinalis*	Stems	China	Anti-inflammatory
**278**	Methyl 11, 12-Methylenedioxychanofruticosinate	Methyl Chanofruticosinate	*Kopsia officinalis*	Stems	China	Anti-inflammatory
**279**	Methyl *N*-(Decarbomethoxy)-11,12-(Methylenedioxy) Chanofruticosinate	Methyl Chanofruticosinate	*Kopsia officinalis*	Stems	China	Anti-inflammatory
**280**	*O*-Methylleuconolam	Aspidosperma	*Kopsia officinalis*	Stems	China	Anti-inflammatory
**281**	Leuconodine D	Aspidosperma	*Kopsia officinalis*	Stems	China	Anti-inflammatory
**282**	Oxayohimban-16-Carboxylic Acid	Corynanthe	*Kopsia officinalis*	Stems	China	Anti-inflammatory
**283**	19, 20-Dihydroisositsirikine	Corynanthe	*Kopsia officinalis*	Stems	China	Anti-inflammatory
**284**	Rauvomine A	Sarpagine	*Rauvolfia vomitoria*	Aerial parts	China	Anti-inflammatory
**285**	Rauvomine B	Sarpagine	*Rauvolfia vomitoria*	Aerial parts	China	Anti-inflammatory
**286**	Peraksine	Sarpagine	*Rauvolfia vomitoria*	Aerial parts	China	Anti-inflammatory
**287**	Alstoyunine A	Sarpagine	*Rauvolfia vomitoria*	Aerial parts	China	Anti-inflammatory
**288**	11-Hydroxyburnamine	Picraline	*Rauvolfia yunnanensis*	Whole plant	China	Cytotoxicity Immunosuppressive
**289**	Rauvoyunnanine A	Sarpagine	*Rauvolfia yunnanensis*	Whole plant	China	Cytotoxicity Immunosuppressive
**290**	Rauvoyunnanine B	Corynanthe	*Rauvolfia yunnanensis*	Whole plant	China	Cytotoxicity Immunosuppressive
**291**	Lochnerine	Sarpagine	*Rauvolfia yunnanensis*	Whole plant	China	Cytotoxicity Immunosuppressive
**292**	Serpentinic Acid	Corynanthe	*Rauvolfia yunnanensis*	Whole plant	China	Cytotoxicity Immunosuppressive
**293**	Reserpine	Corynanthe	*Rauvolfia yunnanensis*	Whole plant	China	Cytotoxicity Immunosuppressive
**294**	(−)-Yohimbine	Corynanthe	*Rauvolfia yunnanensis*	Whole plant	China	Cytotoxicity Immunosuppressive
**295**	Ajmaline	Ajmaline	*Rauvolfia yunnanensis*	Whole plant	China	Cytotoxicity Immunosuppressive
**296**	Mauiensine	Ajmaline	*Rauvolfia yunnanensis*	Whole plant	China	Cytotoxicity Immunosuppressive
**297**	Ajmalicine	Corynanthe	*Rauvolfia yunnanensis*	Whole plant	China	Cytotoxicity Immunosuppressive
**298**	Sitsirikine	Corynanthe	*Rauvolfia yunnanensis*	Whole plant	China	Cytotoxicity Immunosuppressive
**299**	Strictosidinic Acid	Strictosidine	*Rauvolfia yunnanensis*	Whole plant	China	Cytotoxicity Immunosuppressive
**300**	Caboxine B	Corynanthe	*Rauvolfia yunnanensis*	Whole plant	China	Cytotoxicity Immunosuppressive
**301**	Isocaboxine B	Corynanthe	*Rauvolfia yunnanensis*	Whole plant	China	Cytotoxicity Immunosuppressive
**302**	Spegatrine	Sarpagine	*Rauvolfia yunnanensis*	Whole plant	China	Cytotoxicity Immunosuppressive
**303**	19(*S*),20(*R*)-Dihydroperaksine	Sarpagine	*Rauvolfia yunnanensis*	Whole plant	China	Cytotoxicity Immunosuppressive
**304**	Ervataine	Iboga	*Ervatamia yunnanensis*	Stems	China	_$
**305**	Ibogaine	Iboga	*Ervatamia yunnanensis*	Stems	China	_$
**306**	Coronaridine	Iboga	*Ervatamia yunnanensis*	Stems	China	Acetylcholinesterase Inhibition
**307**	Heyneanine	Iboga	*Ervatamia yunnanensis*	Stems	China	_
**308**	Voacangine Hydroxyindolenine	Iboga	*Ervatamia yunnanensis*	Stems	China	_
**309**	Coronaridine Hydroxyindolenine	Iboga	*Ervatamia yunnanensis*	Stems	China	_
**310**	10-Hydroxycoronaridine	Iboga	*Ervatamia hainanensis*	Stems	China	Acetylcholinesterase inhibition
**311**	Voacangine	Iboga	*Ervatamia hainanensis*	Stems	China	Acetylcholinesterase inhibition
**312**	19(*S*)-Heyneanine	Iboga	*Ervatamia hainanensis*	Stems	China	Acetylcholinesterase inhibition
**313**	19(*R*)-Heyneanine	Iboga	*Ervatamia hainanensis*	Stems	China	Acetylcholinesterase inhibition
**314**	Heyneanine Hydroxyindolenine	Iboga	*Ervatamia hainanensis*	Stems	China	Acetylcholinesterase inhibition
**315**	Vobasine	Vobasine	*Ervatamia hainanensis*	Stems	China	Acetylcholinesterase inhibition
**316**	Ervachinine E	Iboga	*Ervatamia chinensis*	Whole plants	China	Cytotoxicity
**317**	Rutaecarpine	Corynanthe	*Ervatamia chinensis*	Whole plants	China	Cytotoxicity
**318**	Ervahainine A	Iboga	*Ervatamia hainanensis*	Leaves and twigs	China	Cytotoxicity
**319**	Ervaoffine A	Iboga	*Ervatamia officinalis*	Leaves and twigs	China	_
**320**	Ervaoffine B	Iboga	*Ervatamia officinalis*	Leaves and twigs	China	_
**321**	Ervaoffine C	Iboga	*Ervatamia officinalis*	Leaves and twigs	China	_
**322**	Ervaoffine D	Iboga	*Ervatamia officinalis*	Leaves and twigs	China	_
**323**	(7*S*)-3-Oxoibogaine Hydroxyindolenine	Iboga	*Ervatamia officinalis*	Leaves and twigs	China	_
**324**	Ibogaine- 5,6-Dione	Iboga	*Ervatamia officinalis*	Leaves and twigs	China	_
**325**	19-*Epi*-5-Oxovoacristine	Iboga	*Ervatamia officinalis*	Leaves and twigs	China	_
**326**	Iboluteine		*Ervatamia officinalis*	Leaves and twigs	China	_
**327**	(7*S*)- Ibogaine Hydroxyindolenine	Iboga	*Ervatamia officinalis*	Leaves and twigs	China	_
**328**	Ibogaline	Iboga	*Ervatamia officinalis*	Leaves and twigs	China	_
**329**	Conopharyngine	Iboga	*Ervatamia officinalis*	Leaves and twigs	China	_
**330**	Voacristine	Iboga	*Ervatamia officinalis*	Leaves and twigs	China	_
**331**	19*S* -Hydroxyibogamine	Iboga	*Ervatamia officinalis*	Leaves and twigs	China	_
**332**	Ibogaine *N*_4_-Oxide	Iboga	*Ervatamia officinalis*	Leaves and twigs	China	_
**333**	3-Oxo-7*r*-Coronaridine Hydroxyindolenine	Iboga	*Ervatamia hainanensis*	Leaves and twigs	China	_
**334**	3*S*-Cyano-7*S*-Coronaridine Hydroxyindolenine	Iboga	*Ervatamia hainanensis*	Leaves and twigs	China	_
**335**	3*R*-Hydroxy-7*S*-Coronaridine Hydroxyindolenine	Iboga	*Ervatamia hainanensis*	Leaves and twigs	China	_
**336**	3*S* -(24*S*-Hydroxyethyl)-Coronaridine	Iboga	*Ervatamia hainanensis*	Leaves and twigs	China	_
**337**	3S -(24*R*-Hydroxyethyl)-Coronaridine	Iboga	*Ervatamia hainanensis*	Leaves and twigs	China	_
**338**	5-Oxo-6*S*-Hydroxycoronaridine	Iboga	*Ervatamia hainanensis*	Leaves and twigs	China	_
**239**	5-Oxo-6*S* -Methoxy-Coronaridine	Iboga	*Ervatamia hainanensis*	Leaves and twigs	China	_
**340**	7S-coronaridine hydroxyindolenine	Iboga	*Ervatamia hainanensis*	Leaves and twigs	China	_
**341**	3-Oxo-7*S*-Coronaridine Hydroxyl Indolenine	Iboga	*Ervatamia hainanensis*	Leaves and twigs	China	_
**342**	5-Oxocoronaridine	Iboga	*Ervatamia hainanensis*	Leaves and twigs	China	_
**343**	3-Oxocoronaridine	Iboga	*Ervatamia hainanensis*	Leaves and twigs	China	_
**344**	Pseudoindoxyl Coronaridine	Iboga	*Ervatamia hainanensis*	Leaves and twigs	China	_
**345**	Ervaoffine E	Iboga	*Ervatamia officinalis*	Leaves and twigs	China	Neuroprotective
**346**	Ervaoffine f	Iboga	*Ervatamia officinalis*	Leaves and twigs	China	Neuroprotective
**347**	Ervaoffine G	Iboga	*Ervatamia officinalis*	Leaves and twigs	China	Neuroprotective
**348**	Lirofoline A	Iboga	*Ervatamia officinalis*	Leaves and twigs	China	Neuroprotective
**349**	Lirofoline B	Iboga	*Ervatamia officinalis*	Leaves and twigs	China	Neuroprotective
**350**	6-Oxo-Ibogaine	Iboga	*Ervatamia officinalis*	Leaves and twigs	China	Neuroprotective
**351**	8-Oxo-Ibogaine Lactam	Iboga	*Ervatamia officinalis*	Leaves and twigs	China	Neuroprotective
**352**	Erchinine A	Iboga	*Ervatamia chinensis*	Roots	China	Antibacterial, Antifungal
**353**	Erchinine B	Iboga	*Ervatamia chinensis*	Roots	China	Antibacterial, Antifungal
**354**	Ervapandine A	Iboga	*Ervatamia pandacaqui*	Leaves and twigs	China	Cytotoxicity
**355**	3*R*-Hydroxyibogaine	Iboga	*Ervatamia pandacaqui*	Leaves and twigs	China	Cytotoxicity
**356**	12-Hydroxyakuammicine *N*_4_-Oxide	Akuammicine	*Ervatamia pandacaqui*	Leaves and twigs	China	Cytotoxicity
**357**	19-*Epi*-Voacristine	Iboga	*Ervatamia pandacaqui*	Leaves and twigs	China	Cytotoxicity
**358**	Taberdivarine I	Iboga	*Ervatamia pandacaqui*	Leaves and twigs	China	Cytotoxicity
**359**	12-Hydroxyakuamicine	Akuammicine	*Ervatamia pandacaqui*	Leaves and twigs	China	Cytotoxicity
**360**	Ervadivamine A	Vobasine-Iboga-Vobasine	*Ervatamia divaricate*	Roots	China	Cytotoxicity
**361**	Ervadivamine B	Vobasine-Iboga-Vobasine	*Ervatamia divaricate*	Roots	China	Cytotoxicity
**362**	19,20-Dihydroervahanine A	Vobasine-Iboga	*Ervatamia divaricate*	Roots	China	Cytotoxicity
**363**	Ibogamine	Iboga	*Ervatamia divaricate*	Roots	China	Cytotoxicity
**364**	Ervatamine	Flabelliformide	*Ervatamia yunnanensis*	Stems	China	_
**365**	20-*Epi*-Ervatamine	Flabelliformide	*Ervatamia yunnanensis*	Stems	China	_
**366**	Dregamine	Vobasine	*Ervatamia yunnanensis*	Stems	China	_
**367**	Tabernaemontanine	Vobasine	*Ervatamia yunnanensis*	Stems	China	_
**368**	Apparicine	Iboga	*Ervatamia yunnanensis*	Stems	China	_
**369**	Isovoacangine	Apparicine	*Ervatamia yunnanensis*	Stems	China	_
**370**	Conodusine A	Iboga	*Tabernaemontana corymbosa*	Stem bark	Malaysia	Cytotoxicity
**371**	Conodusine B	Iboga	*Tabernaemontana corymbosa*	Stem bark	Malaysia	Cytotoxicity
**372**	Conodusine C	Iboga	*Tabernaemontana corymbosa*	Stem bark	Malaysia	Cytotoxicity
**373**	Conodusine D	Iboga	*Tabernaemontana corymbosa*	Stem bark	Malaysia	Cytotoxicity
**374**	Conodusine E	Iboga	*Tabernaemontana corymbosa*	Stem bark	Malaysia	Cytotoxicity
**375**	Apocidine A	Aspidosperma	*Tabernaemontana corymbosa*	Stem bark	Malaysia	Cytotoxicity
**376**	Apocidine B	Aspidosperma	*Tabernaemontana corymbosa*	Stem bark	Malaysia	Cytotoxicity
**377**	Conoduzidine A	Vincamine	*Tabernaemontana corymbosa*	Stem bark	Malaysia	Cytotoxicity
**378**	Tabernamidine A	Vobasine-Iboga	*Tabernaemontana corymbosa*	Stem bark	Malaysia	Cytotoxicity
**379**	Tabernamidine B	Vobasine-Iboga	*Tabernaemontana corymbosa*	Stem bark	Malaysia	Cytotoxicity
**380**	(+)-Catharanthine	Iboga	*Tabernaemontana corymbosa*	Stem bark	Malaysia	Cytotoxicity
**381**	Tabernamine	Vobasine-Iboga	*Tabernaemontana corymbosa*	Stem bark	Malaysia	Cytotoxicity
**382**	19′(*S*)-Hydroxytabernamine	Vobasine-Iboga	*Tabernaemontana corymbosa*	Stem bark	Malaysia	Cytotoxicity
**383**	19′(*R*)-Hydroxytabernamine	Vobasine-Iboga	*Tabernaemontana corymbosa*	Stem bark	Malaysia	Cytotoxicity
**384**	16′-Decarbomethoxyvoacamine	Vobasine-Iboga	*Tabernaemontana corymbosa*	Stem bark	Malaysia	Cytotoxicity
**385**	Isoakuammiline	Corynanthe	*Tabernaemontana litoralis*	Fruits	USA	_
**386**	18-Hydroxypseudovincadifformine	Iboga	*Tabernaemontana litoralis*	Fruits	USA	_
**387**	3,19-Oxidocoronaridine	Iboga	*Tabernaemontana litoralis*	Fruits	USA	_
**388**	Strictosidine	Strictosidine	*Tabernaemontana litoralis*	Fruits	USA	_
**389**	$Tabervarine A	Iboga	*Tabernaemontana divaricate*	Leaves and twigs	China	Cytotoxicity
**390**	$Tabervarine B	Iboga	*Tabernaemontana divaricate*	Leaves and twigs	China	Cytotoxicity
**391**	Vobasidine C	Vobasine	*Tabernaemontana divaricate*	Leaves and twigs	China	Cytotoxicity
**392**	Ervadivaricatine B	Vobasine-Iboga	*Tabernaemontana divaricate*	Leaves and twigs	China	Cytotoxicity
**393**	Pedunculine	Aspidosperma- Aspidosperma	*Tabernaemontana divaricate*	Leaves and twigs	China	Cytotoxicity
**394**	Polyervine	Aspidosperma- Aspidosperma	*Tabernaemontana divaricate*	Leaves and twigs	China	Cytotoxicity
**395**	Flabellipparicine	Flabelliformide-Apparicine	*Tabernaemontana divaricate*	Stems	China	Cytotoxicity
**396**	19,20-Dihydrovobparicine	Vobasine-Apparicine	*Tabernaemontana divaricate*	Stems	China	Cytotoxicity
**397**	10′- Demethoxy-19,20-Dihydrovobatensine D	Vobasine-Iboga	*Tabernaemontana divaricate*	Stems	China	Cytotoxicity
**398**	3′-(2-Oxopropyl)Ervahanine A	Sarpagine-Iboga	*Tabernaemontana divaricate*	Stems	China	Cytotoxicity
**399**	Ervahanine A	Sarpagine-Iboga	*Tabernaemontana divaricate*	Stems	China	Cytotoxicity
**400**	Vobparicine	Vobasine-Apparicine	*Tabernaemontana divaricate*	Stems	China	Cytotoxicity
**401**	19,20-Dihydrotabernamine	Vobasine-Iboga	*Tabernaemontana divaricate*	Stems	China	Cytotoxicity
**402**	19,20-Dihydrotabernamine A	Vobasine-Iboga	*Tabernaemontana divaricate*	Stems	China	Cytotoxicity
**403**	Taberdivarines E	Vobasine-Iboga	*Tabernaemontana divaricate*	Stems	China	Cytotoxicity
**404**	Tubotaiwine	Strychnos	*Tabernaemontana divaricate*	Stems	China	Cytotoxicity
**405**	Hydroxy-3-(2-Oxopropyl) Coronaridine Indolenine	Iboga	*Tabernaemontana divaricate*	Stems	China	Cytotoxicity
**406**	Deoxytubulosine	Corynanthe bisindole	*Tabernaemontana divaricate*	Stems	China	Cytotoxicity
**407**	(3*R*,7*S*,14*R*,19*S*,20*R*)-19-Hydroxypseudovincadifformine	Iboga	*Tabernaemontana bufalina*	Branches and leaves	China	Cytotoxicity
**408**	17-Demethoxy-Hydroisorhyn Chophylline	Corynanthe	*Tabernaemontana bufalina*	Branches and leaves	China	Cytotoxicity
**409**	17-Demethoxy-Isorhynchophylline	Corynanthe	*Tabernaemontana bufalina*	Branches and leaves	China	Cytotoxicity
**410**	Voachalotine	Akuammidine	*Tabernaemontana bufalina*	Branches and leaves	China	Cytotoxicity
**411**	12-Methoxyl-Voaphylline	Aspidosperma	*Tabernaemontana bufalina*	Branches and leaves	China	Cytotoxicity
**412**	Conophylline	Aspidosperma- Aspidosperma	*Tabernaemontana bufalina*	Branches and leaves	China	Cytotoxicity
**413**	5,6-Dioxo-11-Methoxy Voacangine	Iboga	*Tabernaemontana contorta*	Fruits	Cameroon	Anti-inflammatory
**414**	(−)-Apparicin-21-One	Apparicine	*Tabernaemontana contorta*	Fruits	Cameroon	Anti-inflammatory
**415**	Tabernabovine A	Corynanthe bisindole	*Tabernaemontana bovina*	Leaves	China	Anti-inflammatory
**416**	Tabernabovine B	Aspidosperma	*Tabernaemontana bovina*	Leaves	China	Anti-inflammatory
**417**	Tabernabovine C	Iboga	*Tabernaemontana bovina*	Leaves	China	Anti-inflammatory
**418**	Secopleiocarpamine A	Corynanthe	*Rhazya stricta*	Aerial parts	Pakistan	Antifungal
**419**	16,17-Epoxyisositsirikine	Corynanthe	*Rhazya stricta*	Aerial parts	Pakistan	Antifungal
**420**	2-Ethyl-3[2-(3-Ethyl-1,2,3,6-Tetrahydropyridine)Ethyl]-Indole	Secodine	*Rhazya stricta*	Aerial parts	Pakistan	Antifungal
**421**	2-Ethyl-3[2-(3-Ethylpiperidine)Ethyl]-Indole	Secodine	*Rhazya stricta*	Aerial parts	Pakistan	Antifungal
**422**	Tetrahydrosecodine	Secodine	*Rhazya stricta*	Aerial parts	Pakistan	Antifungal
**423**	16,17-Dihydrosecodine	Secodine	*Rhazya stricta*	Aerial parts	Pakistan	Antifungal
**424**	Deacetylakuammilin	Akuammiline	*Rhazya stricta*	Aerial parts	Pakistan	Antifungal
**425**	Rhazimal	Akuammiline	*Rhazya stricta*	Aerial parts	Pakistan	Antifungal
**426**	Strictamine-*N*-Oxide	Akuammiline	*Rhazya stricta*	Aerial parts	Pakistan	Antifungal
**427**	Rhazinaline	Akuammiline	*Rhazya stricta*	Aerial parts	Pakistan	Antifungal
**428**	Rhazinaline *N*_b_-Oxide	Akuammiline	*Rhazya stricta*	Aerial parts	Pakistan	Antifungal
**429**	Akuammicine	Akummicine	*Rhazya stricta*	Aerial parts	Pakistan	Antifungal
**430**	16*R*-*E*-Isositsirikine	Corynanthe	*Rhazya stricta*	Aerial parts	Pakistan	Antifungal
**431**	Dihydrositsirikine	Corynanthe	*Rhazya stricta*	Aerial parts	Pakistan	Antifungal
**432**	Antirhine	Corynanthe	*Rhazya stricta*	Aerial parts	Pakistan	Antifungal
**433**	Vincadifformine *N*_(4)_-Oxide	Aspidosperma	*Rhazya stricta*	Aerial parts	Pakistan	Antifungal
**434**	Eburenine	Aspidosperma	*Rhazya stricta*	Aerial parts	Pakistan	Antifungal
**435**	Winchinine B	Aspidosperma	*Rhazya stricta*	Aerial parts	Pakistan	Antifungal
**436**	Quebrachamine	Aspidosperma	*Rhazya stricta*	Aerial parts	Pakistan	Antifungal
**437**	Strictanol	Aspidosperma	*Rhazya stricta*	Aerial parts	Pakistan	Antifungal
**438**	16-*Epi*-Stemmadenine-*N*-Oxide	Corynanthe	*Rhazya stricta*	Leaves	Saudi Arabia	Cytotoxicity
**439**	Stemmadenine-*N*-Methyl	Corynanthe	*Rhazya stricta*	Leaves	Saudi Arabia	Cytotoxicity
**440**	20-*Epi*-Antirhine	Corynanthe	*Rhazya stricta*	Leaves	Saudi Arabia	Cytotoxicity
**441**	Isopicrinine	Picrinine	*Rhazya stricta*	Leaves	Saudi Arabia	Cytotoxicity
**442**	*Epi*rhazyaminine		*Rhazya stricta*	Aerial parts	Saudi Arabia	Cytotoxicity
**443**	20-*Epi*-sitsirikine		*Rhazya stricta*	Aerial parts	Saudi Arabia	Cytotoxicity
**444**	Strictamine		*Rhazya stricta*	Aerial parts	Saudi Arabia	Cytotoxicity

## Abbreviations

A431	Dermatoma cancer
A-549	Lung cancer
AChE	Acetylcholinesterase
B16F10	Melanogenesis activity
BEN-MEN-1	Meningioma
BGC-823	Human gastric carcinoma
CAL-27	Head and neck squamous cell carcinomas
CCF-STTG1	Astrocytoma
CHG-5	Glioma
CI	Confidence intervals
Detroit-562	Head and neck squamous cell carcinomas
ED_50_	Median effective dose
F.sp.	Forma specialis, abbreviated f. sp., is an informal taxonomic grouping allowed by the International Code of Nomenclature for algae, fungi, and plants
HCT 116	Human colorectal carcinoma
HeLa	Human Gastric cancer
Hep-2	Head and neck squamous cell carcinomas
HepG2	Human hepatocellular
HIF-α	Hypoxia-inducible factor
HL-60	Human myeloid leukemia
HS-1	Dermatona cancer
HS-4	Dermatona cancer
HT-29	Human colorectal carcinoma
IC_50_	Half maximal inhibitory concentration
ID_50_	Median infective dose
IL-1β	Interleukin 1 beta
LNCaP	Human prostate carcinoma
M663	Osteosarcoma cells
MCF-7	Human breast cancer
MDA-MB-231	Human breast adenocarcinoma
MG-63	Osteosarcoma cells
MIAs	Terpenoid indole compounds
MIAs	Monoterpenoid indole compounds
MIC	Minimum inhibitory concentration
NF-kB	Nuclear factor k-light-chain-enhancer of activated B cells
NO	Nitric oxide
PANC-1	Pancreatic cancer
PC-3	Human prostate carcinoma
PGE2	Prostaglandin E2
SAOS-2	Osteosarcoma cell lines
SCC-PKU	Head and neck squamous cell carcinomas
SCL-1	Head and neck squamous cell carcinomas
SGC-7901	Gastric cancer
SHG-44	Human glioma cancer
SK-BR-3	Human breast cancer
SK-MEL-2	Human skin cancer
SMMC-7721	Hepatocellular carcinoma
SOSP-9607	Human Osteosarcoma cell lines
SW480	Human Colon cancer
TCA-83	Head and neck squamous cell carcinomas
TNF-α	Tumor necrosis factor-α
U251	Human glioma cancer
U2-OS	Osteosarcoma cell lines
UMSCC-1	Head and neck squamous cell carcinomas
